# Comparative Analysis of Strain-to-Velocity Conversion Methods for Active-Source DAS Data and Collocated Nodal Stations

**DOI:** 10.3390/s26154673

**Published:** 2026-07-23

**Authors:** Prajwal Panthi, Brady R. Cox

**Affiliations:** Department of Civil and Environmental Engineering, Utah State University, Logan, UT 84322, USA; prajwal.panthi@usu.edu

**Keywords:** distributed acoustic sensing, DAS, nodal stations, strain-to-velocity conversion, fk-rescaling, curvelet, slant-stack, active-source, transfer function

## Abstract

Distributed Acoustic Sensing (DAS) provides dense spatial measurements of the dynamic strain along fiber optic cables, offering high-resolution wave sensing for ground motion monitoring and subsurface imaging applications. However, DAS records the axial strain or strain rate, whereas traditional seismic and engineering ground motion equipment and derived metrics are based on particle displacement, velocity, or acceleration, necessitating reliable strain-to-velocity conversion methods. This study evaluates three widely used conversion approaches: the fk-rescaling, curvelet-based conversion, and slant-stack methods. These approaches are applied to a unique high-energy, near-field, active-source dataset collected at the Birds Landing Site in Sherman Island, California. The dataset includes wavefields generated by a large transmission tower collapse and sledgehammer impacts used for subsurface imaging. The wavefields were recorded simultaneously by a 1.4 km DAS array and 71 collocated nodal stations (NSs). Using 63 DAS–NS pairs, we quantify the strain-to-velocity conversion method performance using amplitude and phase transfer functions (TFs) between DAS-derived and NS particle velocity records, with the root-mean-square error (RMSE) evaluated across three frequency bands: 0.5–100 Hz, 1–10 Hz, and 10–100 Hz. The results show that fk-rescaling provides the most stable amplitude response across both source types, while both the fk-rescaling and slant-stack methods generally yield the best phase agreement. Curvelet-based conversion shows a greater variability and larger RMSE values. All methods yield a poorer amplitude reconstruction at higher frequencies, while the phase content is generally preserved more reliably than amplitudes. Differences between the tower collapse and sledgehammer sources demonstrate the influence of the source characteristics and spatial processing window length on the conversion performance. The findings provide practical guidance for selecting suitable strain-to-velocity conversion methods for active-source DAS applications, particularly where collocated reference sensors are unavailable.

## 1. Introduction

Distributed Acoustic Sensing (DAS) is a powerful and versatile tool for measuring spatially dense dynamic strain fields, with applications in infrastructure health monitoring [1,2,3,4], fault detection [5,6], earthquake early warning [7,8,9], traffic monitoring [10,11], geotechnical site characterization [12,13,14], and subsurface imaging [15,16,17]. By transforming fiber optic cables into dense arrays of strain sensors, DAS provides meter-scale spatial resolution over kilometer-scale distances. This capability is difficult to achieve using traditional ground motion monitoring systems like geophones, seismometers, or accelerometers. However, traditional seismological and engineering ground motion metrics are defined in terms of particle displacement, velocity, or acceleration, while DAS inherently measures the axial strain or strain rate. This fundamental difference requires reliable methods to convert DAS strain measurements into the equivalent particle velocity for a meaningful comparison with traditional seismic sensors and for use in predictive relationships.

Several previous studies on DAS strain-to-velocity conversion methods have focused on applications related to earthquake ground motions recorded with dense DAS channels but with limited coverage from traditional seismic sensors [18,19,20,21,22,23,24]. These studies have reported both successes and challenges associated with accurately converting DAS strain waveforms to equivalent particle-velocity waveforms. Furthermore, these earthquake ground motions have predominantly been acquired at large source-to-receiver distances and generally have small amplitudes and lower frequency bandwidths. In contrast, this study leverages a unique DAS dataset recorded during excitation generated from high-energy, near-field, active sources, including a large transmission tower collapse and sledgehammer impacts used for subsurface imaging. These active-source wavefields were captured by a dense linear array of collocated DAS channels and three-component, high-frequency geophones (i.e., nodal stations). The near-field geometry, and higher-frequency and higher-strain amplitudes recorded in this study provide an opportunity to systematically and quantitatively evaluate several strain-to-velocity conversion methods that have previously been evaluated predominantly on weak-motion seismic waveforms.

The objective of this study is to quantitatively compare the performance of three strain-to-velocity conversion techniques—(1) fk-rescaling [25], (2) curvelet-based conversion [26], and (3) the slant-stack method [27]—by evaluating their ability to reproduce the amplitude and phase characteristics of collocated nodal station particle velocity recordings. These comparisons are qualitatively visualized in the time domain and quantitatively evaluated in the frequency domain using transfer function (TF) analysis and root-mean-square error (RMSE) metrics across multiple frequency bands. This study contributes to the existing body of literature by (i) providing an active-source, high-energy, benchmark dataset for evaluating DAS strain-to-velocity conversion methods, (ii) performing a systematic comparison of widely used conversion techniques, and (iii) quantifying the performance of different conversion methods across multiple frequency ranges using statistically robust metrics.

## 2. Site Description and Data Acquisition

**Figure 1:**
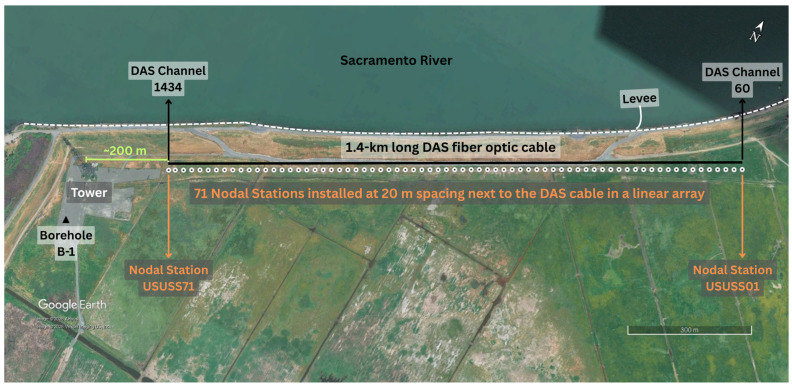
Map of the Birds Landing Site showing the tower location and layout of the 1.4 km long DAS cable (black line) and the linear array of 71 nodal stations (white circles with black centers) relative to the Sacramento River (map imagery: Google Earth).

The data for this study was collected at the Birds Landing Site in Sherman Island, located near the Sacramento–San Joaquin River Delta, north of Antioch, California. The project involved the explosive demolition (controlled collapse) of the tallest electrical transmission tower in the state of California. Due to the close proximity of this tower to the Sacramento River and its flood protection levees along W Sherman Road (Figure 1), the tower owners (Pacific Gas and Electric) wanted to monitor strains induced by the tower collapse while simultaneously using it as an active source for seismic site characterization studies. The site consists of flat deltaic terrain underlain by thick layers of soft peat, clay, and sand. A borehole (B-1) drilled near the tower and advanced down to a depth of 40 m revealed approximately 1.5 m of sandy lean clay over more than 7 m of very soft fibrous peat, underlain by alternating layers of soft fat clay, lean clay, and dense sand.

An approximately 1.4 km long fiber optic cable and 71 nodal stations were installed in a linear array parallel to the levee to record wavefields and monitor strains induced along the toe of the levee and surrounding soils during the tower collapse (Figure 1). Due to the need to stay out of the collapse radius of the tower, the closest nodal station (USUSS71) and DAS channel (1434) were both situated approximately 200 m away from the base of the tower. Herein, this side of the DAS array is referred to as the “tower-end” (i.e., the end near the tower). The farthest nodal station (USUSS01) and DAS channel (60) were situated 1.4 km away from the nearest channel. Herein, this side of the DAS array is referred to as the “IU-end” (i.e., the end where the DAS interrogator unit—IU—was placed).

The DAS fiber optic cable used for this project was a tight-buffered, single-strand, single-mode cable manufactured by TiniFiber (Lindenhurst, NY, USA). It has a nominal outer diameter of 3 mm, a tough but pliable UV-resistant outer plastic jacket, Kevlar, hydro-yarns, and a metal braid for strength and improved coupling, and a fiber optic refractive index of 1.4677. It was installed by hand in a trench approximately 15 to 20 cm deep that was excavated using a walk-behind landscaping trencher. The trench was then backfilled and compacted by hand to ensure effective coupling between the cable and the ground.

The fiber optic cable was connected to an Optasense (Roanoke, VA, USA) ODH4+ interrogator unit (IU) for data acquisition. The IU was set to acquire data using a gauge length (GL) of 2.0419 m, a spatial sampling interval of 1.017 m, a wavelength of 1550 nanometers, and a pulse rate of 40 kHz. Using tap tests, the channels corresponding to the array limits on the IU- and tower-ends of the cable were identified as Channel 60 and Channel 1434, respectively (see Figure 1). The IU was GPS-synchronized to Coordinated Universal Time (UTC) and was programmed to save the continuous time records in minute-long .h5 files, which were downsampled to 1 kHz prior to saving to reduce the file size and facilitate processing.

SmartSolo (Calgary, AB, Canada) IGU-16HR 3C nodal stations (NSs) were placed every 20 m along the DAS cable. This spacing resulted in 71 NSs installed in a linear array offset approximately 30 cm from the cable. Each NS was buried to its top in a hand-dug hole, oriented towards magnetic north, and backfilled with native soil for good coupling. The stations were GPS-synchronized and configured with a 250 Hz sampling rate. The NS array extends from USUSS01 at the IU-end (collocated with DAS Channel 60) to USUSS71 at the tower-end (collocated with DAS Channel 1434), resulting in a total of 71 collocated DAS channel–NS pairs.

## 3. Data Preprocessing

The DAS and NS were used to record wavefields from two active sources: (1) the controlled collapse of the transmission tower, and (2) vertical sledgehammer impacts on a round aluminum strike plate imposed every 10 m along the DAS array. Note that the dense configuration of sledgehammer impacts was intended to allow for 2D subsurface imaging along the entire length of the DAS array using MASW and full waveform inversion methods [15,16], although that work has not yet been completed. For each active source, a consistent spatial processing window was established around every DAS channel–NS pair to enable uniform comparisons across the array.

**Figure 2:**
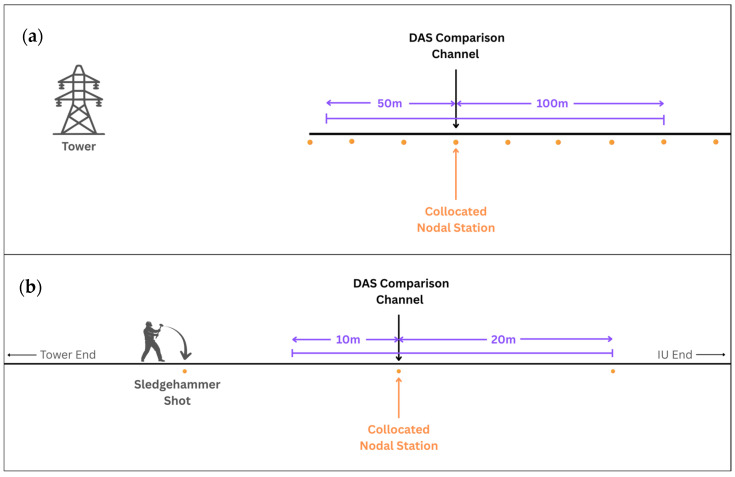
Schematic showing the spatial processing window used for analysis of a DAS channel–nodal station pair for (**a**) the tower collapse, and (**b**) a sledgehammer shot as the source.

For the tower collapse source, a 150-channel (~150 m) DAS segment was extracted around each DAS–NS pair, extending 50 m to the left and 100 m to the right, as illustrated in Figure 2a. This asymmetric window was selected to account for the dominant wave propagation direction from the tower-end to the IU-end of the array, and to provide a sufficient spatial aperture for the stable application of the strain-to-velocity conversion methods. Out of 71 DAS–NS pairs, 8 pairs located near the ends of the array and corresponding to nodal stations USUSS01–05 and USUSS69–71 were excluded from comparisons due to the geometric limitations necessitated by using a 150 m DAS segment length. Thus, only 63 of the 71 DAS–NS pairs could be used in the strain-to-velocity comparisons for the tower collapse source. For all DAS channels within each spatial window, a fixed 60 s time window was extracted around the tower-impact event from 19 March 2025 at 20:45:47 to 20:46:47 UTC. This time window included approximately 3 s of a pre-trigger delay and approximately 57 s of “listen” time.

For the sledgehammer data, a shot positioned 20 m to the left of each DAS–NS pair was consistently selected as the source location. A smaller, 30-channel (~30 m) DAS segment was then extracted around each DAS–NS pair, extending 10 m to the left and 20 m to the right, as shown in Figure 2b. This asymmetric window maintains the same wavefield propagation direction as used for the tower collapse and preserves an offset of 10 m from the sledgehammer to the nearest channel in the DAS segment as a means to mitigate near-field effects. Eight pairs corresponding to nodal stations USUSS01, 02, 06, 07, 51, 52, 70, and 71 were excluded due to the geometric limitations at the ends of the DAS array necessitated by using a 30 m DAS segment or because of noisy records caused by not holding still enough during sledgehammer blows. Thus, once again, only 63 of the 71 DAS–NS pairs could be used in the strain-to-velocity comparisons for the sledgehammer source. For each sledgehammer shot, a 4 s time window with a 1 s pre-trigger delay and 3 s of listen time was used for analysis.

The DAS and NS records were trimmed to identical time windows around each active-source event. The DAS data, which was originally saved by the ODH4+ IU in raw phase units of radians per 2π divided by a factor of 2^16^, was converted into units of absolute strain using Equation (1) [2]:

(1) $$\;\varepsilon_{DAS}=\frac{\Lambda d\phi }{4\pi n\xi g}$$

where $\varepsilon_{DAS}$ is the average longitudinal/normal strain in the direction of the fiber over a gauge length g, Λ is the average optical wavelength of the laser used by the IU (1550 nm), dϕ is the phase change measured by the DAS system, n is the group refractive index of the sensing fiber (1.4677), and ξ is the photoelastic scaling factor for the longitudinal strain in the optical fiber (0.78). The DAS strain records were first filtered using a sixth-order 0.5–125 Hz bandpass filter and then downsampled from 1 kHz to 250 Hz to match the sampling frequency of the NS records. A complete instrument response correction was applied to the NS recordings to obtain the particle velocity in units of m/s, and then the two horizontal components of the NS were mathematically combined/rotated to provide a single horizontal component in line with the DAS array to obtain a consistent directionality between the datasets. Subsequently, the NS records were filtered using a sixth-order 0.5–125 Hz bandpass filter, identical to the DAS records. The 0.5 Hz low-frequency cutoff was chosen to ensure that the NS waveforms used as a reference to evaluate the DAS strain-to-velocity conversion methods were reliable over the selected frequency bandwidth. Research has shown that these SmartSolo IGU-16HR stations compare favorably with broadband instruments down to approximately 0.2 Hz once the instrument response has been accounted for [28]. Given that our comparisons only extend down to 0.5 Hz, we believe using the NS as a reference for this study is reasonable. Additionally, all frequency domain evaluations in this study are reported only up to 100 Hz, which is approximately consistent with a guard-band factor of 2.56 to account for the imperfect roll-off of the 125 Hz digital filter. For our 250 Hz sampling rate, this corresponds to 250/2.56 = 97.7 Hz ≈ 100 Hz.

## 4. Strain-to-Velocity Conversion Methodology

This study evaluates three widely used approaches for converting DAS strain measurements into particle velocity: (1) fk-rescaling, (2) curvelet-based conversion, and (3) the slant-stack method. Each conversion method relies on different assumptions regarding wavefield coherence, propagation direction, and apparent phase velocity estimation, which can influence the reconstructed particle velocity waveforms. All implementations are carried out using the open-access Python toolbox DASPy v1.2.2 [29]. The strain-to-velocity conversion tool (strain2vel.py) integrated in DASPy is used for this analysis, and each method has its own function and required input parameters associated with it.

### 4.1. fk-Rescaling Method

The fk-rescaling method [25,30] operates in the frequency–wavenumber (*f*–*k*) domain by converting DAS strain measurements to particle velocity using the relationship between the temporal frequency (f) and spatial wavenumber (k). For a propagating plane wave, the longitudinal strain (ε) and particle velocity (u′) in the longitudinal direction are related through the velocity (v) of the wave exciting particle motion in the longitudinal direction according to Equation (2):

(2) $$\;\;\;\varepsilon =\frac{u\prime }{v}$$

where v is the wave velocity of the dominant wave-type causing the longitudinal strain. For surface-based DAS measurements of waveforms emanating from a vertically oriented active source on the ground surface, the dominant wave-type is assumed to be Rayleigh-type surface waves. Thus, v is an apparent Rayleigh wave phase velocity ($v_{R}$) that can be calculated according to Equation (3):

(3) $$v_{R}=f\cdot \frac{2\pi }{k}=f\cdot \lambda$$

where λ is the frequency-dependent Rayleigh wavelength. The term “apparent” velocity is used because the true Rayleigh wave phase velocity at each frequency may or may not be resolved due to factors such as near-field effects and/or the possibility of measuring waves that are not propagating directly in line with the DAS array. The measured DAS strain ($\varepsilon_{DAS}$) can be converted to particle velocity by scaling the frequency-dependent strain amplitudes by the frequency-dependent apparent Rayleigh wave phase velocity in the *f*–*k* domain according to Equation (4):

(4) $$u^{\prime }=\varepsilon_{DAS}\cdot v_{R}=\varepsilon_{DAS}\cdot f\cdot \frac{2\pi }{k}$$

The fk-rescaling method assumes that the wavefield can be locally approximated as coherent plane-wave propagation over the selected spatial aperture of the array. In DAS applications, this assumption is often reasonable for surface-wave-dominated wavefields propagating along relatively linear fiber geometries. Lindsey et al. [25] showed that DAS preserves the phase characteristics of seismic waves over a wide frequency range, although the amplitude response may vary due to factors such as the coupling conditions, gauge length effects, and array geometry. Boullenger et al. [30] further demonstrated that *f*–*k* domain conversion produced DAS-derived velocity waveforms that showed a good overall agreement in amplitude and phase relative to collocated accelerometer records, although the peak amplitudes were initially underestimated by approximately 30–40% prior to further correction.

In DASPy, this conversion is implemented using the *fk_rescaling* function, which applies velocity-dependent filtering and scaling within the *f*–*k* domain. It requires inputs for parameters such as channel spacing (dx), sampling frequency ($f_{s}$), space and time end tapers (taper), maximum frequency retained ($f_{max}$), minimum wavenumber cutoff range ($k_{min}$), maximum allowed velocity range ($v_{max}$), and *fk* mask boundary taper (edge). Among these, the most influential parameters were found to be $v_{max}$, which defines the upper bound of surface-wave phase velocities retained in the conversion, and $k_{min}$, which controls the inclusion of long-wavelength components.

Prior to applying the fk-rescaling conversion, the DAS strain data was trimmed into local spatial segments around each DAS–NS pair, as described earlier (refer to Figure 2a,b). Then, to guide the selection of the parameters used for conversion, surface wave dispersion data was extracted from DAS records using both active sources (tower collapse and sledgehammer) and passive ambient noise using the open-source Python package *swprocess* [31]. The dispersion data extracted from the active and passive DAS waveforms is included in the Accompanying Electronic Supplement (Figure S1), and it was used to guide the selection of $v_{max}$ and $k_{min}$. The value of $v_{max}\;$was selected to be slightly above the upper bound of the reliable dispersion data. For example, for the tower impact source, where phase velocities were observed up to approximately 750 m/s, a tapered range of $v_{max}$ (750, 800) m/s was adopted. Similarly, $k_{min}$ was selected based on the maximum wavelength ($\lambda_{max}$) of the reliable dispersion data using the relation $k_{min}=\frac{2\pi }{\lambda_{max}}\;$. For the tower impact source, $\lambda_{max}$ was around 2000 m, which yields $k_{min}=\frac{2\pi }{2000}=\;$0.003 $m^{-1}$. Thus, a slightly higher range (0.002, 0.0017) $m^{-1}$ was chosen. This procedure was repeated using the sledgehammer dispersion data to inform the $v_{max}$ and $k_{min}$ parameters for the sledgehammer source as well.

Sensitivity studies were performed for all the required fk-rescaling parameters as a means to investigate the optimal values for use across the entire DAS array. These studies were performed thoroughly and quantitatively for each of the parameters. Additional details regarding this input parameter sensitivity study will be discussed later in the paper after the background information on transfer functions and root-mean-square error (RMSE) metrics have been discussed. The optimal set of parameters used in the fk-rescaling conversion for each source are summarized in Table 1. Additionally, the range of values considered for the most influential parameters, along with the selected optimal values, are provided in Table S1 of the Electronic Supplement.

### 4.2. Curvelet-Based Conversion

The curvelet-based conversion method [26] decomposes the DAS wavefield into components known as curvelets, which are localized in space, time, propagation direction, and frequency content. Unlike conventional Fourier-domain approaches that represent the wavefield using globally distributed sinusoidal functions, the curvelet transform represents the DAS wavefield as a summation of many small directional wavefield packets. Mathematically, the DAS wavefield may be approximated as follows:

(5) $$Wavefield\approx \sum c_{i}\phi_{i}$$

where $c_{i}$ is a coefficient representing the contribution of a particular directional wavefield packet, and $\phi_{i}$ is a curvelet basis function characterized by a specific spatial position, scale, and orientation. Each curvelet basis function is localized in frequency, wavenumber, and propagation direction, and therefore represents waves travelling within a particular apparent velocity range. For example, functions associated with high frequencies and low wavenumbers correspond to higher apparent velocities, whereas functions associated with lower frequencies and higher wavenumbers correspond to lower apparent velocities. Consequently, each curvelet basis function represents a localized wavefield component associated with a specific directional and velocity range in the *f*–*k* domain.

In the curvelet-based strain-to-velocity conversion implemented in DASPy, each curvelet coefficient is multiplied by the representative apparent velocity associated with its corresponding curvelet basis function. Specifically, the median apparent velocity represented by each curvelet basis function in the *f*–*k* domain is used for the conversion. Thus, similar to Equation (6) that represents the strain–velocity relationship used in the fk-rescaling method, the particle velocity is estimated by scaling the DAS strain values by their corresponding median apparent phase velocities. However, unlike fk-rescaling, which assumes a globally coherent wavefield over the entire spatial window, the curvelet method performs this conversion locally in terms of the spatial position, propagation direction, and scale. This localized directional decomposition makes the curvelet method theoretically well-suited for DAS wavefields containing scattered energy, interfering arrivals, directional noise, curved wavefronts, or multidirectional propagation. Additionally, the method may be advantageous for DAS deployments with non-linear fiber geometries where the wave propagation characteristics vary spatially along the array. Atterholt et al. [26] demonstrated that curvelet-based processing can improve the directional wavefield separation and suppress the incoherent noise in DAS arrays, enabling the improved reconstruction of coherent seismic arrivals used for the DAS strain-to-ground-motion conversion. However, for near-field active-source datasets collected with a linear DAS array, the dominant wave propagation is likely to be coherent and unidirectional. Therefore, the benefits of localized directional decomposition may not be realized under such conditions.

In curvelet decomposition, a term “scale” is used, which refers to the spatial size or wavelength content of the represented wavefield packets. Coarser-scale curvelets represent broader, lower-frequency, long-wavelength wavefield packets, whereas finer-scale curvelets represent smaller, higher-frequency, short-wavelength packets. DASPy implements this conversion using the *curvelet_conversion* function, which requires inputs for parameters such as dx, $f_{s}$, space and time zero-padding (pad), starting scale level (*scale*_*begin*), number of decomposition scales (nbscales), and number of angular directions (nbangles) [29]. The parameter nbscales controls the total number of decomposition levels used to represent the wavefield. Increasing the number of scales allows the representation of a broader range of wavelength and frequency content, although excessively large values may increase the computational cost and sensitivity to noise. Conversely, too few scales may result in the inadequate multiscale representation of the wavefield. The parameter *scale*_*begin* controls which coarse-scale components are included in the decomposition and can therefore influence the suppression of very-low-frequency or -long-wavelength artifacts. The parameter nbangles, specified in multiples of 4, controls the directional resolution of the decomposition. Increasing this value improves the directional discrimination between propagating arrivals but also increases the computational expense and memory requirements. Among these parameters, nbscales, *scale*_*begin*, and nbangles were found to most strongly influence the converted velocity waveforms. Similar to the fk-rescaling method, rigorous sensitivity studies were performed for each of these parameters as a means to obtain the best agreement between the DAS and NS waveforms, and an optimal set of parameters for each source type was obtained, as summarized in Table 1.

### 4.3. Slant-Stack Method

The slant-stack method [27,32] converts DAS strain to particle velocity by estimating the apparent phase slowness of locally coherent wavefields in the time-slowness domain. Unlike the fk-rescaling method, which estimates the apparent Rayleigh wave phase velocity over relatively large spatial and temporal windows in the *f*–*k* domain, the slant-stack method estimates the apparent slowness (inverse of the velocity) locally and continuously as a function of time. This allows the method to adapt to changing wave propagation characteristics, varying arrival directions, interfering wavefields, and time-dependent apparent velocities.

From Equation (6), the particle velocity can be expressed in terms of the longitudinal DAS strain and slowness (p) as follows:

(6) $$u^{\prime }=\varepsilon_{DAS}\cdot v_{R}=\frac{\varepsilon_{DAS}}{p}\;$$

where slowness (p) is the reciprocal of apparent phase velocity ($v_{R}$). Lior et al. [27] emphasized that this strain–velocity relationship is valid under the assumption that the locally recorded wavefield can be approximated as a coherent plane wave.

The slant-stack method first selects a local spatial aperture centered on the target DAS channel. Within this aperture, a range of trial slowness values is tested. For each trial slowness, the neighboring DAS traces are shifted according to the expected wave arrival timing for that slowness and a semblance value is calculated to measure how well the shifted traces align. If the tested slowness matches the actual slowness of the dominant wavefield, the shifted traces align well and produce a high semblance value. The slowness corresponding to the maximum semblance is then selected as the apparent phase slowness at that time step. Repeating this process through time produces a time-dependent slowness series, p(t). Because the estimated slowness may fluctuate abruptly due to noise, scattered waves, interfering arrivals, or changes in propagation direction, the slowness series is smoothed before conversion.

Finally, the smoothed apparent slowness is used in Equation (6) to convert the DAS strain waveform to particle velocity. This follows the general slant-stack conversion framework described by Lior et al. [27], where a local semblance-based slowness estimation is used to convert DAS strain records to ground motion. In DASPy, this method is implemented using the slowness and *slant*_*stacking* functions, which require inputs for parameters such as dx, $f_{s}$, maximum slowness bound (slm), slowness step (sls), stack length in number of channels (*L*_*stack*), and pass band low corner and high corner frequencies (frqlow & frqhigh) [29]. Here, an important thing to note is that the parameter *L*_*stack* does not represent the total DAS segment length shown in Figure 2a,b. Instead, it represents the number of neighboring channels used on each side of the target channel, resulting in a total aperture of 2*L*_*stack* + 1 channels. For example, using *L*_*stack* = 50 channels for the tower collapse results in a local aperture of 101 channels centered on the target channel, whereas *L*_*stack* = 10 channels for the sledgehammer shots results in a local aperture of 21 channels. Larger *L*_*stack* values generally improve the slowness stability and resolution by including more traces in the semblance calculation, but they reduce the spatial locality and may violate the assumption that the wavefield remains locally coherent. Smaller *L*_*stack* values better preserve the local wavefield behavior but may produce less stable slowness estimates. The parameter slm defines the maximum bound of slowness searched, and therefore controls the range of apparent velocities considered during conversion. The parameter sls controls the slowness sampling interval; smaller values provide a finer slowness resolution but increase the computation time and may make the estimate more sensitive to noise.

Out of all the parameters used in this method, the most influential were found to be *L*_*stack*, slm, and sls. Similar to the prior methods, sensitivity studies were performed for each of these parameters, and an optimal set of parameters for each source was obtained, which are summarized in Table 1.

## 5. Results and Discussion

For a comprehensive qualitative and quantitative comparison of the three strain-to-velocity conversion methods, time-domain waveforms, amplitude TFs, and phase TFs were generated for all DAS–NS pairs using the optimal parameter sets summarized in Table 1. The results are presented separately for the tower collapse and the sledgehammer sources, and the performance of each method is evaluated in terms of the visual waveform agreement, visual TF behavior, and RMSE-based metrics across multiple TF frequency bands. This enables a qualitative and quantitative assessment of how effectively each conversion method reconstructs the particle velocity amplitude and phase content from DAS strain measurements relative to the collocated NS recordings.

### 5.1. Tower Collapse Source

**Figure 3:**
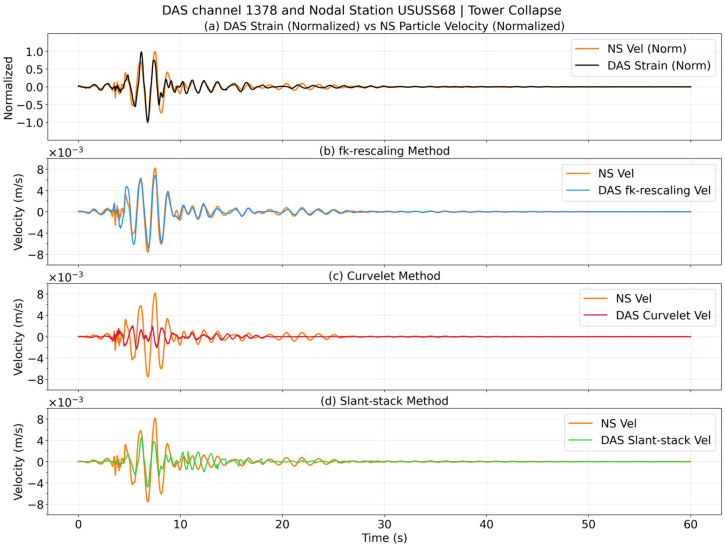
Time-domain waveforms recorded during the tower collapse at the closest usable DAS–NS pair to the tower (DAS channel 1378 and Nodal Station USUSS68). Provided are (**a**) the NS particle velocity time history normalized by its peak particle velocity and the DAS strain time history normalized by its peak strain value, and (**b**–**d**) NS particle velocity time history versus DAS particle velocity time history converted from strain using three methods: (**b**) fk-rescaling, (**c**) curvelet, and (**d**) slant-stack.

Figure 3 presents waveform comparisons for the closest usable DAS–NS pair to the tower (DAS channel 1378 and Nodal Station USUSS68) for the tower collapse source. In Figure 3a, the DAS strain waveform and the NS particle velocity waveform, both normalized by their respective peak amplitudes, are shown to allow a visual comparison of waveform shapes in terms of the relative amplitude and relative timing (i.e., phase) prior to any strain-to-velocity conversion. This amplitude-normalized comparison between the NS particle velocity and DAS strain provides a baseline for evaluating the three DAS strain-to-velocity conversion approaches later. As observed in Figure 3a, the normalized waveforms agree very well with one another, with some visual differences in the higher-frequency components of the signals. This is consistent with prior studies showing that the DAS recordings exhibit a strong similarity to collocated geophone observations. For example, Wang et al. [20] demonstrated a strong correspondence between the DAS strain rate measurements and collocated geophone particle velocity records in terms of waveform similarity and arrival timing using the PoroTomo dataset. In addition to the wave propagation effects, the DAS amplitude behavior can also be influenced by spatial variations in fiber-ground coupling and installation conditions along the array [25]. These effects can contribute to the local variability in amplitude reconstruction between DAS and collocated nodal stations, particularly at higher frequencies where DAS measurements become increasingly sensitive to small-scale heterogeneity and coupling differences. The Electronic Supplement provides waterfall-style waveform comparison plots for all amplitude-normalized DAS–NS pairs recorded during the tower collapse (Figures S2–S4).

The remaining panels in Figure 3 compare the tower collapse waveforms for this DAS–NS pair in terms of the particle velocity, with the DAS strain-to-velocity converted waveforms from the fk-rescaling, curvelet-based conversion, and slant-stack methods shown in Figure 3b–d, respectively. Overall, the fk-rescaling method produces the closest visual agreement with the NS waveform, reproducing amplitudes of the major peaks and troughs while maintaining a good phase alignment. The curvelet-based method shows the largest visual mismatch in both amplitude and phase, while the slant-stack method gives an intermediate performance, with a generally good phase agreement but less consistent amplitude scaling. The Electronic Supplement provides waterfall-style comparison plots for all DAS–NS pairs and all three strain-to-velocity conversion methods for waveforms recorded during the tower collapse (Figures S5–S13).

To quantify the waveform agreement as a function of frequency, the amplitude and phase TFs were computed for each DAS–NS pair. First, the converted DAS and NS particle velocity waveforms were transformed to the frequency domain via Fourier transformation. Then, the amplitude and phase TFs (TF_amp_ and TF_phase_, respectively) between the DAS-derived particle velocity and the collocated NS particle velocity were calculated using Equations (7) and (8):

(7) $$TF_{amp}=\frac{FAS_{DAS}}{FAS_{NS}}\;\;\;\;$$

(8) $$TF_{phase}=FPS_{DAS}-FPS_{NS}$$

where FAS is the Fourier amplitude spectrum of the DAS or NS time history (i.e., the square root of the sum of the squares of the real and imaginary parts) and FPS is the Fourier phase spectrum of the DAS or NS (i.e., the inverse tangent of the imaginary part divided by the real part). For a perfect amplitude agreement, the TF_amp_ should equal 1.0 across all frequencies, while, for a perfect phase agreement, the TF_phase_ should equal 0° across all frequencies. Deviations from these reference values indicate a frequency-dependent mismatch between the DAS and NS waveforms.

**Figure 4:**
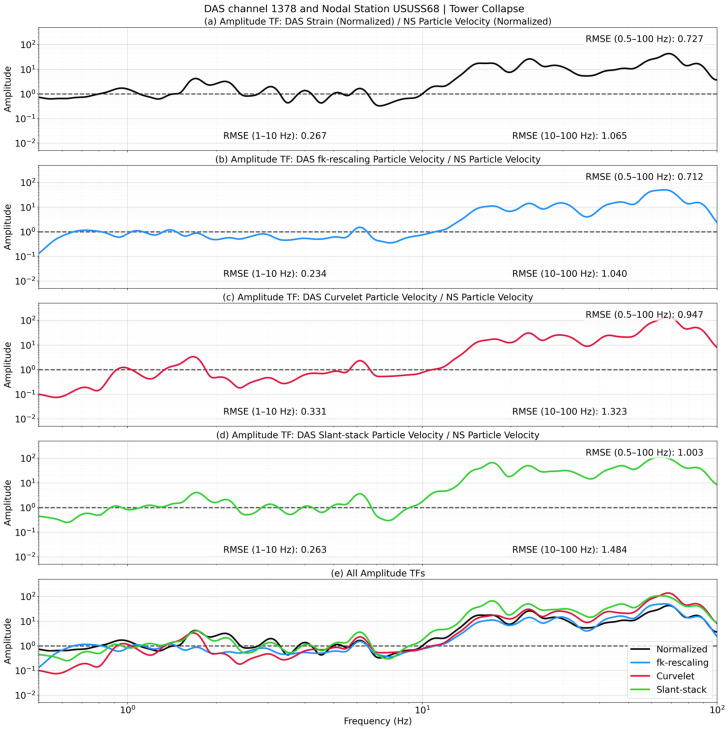
Amplitude TFs calculated for waveforms recorded during the tower collapse at the closest usable DAS–NS pair to the tower (DAS channel 1378 and Nodal Station USUSS68). Provided are (**a**) normalized DAS strain to normalized NS particle velocity TF, (**b**) fk-rescaling DAS particle velocity to NS particle velocity TF, (**c**) curvelet DAS particle velocity to NS particle velocity TF, (**d**) slant-stack DAS particle velocity to NS particle velocity, and (**e**) all amplitude TFs overlaid. RMSE values are reported for three frequency bands, 0.5–100 Hz, 1–10 Hz, and 10–100 Hz, using the log-amplitude RMSE metric.

**Figure 5:**
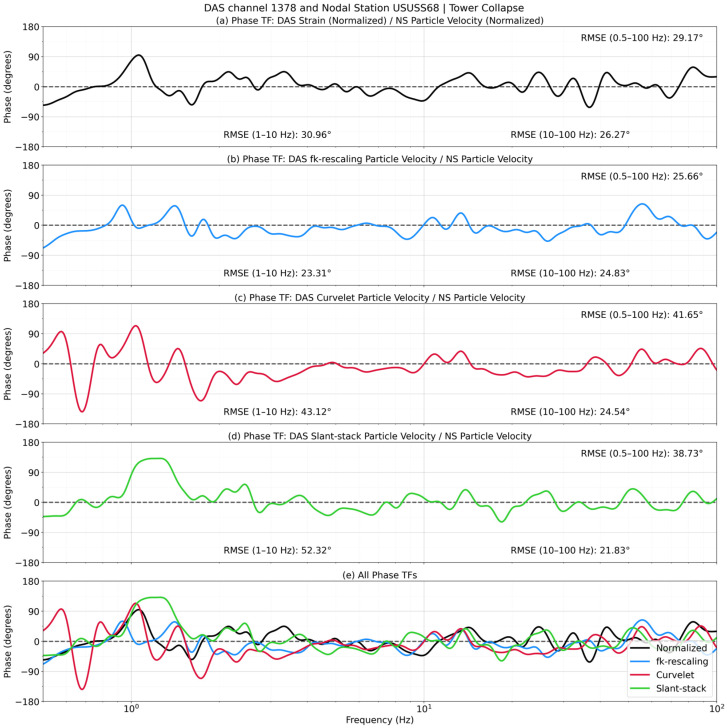
Phase TFs calculated for waveforms recorded during the tower collapse at the closest usable DAS–NS pair to the tower (DAS channel 1378 and Nodal Station USUSS68). Provided are (**a**) normalized DAS strain to normalized NS particle velocity TF, (**b**) fk-rescaling DAS particle velocity to NS particle velocity TF, (**c**) curvelet DAS particle velocity to NS particle velocity TF, (**d**) slant-stack DAS particle velocity to NS particle velocity, and (**e**) all phase TFs overlaid. RMSE values are reported for three frequency bands, 0.5–100 Hz, 1–10 Hz, and 10–100 Hz, using the phase RMSE metric.

Figure 4 and Figure 5 show the amplitude and phase TFs, respectively, over the frequency range of 0.5 to 100 Hz for the same DAS–NS pair waveforms shown in Figure 3. Subplot (a) in Figure 4 and Figure 5 represents the TFs calculated between the normalized DAS strain and NS particle velocity waveforms, and subplots (b)–(d) represent the TFs calculated between the strain-to-velocity converted DAS waveforms and the NS particle velocity waveforms for the three DAS strain-to-velocity conversion methods, while subplot (e) overlays all cases to facilitate a direct comparison. These figures show that the agreement in amplitude and phase varies substantially with frequency, and also across conversion methods. In particular, the behavior below 10 Hz differs noticeably from the behavior above 10 Hz for the amplitude TFs shown in Figure 4, with a better agreement (i.e., TF_amp_ closer to 1.0) below 10 Hz. [We note here that the amplitude TFs are plotted on a log-amplitude scale].

In order to quantify the performance between conversion methods, three frequency bands were selected for evaluation: (1) 0.5–100 Hz (overall performance), (2) 1–10 Hz (lower-frequency band), and (3) 10–100 Hz (higher-frequency band). RMSE values were then calculated over each frequency band using Equations (9) and (10) to quantify the strain-to-velocity conversion method performance in terms of amplitude and phase, respectively:

(9) $$RMSE_{amp}=\;\;\sqrt{\frac{1}{N}\;\sum_{i=1}^{N}\log_{10}(TF_{amp}{\left(f_{i}\right))}^{2}}$$

(10) $$RMSE_{phase}=\sqrt{\frac{1}{N}\;\sum_{i=1}^{N}{\left(TF_{phase}\left(f_{i}\right)\right)}^{2}}$$

where N is the total number of frequency samples within the selected band, i is a counter from 1 to N, and $f_{i}$ is the frequency in Hz. For the amplitude, a logarithmic metric was used because amplitude TFs can vary over orders of magnitude. Thus, an RMSE_amp_ value of 1.0 indicates that the DAS particle velocity amplitude is a factor of 10 different than the NS particle velocity amplitude. Lower RMSE values indicate TF_amp_ ratios that are closer to 1.0. For the phase, lower RMSE values indicate TF_phase_ ratios that are closer to 0°.

For the example DAS–NS pair shown in Figure 4 and Figure 5, the RMSE values for each frequency band and strain-to-velocity conversion method are reported within the corresponding subplots. In Figure 4, the fk-rescaling and slant-stack methods perform the best in the 1–10 Hz range, with RMSE_amp_ values of 0.234 and 0.263, respectively, suggesting a good amplitude reconstruction over lower frequencies. In fact, both of these strain-to-velocity conversion methods resulted in a slightly better amplitude agreement than simply normalizing the DAS strain and NS velocity waveforms by their respective maximum amplitudes, which yielded an RMSE_amp_ value of 0.267 over the 1–10 Hz range. The curvelet method departs more from TF amplitude unity and shows larger fluctuations in the 1–10 Hz band. In the 10–100 Hz band, all methods show an increased deviation from TF amplitude unity, indicating an amplification bias in the converted DAS response at higher frequencies. However, the fk-rescaling method performs the best over the 10–100 Hz band with an RMSE_amp_ value of 1.04 and the best over the entire 0.5–100 Hz band with an RMSE_amp_ value of 0.712. [We note here that an RMSE_amp_ value of 0.712 is equivalent to an average TF amplitude ratio of 10^0.712^ = 5.15.] All of the amplitude TFs for this DAS–NS pair are compared in Figure 4e.

The phase TFs in Figure 5 show comparatively flatter/more stable trends across frequency than the amplitude TFs. However, in general, the phase TFs indicate a better phase alignment at higher frequencies than lower frequencies. The fk-rescaling method again performs best overall, with the lowest RMSE_phase_ value of 25.66° across the entire 0.5–100 Hz bandwidth. It also has the lowest RMSE_phase_ value, by far, over the 1–10 Hz bandwidth at 23.31°. The other methods have significantly higher RMSE_phase_ values in the 1–10 Hz band than in the 10–100 Hz band, and the curvelet method has the highest RMSE_phase_ value of 41.65° across the entire 0.5–100 Hz bandwidth. The slant-stack method performs slightly better than the fk-rescaling method over the higher-frequency band.

At this point, a brief aside in the narrative is warranted to further explain the sensitivity studies used to choose the optimum strain-to-velocity conversion parameters provided in Table 1 and discussed in Section 4. The RMSE_amp_ and RMSE_phase_ values obtained from three DAS–NS pairs distributed across the length of the array (one at each end of the DAS array, and one in the center) were used in the sensitivity studies to identify the most impactful set of parameters and their optimal values for each strain-to-velocity conversion method. Numerous parameter values for each conversion method were considered, and the RMSE_amp_ and RMSE_phase_ values were tracked to find the optimal set of parameters. All waveform comparisons presented in this paper were made after choosing and applying the optimal set of parameters for each conversion method determined by minimizing the RMSE errors for the TFs.

**Figure 6:**
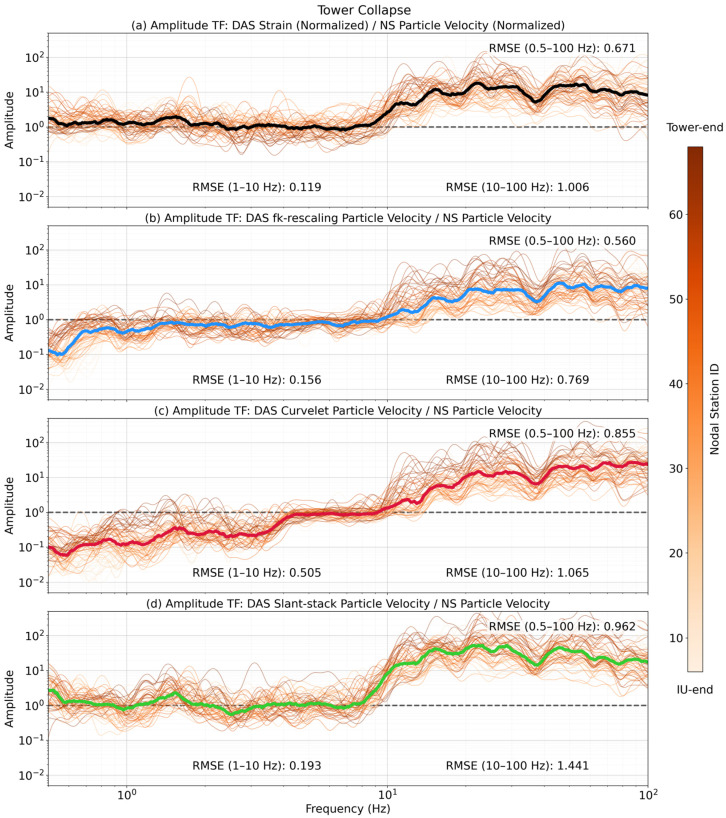
Amplitude TFs for all 63 DAS–NS pairs and the median TF for each strain-to-velocity conversion method for waveforms recorded during the tower collapse. Individual TF curves are color-coded by NS ID, varying from light orange at the IU-end to dark orange at the tower-end. Median TF curves calculated across all pairs are shown for (**a**) normalized waveforms, (**b**) fk-rescaling, (**c**) curvelet, and (**d**) slant-stack. RMSE values for the median TF are shown in each subplot for three frequency bands, 0.5–100 Hz, 1–10 Hz, and 10–100 Hz, using the log-amplitude RMSE metric.

**Figure 7:**
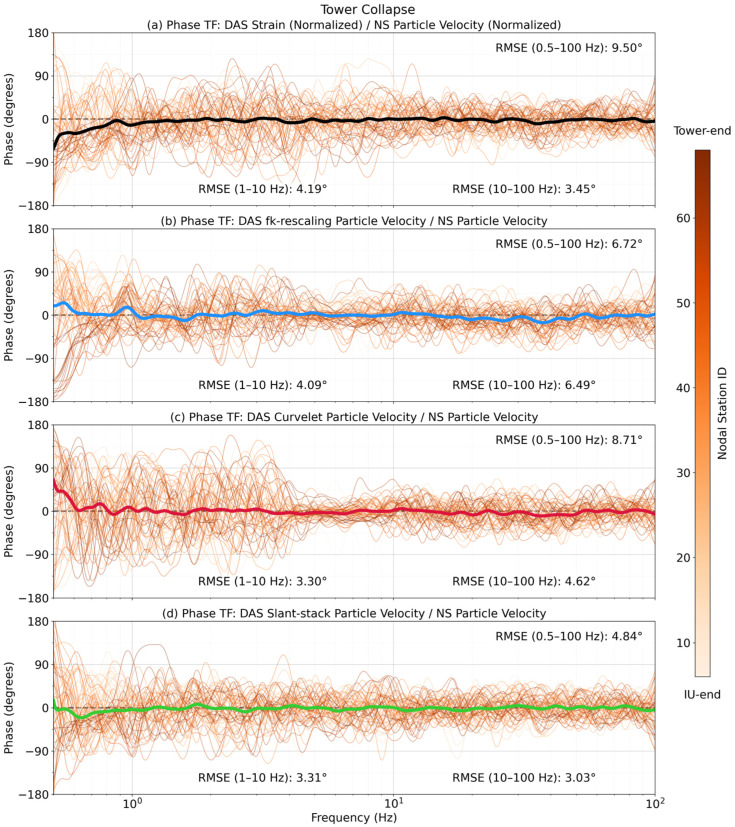
Phase TFs for all 63 DAS–NS pairs and the median TF for each strain-to-velocity conversion method for waveforms recorded during the tower collapse. Individual TF curves are color-coded by NS ID, varying from light orange at the IU-end to dark orange at the tower-end. Median TF curves calculated across all pairs are shown for (**a**) normalized waveforms, (**b**) fk-rescaling, (**c**) curvelet, and (**d**) slant-stack. RMSE values for the median TF are shown in each subplot for three frequency bands, 0.5–100 Hz, 1–10 Hz, and 10–100 Hz, using the phase RMSE metric.

Plots similar to Figure 3, Figure 4 and Figure 5 were generated for each of the 63 usable DAS–NS pairs using waveforms recorded during the tower collapse. To visualize the consistency across the 1.4 km array and reveal trends not apparent from a single pair, Figure 6 and Figure 7 extend the TF comparisons to all 63 DAS–NS pairs. Thin lines represent TFs from individual pairs, while bold lines show the overall median TFs. The RMSE values provided in the subplots are those calculated from the median TFs. For the amplitude (Figure 6), the individual TFs for each conversion method remain relatively clustered, and the median TFs capture the overall trends of a better amplitude agreement over the 1–10 Hz frequency band and a poorer amplitude agreement over the 10–100 Hz frequency band. The fk-rescaling method yields the lowest median RMSE_amp_ value of 0.56 across the entire frequency band from 0.5 to 100 Hz. It is also superior to all other methods in the individual frequency bands from 1 to 10 Hz and 10 to 100 Hz. The curvelet method has the highest RMSE_amp_ values in both the 1–10 Hz and 10–100 Hz frequency bands. The slant-stack method performs well in the lower frequency band, but poorly in the higher frequency band.

For the phase (Figure 7), the TFs calculated from the individual DAS–NS pairs are substantially more variable as a function of frequency, irrespective of the conversion method used. Thus, the median TFs simply average out the opposing phase fluctuations across individual pairs, producing median phase values that are generally less than 5°, depending on the frequency bandwidth and conversion method. These median phase differences, that tend to average out across numerous DAS–NS pairs, are likely a beneficial characteristic for DAS applications like surface wave testing, where large numbers of channels are simultaneously utilized in the wavefield transformations to calculate phase velocities. This is probably why researchers have reported a strong agreement between surface wave phase velocities calculated from traditional geophones and DAS [30]. However, the phase agreement between any given DAS–NS pair could vary substantially across frequencies, and the median phase TFs do not reflect the expected phase differences for any given pair. Hence, the results obtained from the median phase TFs need to be applied within context, and under consideration of the behavior for the individual DAS–NS pairs.

**Figure 8:**
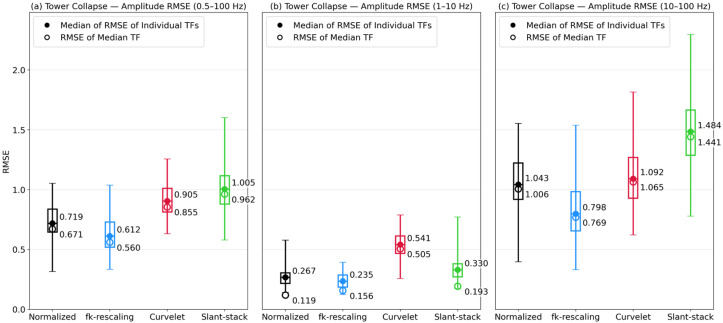
Box-and-whisker plots of amplitude TF RMSE values across all 63 DAS–NS pairs compared to the amplitude RMSE value of the median TF for each strain-to-velocity conversion method based on waveforms recorded during the tower collapse. Subplots for three frequency bands are provided: (**a**) 0.5–100 Hz, (**b**) 1–10 Hz, and (**c**) 10–100 Hz. Four conversion methods are shown in each subplot: normalized (black), fk-rescaling (blue), curvelet (red), and slant-stack (green). Filled circles indicate the median RMSE amplitude values of individual DAS–NS TFs, while open circles indicate the RMSE amplitude computed from the median TF.

**Figure 9:**
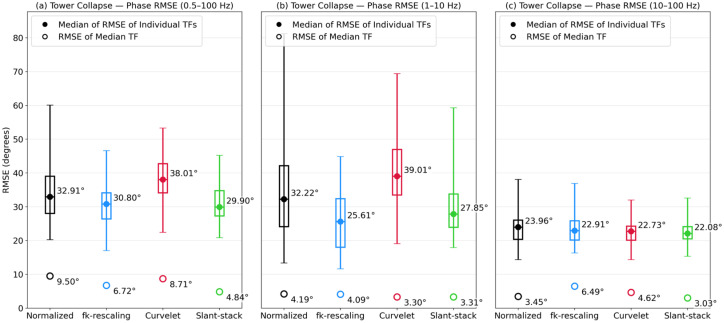
Box-and-whisker plots of phase TF RMSE values across all 63 DAS–NS pairs compared to the phase RMSE value of the median TF for each strain-to-velocity conversion method based on waveforms recorded during the tower collapse. Subplots for three frequency bands are provided: (**a**) 0.5–100 Hz, (**b**) 1–10 Hz, and (**c**) 10–100 Hz. Four conversion methods are shown in each subplot: normalized (black), fk-rescaling (blue), curvelet (red), and slant-stack (green). Filled circles indicate the median RMSE phase values of individual DAS–NS TFs, while open circles indicate the RMSE phase computed from the median TF.

To help synthesize and compare the RMSE values from the individual DAS–NS pair TFs with those from the median TFs, box-and-whisker plots of RMSE_amp_ and RMSE_phase_ are shown in Figure 8 and Figure 9, respectively. Filled circles indicate the median RMSE of the individual DAS–NS pairs, while open circles indicate the RMSE computed from the median TFs provided in Figure 6 and Figure 7. If the median TF is representative of the individual TFs, these values will be close to one another. The ends of each box represent the 25th- and 75th-percentile RMSE values calculated from the individual TFs, while the ends of the whiskers represent the minimum and maximum RMSE values from the individual TFs. A subplot is provided for each of the three different frequency bandwidths. In general, the amplitude RMSE values from the median TFs are similar to the median RMSE values from the individual pairs, and well within the statistic bounds of the box-and-whisker plots. However, the phase RMSE values from the median TFs are well outside of the statistical bounds of the box-and-whisker plots representing the individual phase TFs.

For the amplitude (Figure 8), the fk-rescaling method consistently yields the lowest median RMSE_amp_ and the least amount of scatter across all three frequency bands. The second best performing “method” is normalization (i.e., when the DAS strain amplitudes and NS velocity amplitudes are normalized by their respective maximum values). However, this approach does not allow for the conversion of the DAS strain to particle velocity. Rather, it simply shows that the relative amplitudes across the waveforms are similar to one another, and that strain-to-velocity conversion methods that result in larger RMSE_amp_ values than this normalized reference do, in fact, distort rather than improve the amplitude agreement post-conversion. The RMSE_amp_ values are notably best in the 1–10 Hz band and significantly higher and with greater scatter in the 10–100 Hz band. Based on the best performing fk-rescaling method, one could expect a median RMSE_amp_ value of 0.235 (i.e., an amplitude ratio of 10^0.235^ = 1.72) in the 1–10 Hz bandwidth, and a median RMSE_amp_ value of 0.798 (i.e., an amplitude ratio of 10^0.798^ = 6.28) in the 10–100 Hz bandwidth.

For the phase (Figure 9), the fk-rescaling and slant-stack methods generally outperform the curvelet method when the entire bandwidth from 0.5 to 100 Hz is considered. However, the results from all methods are better and much more similar in the 10–100 Hz bandwidth, with median RMSE_phase_ values calculated from the individual TFs of about 22–23°. In the 1–10 Hz band, the fk-rescaling method has the lowest median RMSE_phase_ value at 25.61°, while the curvelet method has the highest values at 39.01°.

While a direct one-to-one comparison between the amplitude misfit and phase misfit is not possible because they are quantified using different metrics (RMSE_amp_ in logarithmic ratio and RMSE_phase_ in degrees), the waterfall-style waveform comparison plots shown in the Electronic Supplement suggest that DAS measurements visually reproduce the phase characteristics more reliably than amplitude characteristics. This observation aligns with that made by Muir and Zhan in their 2022 paper [19], where they reported that DAS commonly preserves phase information more robustly than amplitude information.

### 5.2. Sledgehammer Source

**Figure 10:**
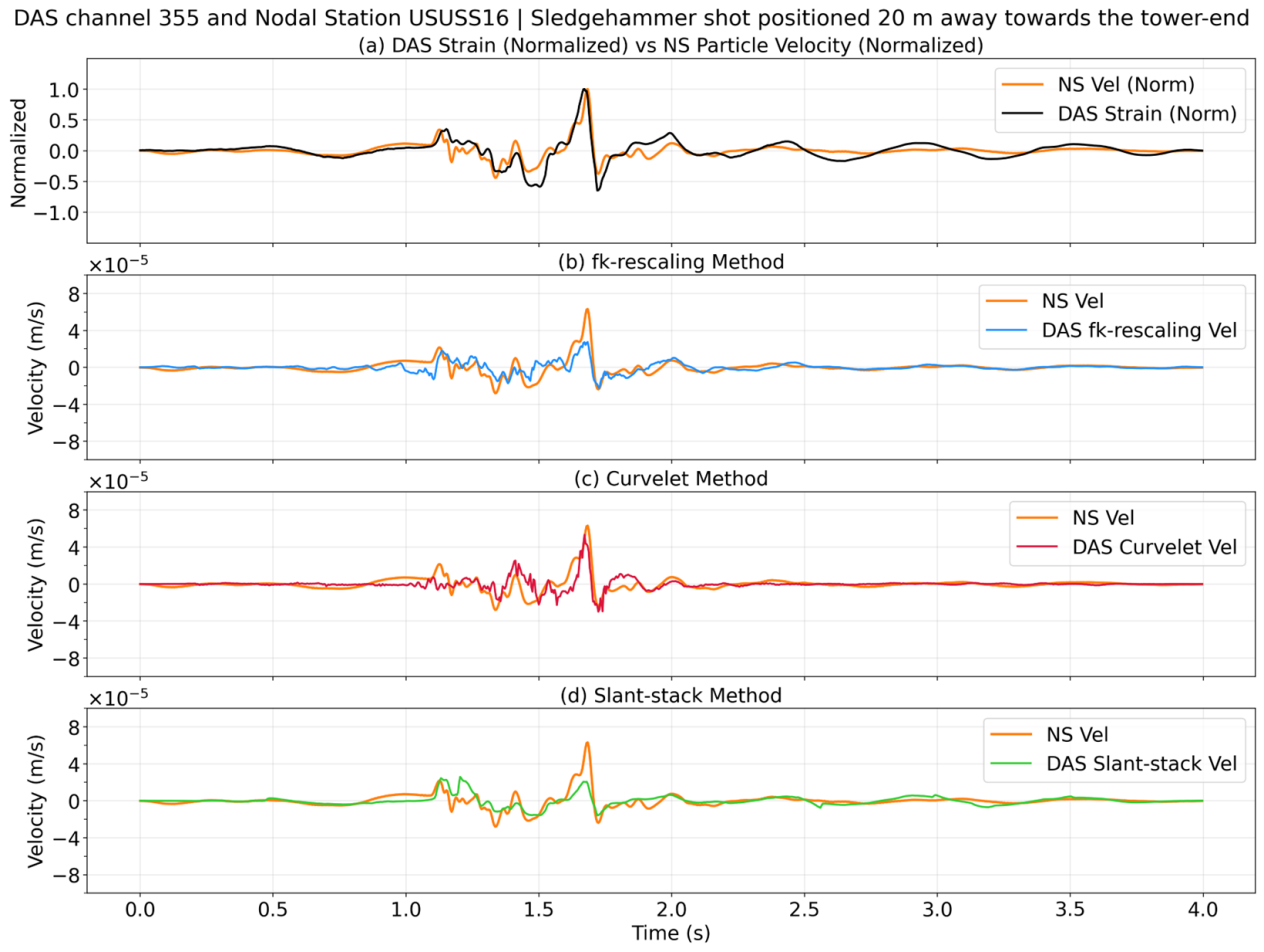
Time-domain waveforms recorded during the sledgehammer shot positioned 20 m away towards the tower-end of a DAS–NS pair (DAS channel 355 and Nodal Station USUSS16). Provided are (**a**) the NS particle velocity time history normalized by its peak particle velocity and the DAS strain time history normalized by its peak strain value, and (**b**–**d**) NS particle velocity time history versus DAS particle velocity time history converted from strain using three methods: (**b**) fk-rescaling, (**c**) curvelet, and (**d**) slant-stack.

Figure 10 presents an example of waveform comparisons for a sledgehammer shot positioned 20 m away (towards the tower-end) from an example DAS–NS pair: DAS Channel 355 and Nodal Station USUSS16. In Figure 10a, the DAS strain waveform and the NS particle velocity waveform, both normalized by their respective peak amplitudes, are shown to allow a visual comparison of waveform shapes in terms of the relative amplitude and relative timing (i.e., phase) prior to any strain-to-velocity conversion. Similar to Figure 3a for the tower collapse source, the normalized waveforms visually agree quite well with one another, indicating that DAS strain measurements preserve key phase characteristics before the conversion to particle velocity regardless of the source type. However, because the sledgehammer source generates a shorter-duration signal than the tower collapse source, the zoomed-in time scale makes differences in the waveforms more visually apparent. Similar to observations made with regard to the tower collapse waveforms shown in Figure 3a, the DAS sledgehammer waveform also seems to lack some of the higher frequency signals that are present in the NS waveform. The Electronic Supplement provides amplitude-normalized waterfall-style waveform comparison plots for sledgehammer shots positioned 20 m away from 63 DAS–NS pairs (Figures S14–S16).

The remaining panels in Figure 10 compare the sledgehammer shot waveforms for this DAS–NS pair in terms of particle velocity, with the DAS strain-to-velocity converted waveforms from the fk-rescaling, curvelet-based conversion, and slant-stack methods shown in Figure 10b–d, respectively. Visually, all three converted DAS waveforms appear to show a slightly worse agreement with the NS waveform than the normalized case. Out of the three conversion methods, the slant-stack method appears to perform the best overall, although it is hard to judge. Both the fk-rescaling method and the curvelet method introduce more high-frequency signals in the waveforms, with the effect more visible in the curvelet method waveform. The Electronic Supplement provides waterfall-style waveform comparison plots for sledgehammer shots positioned 20 m away from 63 DAS–NS pairs and for all three strain-to-velocity conversion methods (Figures S17–S25). Notably, some of the DAS particle velocity waveforms converted using the slant-stack method contain high-frequency spikes outside (either before or after) of the main sledgehammer signal (refer to Figures S23–S25). This behavior is likely related to an assumption of the slant-stack method that, at each time, the wavefield is dominated by a single coherent plane wave. Around the main arrivals, this assumption is more valid, and the converted DAS velocity generally matches the nodal waveform closely. Before and after the main arrivals, however, the signal is weaker and more dominated by noise, scattered waves, or interfering wavefields. In these regions, the estimated slowness can become unstable or change abruptly, producing artificial high-frequency spikes in the converted traces. This effect is likely amplified by the short local aperture (*L*_*stack* = 10) used for the sledgehammer processing, which improves the spatial locality but reduces the slowness stability.

**Figure 11:**
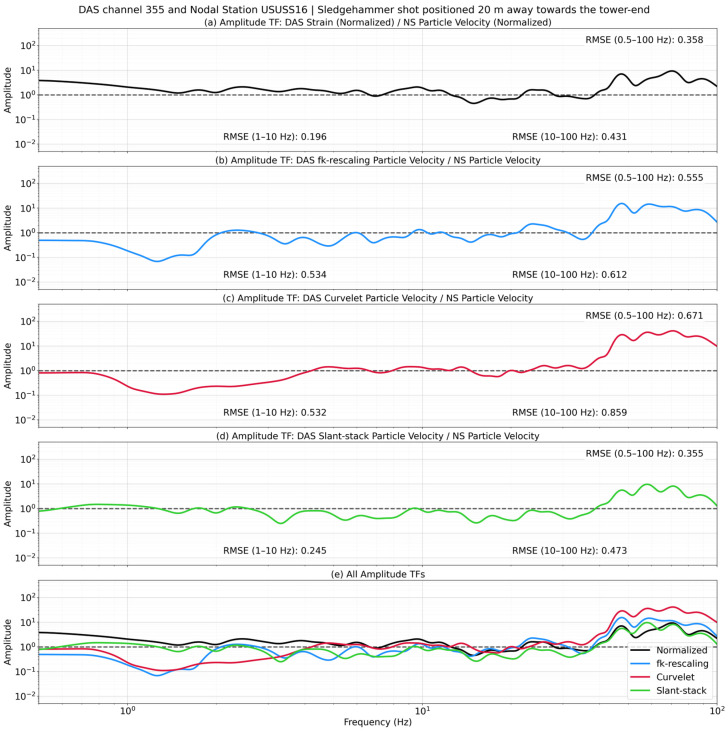
Amplitude TFs calculated for waveforms recorded during the sledgehammer shot positioned 20 m away towards the tower-end of a DAS–NS pair (DAS channel 355 and Nodal Station USUSS16). Provided are (**a**) normalized DAS strain to normalized NS particle velocity TF, (**b**) fk-rescaling DAS particle velocity to NS particle velocity TF, (**c**) curvelet DAS particle velocity to NS particle velocity TF, (**d**) slant-stack DAS particle velocity to NS particle velocity, and (**e**) all amplitude TFs overlaid. RMSE values are reported for three frequency bands, 0.5–100 Hz, 1–10 Hz, and 10–100 Hz, using the log-amplitude RMSE metric.

**Figure 12:**
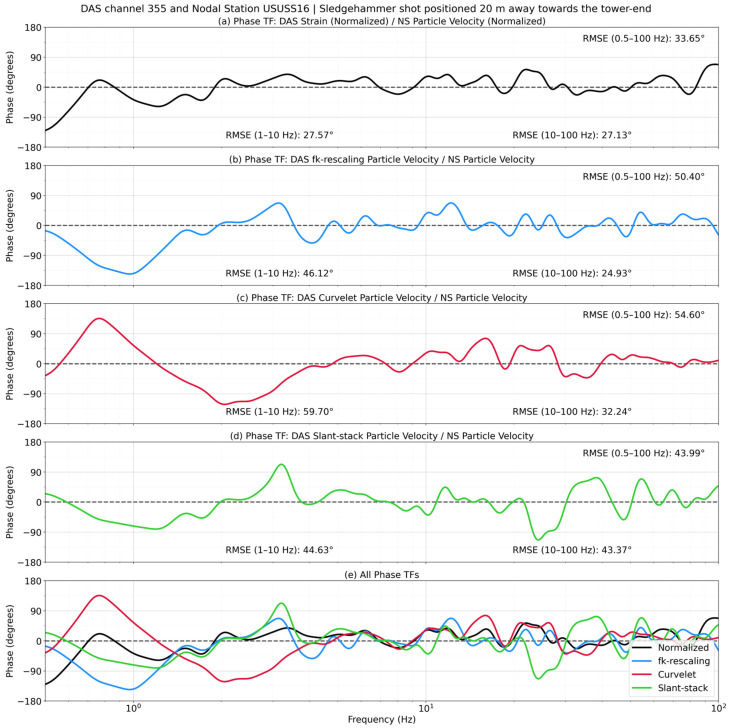
Phase TFs calculated for waveforms recorded during the sledgehammer shot positioned 20 m away towards the tower-end of a DAS–NS pair (DAS channel 355 and Nodal Station USUSS16). Provided are (**a**) normalized DAS strain to normalized NS particle velocity TF, (**b**) fk-rescaling DAS particle velocity to NS particle velocity TF, (**c**) curvelet DAS particle velocity to NS particle velocity TF, (**d**) slant-stack DAS particle velocity to NS particle velocity, and (**e**) all phase TFs overlaid. RMSE values are reported for three frequency bands, 0.5–100 Hz, 1–10 Hz, and 10–100 Hz, using the phase RMSE metric.

To quantify the waveform agreement as a function of frequency, the amplitude and phase TFs were computed for each DAS–NS pair using the same procedure described previously for the tower collapse source. Figure 11 and Figure 12 show the amplitude and phase TFs, respectively, over the frequency range of 0.5 to 100 Hz for the same DAS–NS pair waveforms shown in Figure 10. Subplot (a) in Figure 11 and Figure 12 represents the TFs calculated between the normalized DAS strain and NS particle velocity waveforms, subplots (b)–(d) represent the TFs calculated between the strain-to-velocity converted DAS waveforms and the NS particle velocity waveforms for the three DAS strain-to-velocity conversion methods, and subplot (e) overlays all cases to facilitate a direct comparison. Similar to the tower collapse, it can be observed that the agreement in amplitude and phase differs noticeably as a function of frequency. Therefore, the same three frequency bands used to quantitively evaluate RMSE values for the tower collapse waveforms were selected for evaluating the sledgehammer waveforms: (1) 0.5–100 Hz (overall performance), (2) 1–10 Hz (lower-frequency band), and (3) 10–100 Hz (higher-frequency band). The RMSE values for each frequency band and strain-to-velocity conversion method are reported within the corresponding subplots.

In Figure 11, the slant-stack method produced the lowest RMSE_amp_ values among the three conversion methods at each frequency band, and most closely approached the normalized DAS strain reference case. This method yielded an RMSE_amp_ value of 0.355 across the overall frequency bandwidth (equivalent to an average TF amplitude ratio of 10^0.355^ = 2.26), which is far superior to the best-performing fk-rescaling method for the example tower collapse amplitude TFs shown in Figure 4 that had an overall frequency bandwidth RMSE_amp_ = 0.712. Both the fk-rescaling method and the curvelet method perform more poorly across all three frequency bands. Notably, when compared to the example tower collapse amplitude TFs shown in Figure 4, the sledgehammer amplitude TFs indicate a better agreement at frequencies up to ~40 Hz. As observed in Figure 12, none of the three conversion methods outperformed the normalized raw strain case in terms of the RMSE_phase_ values across the entire 0.5–100 Hz bandwidth. However, similar to the example tower collapse phase TFs shown in Figure 5, the sledgehammer phase TFs indicate a better phase alignment at higher frequencies than lower frequencies. The fk-rescaling method performs the best overall in the 10–100 Hz band, with the lowest RMSE_phase_ value of 24.93°. Across the entire bandwidth, the curvelet method has the highest RMSE_phase_ value of 54.60°.

**Figure 13:**
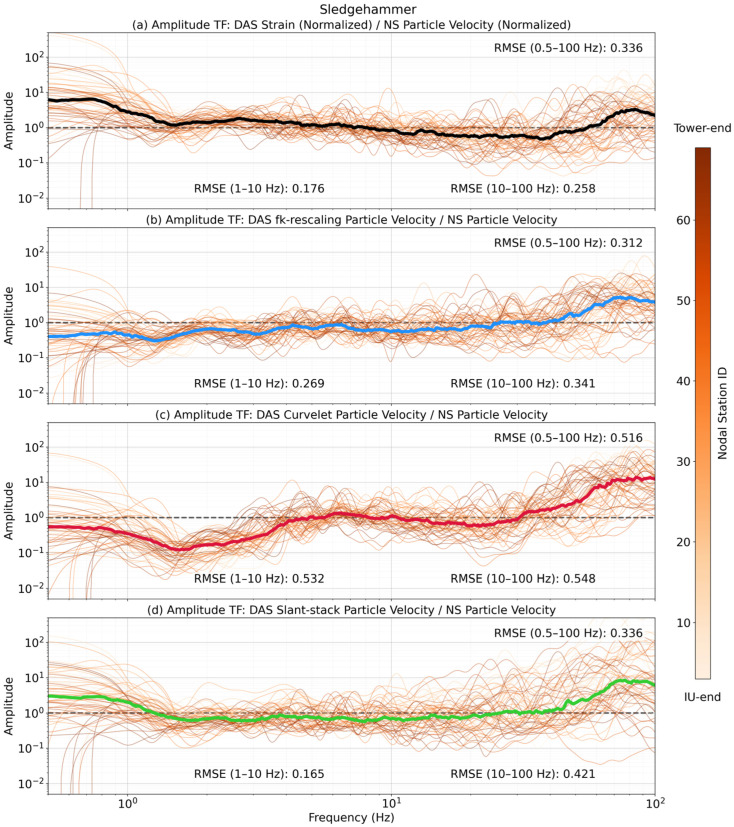
Amplitude TFs for all 63 DAS–NS pairs and the median TF for each strain-to-velocity conversion method for waveforms recorded during the sledgehammer shots. Individual TF curves are color-coded by NS ID, varying from light orange at the IU-end to dark orange at the tower-end. Median TF curves calculated across all pairs are shown for (**a**) normalized waveforms, (**b**) fk-rescaling, (**c**) curvelet, and (**d**) slant-stack. RMSE values for the median TF are shown in each subplot for three frequency bands, 0.5–100 Hz, 1–10 Hz, and 10–100 Hz, using the log-amplitude RMSE metric.

**Figure 14:**
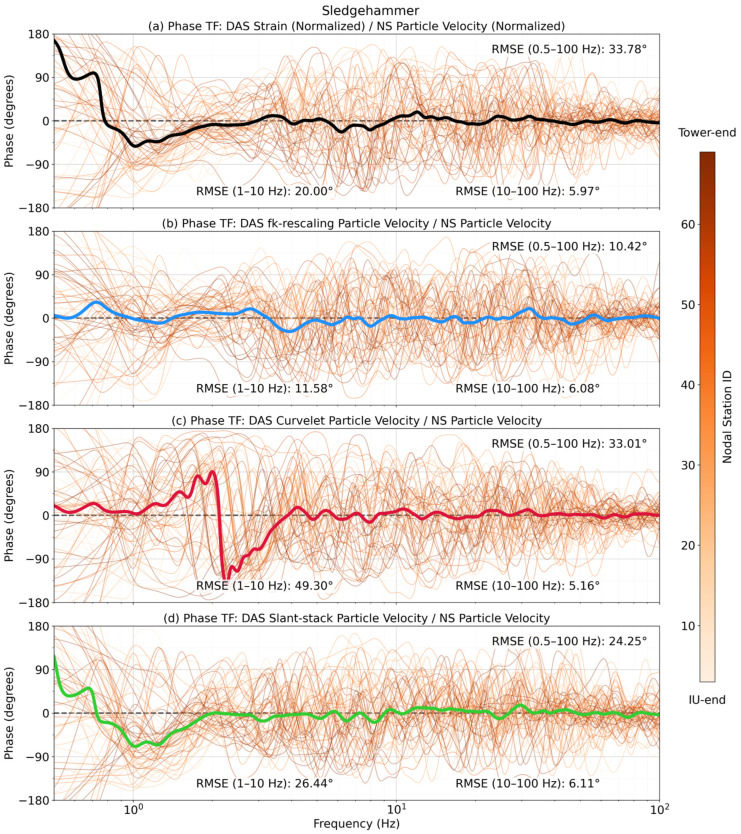
Phase TFs for all 63 DAS–NS pairs and the median TF for each strain-to-velocity conversion method for waveforms recorded during the sledgehammer shots. Individual TF curves are color-coded by NS ID, varying from light orange at the IU-end to dark orange at the tower-end. Median TF curves calculated across all pairs are shown for (**a**) normalized waveforms, (**b**) fk-rescaling, (**c**) curvelet, and (**d**) slant-stack. RMSE values for the median TF are shown in each subplot for three frequency bands, 0.5–100 Hz, 1–10 Hz, and 10–100 Hz, using the phase RMSE metric.

Plots similar to Figure 10, Figure 11 and Figure 12 were generated for each of the 63 usable DAS–NS pairs using waveforms recorded during the sledgehammer shots positioned 20 m away towards the tower-end of each pair. To visualize the consistency across the 1.4 km array and reveal trends not apparent from a single pair, Figure 13 and Figure 14 extend the TF comparisons to all 63 DAS–NS pairs. Thin lines represent TFs from individual pairs, while bold lines show the overall median TFs. The RMSE values provided in the subplots are those calculated from the median TFs. For the amplitude (Figure 13), the individual TFs for each conversion method remain relatively clustered, and the median TFs capture the overall trends of a better amplitude agreement over the 1–10 Hz frequency band and a poorer amplitude agreement over the 10–100 Hz frequency band, although the amplitude TFs actually show a pretty good agreement up to ~40 Hz. The fk-rescaling method yields the lowest median RMSE_amp_ value of 0.312 (i.e., an amplitude ratio of 10^0.312^ = 2.05) across the entire frequency band from 0.5 to 100 Hz. The curvelet method has the highest RMSE_amp_ values in both the 1–10 Hz and 10–100 Hz frequency bands. The slant-stack method has the best performance in the 1–10 Hz band with RMSE_amp_ value of 0.165. For the phase (Figure 14), the TFs calculated from the individual DAS–NS pairs are substantially more variable as a function of frequency, irrespective of the conversion method used. Similar to the tower collapse phase TFs shown in Figure 7, the median sledgehammer phase TFs average out the opposing phase fluctuations across individual pairs, producing median phase values that are much less than individual phase TF values, especially in the 10–100 Hz band.

**Figure 15:**
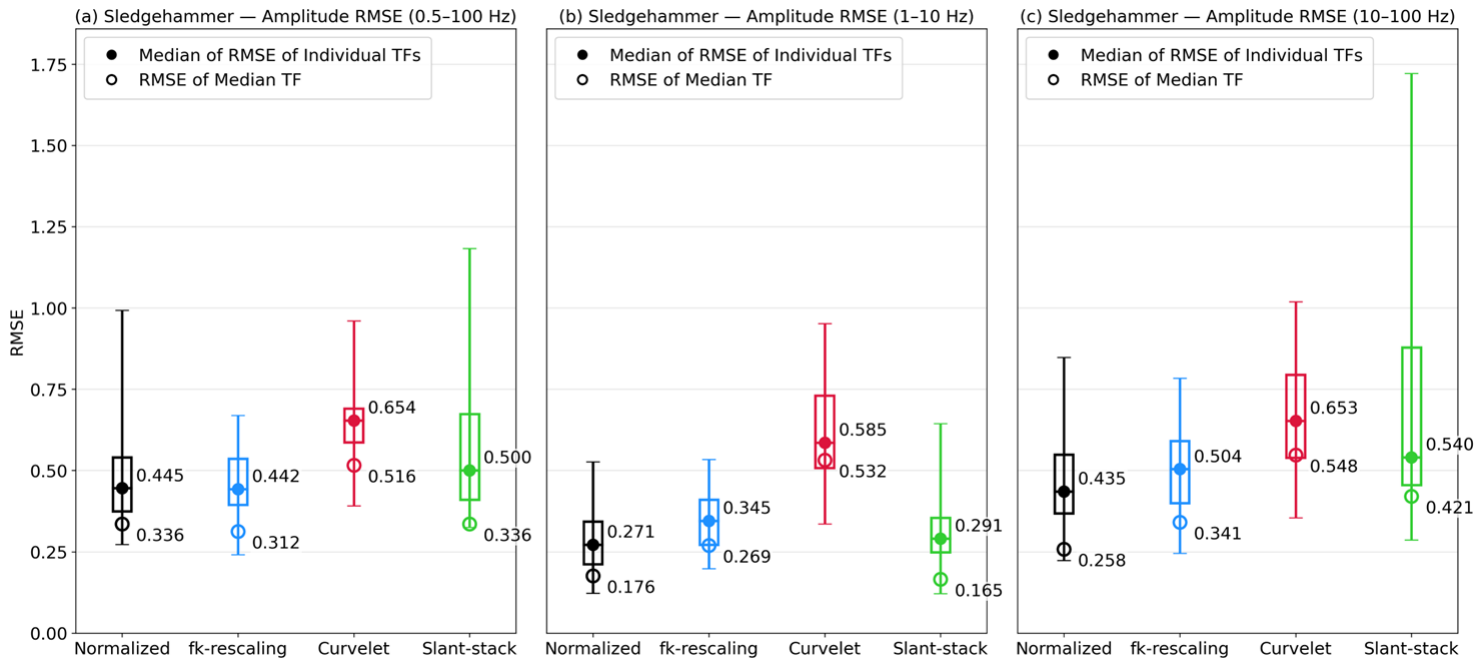
Box-and-whisker plots of amplitude TF RMSE values across all 63 DAS–NS pairs compared to the amplitude RMSE value of the median TF for each strain-to-velocity conversion method based on waveforms recorded during the sledgehammer shots. Subplots for three frequency bands are provided: (**a**) 0.5–100 Hz, (**b**) 1–10 Hz, and (**c**) 10–100 Hz. Four conversion methods are shown in each subplot: normalized (black), fk-rescaling (blue), curvelet (red), and slant-stack (green). Filled circles indicate the median RMSE amplitude values of individual DAS–NS TFs, while open circles indicate the RMSE amplitude computed from the median TF.

**Figure 16:**
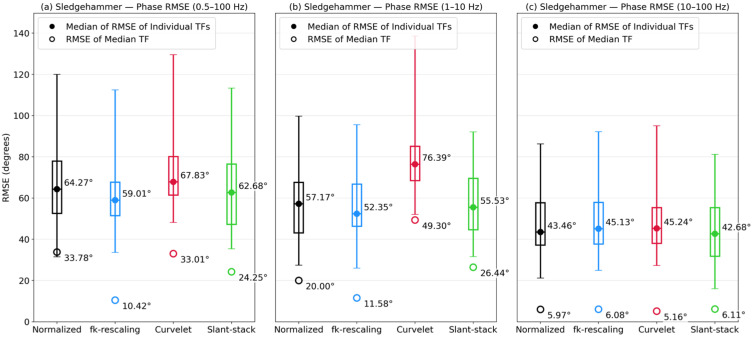
Box-and-whisker plots of phase TF RMSE values across all 63 DAS–NS pairs compared to the phase RMSE value of the median TF for each strain-to-velocity conversion method based on waveforms recorded during the sledgehammer shots. Subplots for three frequency bands are provided: (**a**) 0.5–100 Hz, (**b**) 1–10 Hz, and (**c**) 10–100 Hz. Four conversion methods are shown in each subplot: normalized (black), fk-rescaling (blue), curvelet (red), and slant-stack (green). Filled circles indicate the median RMSE phase values of individual DAS–NS TFs, while open circles indicate the RMSE phase computed from the median TF.

To help synthesize and compare the RMSE values from the individual DAS–NS pair TFs with those from the median TFs, box-and-whisker plots of RMSE_amp_ and RMSE_phase_ are shown in Figure 15 and Figure 16, respectively, for all three frequency bandwidths. In general, the amplitude RMSE values from the median TFs are similar to the median RMSE values from the individual pairs, and within the statistical bounds of the box-and-whisker plots. However, the phase RMSE values from the median TFs are well outside of the statistical bounds of the box-and-whisker plots representing the individual phase TFs. This, again, indicates that median phase TFs can provide an overly optimistic representation of phase agreement for any given DAS–NS pair.

For the amplitude (Figure 15), the fk-rescaling method tends to yield the lowest median RMSE_amp_ values with the least scatter across all bandwidths, although the slant-stack method also has comparably low values with a slightly higher scatter. For example, in the 1–10 Hz band, the slant-stack method has the lowest median RMSE_amp_ values. Overall, RMSE_amp_ values are notably best in the 1–10 Hz band and slightly higher and with a greater scatter in the 10–100 Hz band. Based on the overall best performing fk-rescaling method, one could expect a median RMSE_amp_ value of 0.345 (i.e., an amplitude ratio of 10^0.345^ = 2.21) in the 1–10 Hz bandwidth, and a median RMSE_amp_ value of 0.504 (i.e., an amplitude ratio of 10^0.504^ = 3.19) in the 10–100 Hz bandwidth.

For the phase (Figure 16), the fk-rescaling and slant-stack methods outperform the curvelet method when considering the performance across all three bandwidths. However, the results from all methods are better and much more similar in the 10–100 Hz bandwidth, with median RMSE_phase_ values calculated from the individual TFs of about 42–46°. In the 1–10 Hz band, the fk-rescaling method has the lowest median RMSE_phase_ value at 52.35°, while the curvelet method has the highest value at 76.39°.

Overall, the sledgehammer source confirms the same broad trends observed for the tower collapse source: fk-rescaling provides the best and most consistent overall amplitude and phase performance following DAS strain-to-velocity conversion, with the slant-stack method not far behind, while the curvelet method yields the poorest agreement in terms of both amplitude and phase.

### 5.3. Comparison Between Sources

**Figure 17:**
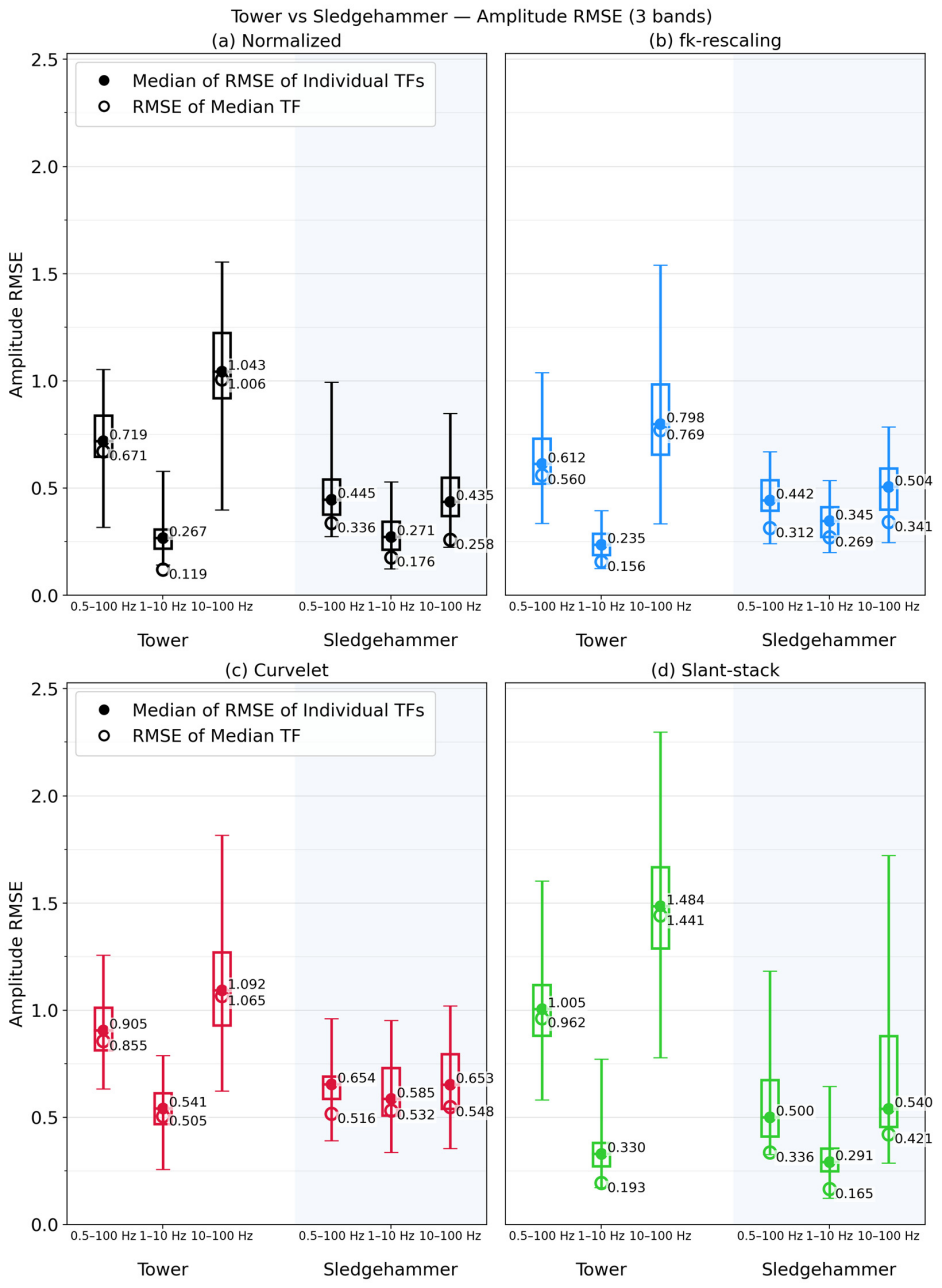
Box-and-whisker plots of amplitude TF RMSE values across all 63 DAS–NS pairs compared to the amplitude RMSE value of the median TF for each strain-to-velocity conversion method based on waveforms recorded during the tower collapse (on the left side of each subplot) and the sledgehammer shots (on the right side of each subplot with shaded background). Subplots for three frequency bands are provided: 0.5–100 Hz, 1–10 Hz, and 10–100 Hz. Four conversion methods are shown in each subplot: (**a**) normalized (black), (**b**) fk-rescaling (blue), (**c**) curvelet (red), and (**d**) slant-stack (green). Filled circles indicate the median RMSE amplitude values of individual DAS–NS TFs, while open circles indicate the RMSE amplitude computed from the median TF.

**Figure 18:**
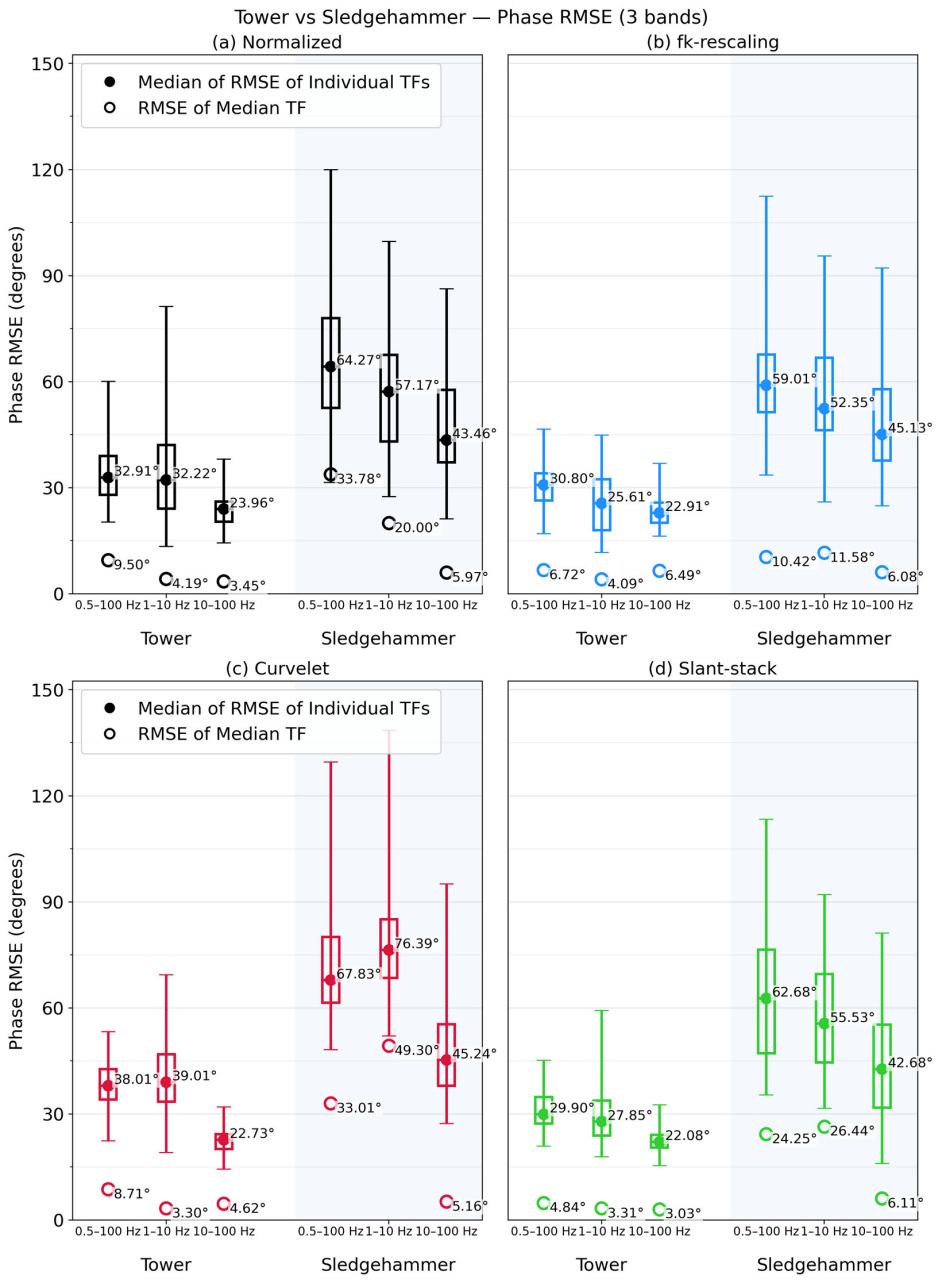
Box-and-whisker plots of phase TF RMSE values across all 63 DAS–NS pairs compared to the phase RMSE value of the median TF for each strain-to-velocity conversion method based on waveforms recorded during the tower collapse (on the left side of each subplot) and the sledgehammer shots (on the right side of each subplot with shaded background). Subplots for three frequency bands are provided: 0.5–100 Hz, 1–10 Hz, and 10–100 Hz. Four conversion methods are shown in each subplot: (**a**) normalized (black), (**b**) fk-rescaling (blue), (**c**) curvelet (red), and (**d**) slant-stack (green). Filled circles indicate the median RMSE phase values of individual DAS–NS TFs, while open circles indicate the RMSE phase computed from the median TF.

In order to compare the amplitude and phase reconstruction ability of each DAS strain-to-velocity conversion method across the two different sources used in this study (i.e., the tower collapse and the sledgehammer shots), box-and-whisker plots of the amplitude RMSE and phase RMSE for both sources are shown in Figure 17 and Figure 18, respectively. Each of these figures has four subplots, one for each conversion method. In each subplot, statistics across the three frequency bands of 0.5–100 Hz, 1–10 Hz, and 10–100 Hz are provided, with those for the tower collapse shown on the left and those for the sledgehammer shots shown on the right.

#### 5.3.1. Amplitude Comparisons

For the amplitude (Figure 17), RMSE values in the 1–10 Hz range are generally comparable between the two sources for each of the strain-to-velocity conversion methods. However, in the 10–100 Hz band, the amplitude RMSE values are significantly lower for the sledgehammer source than for the tower collapse source. In an attempt to better understand the potential causes for this, we investigated whether the poorer amplitude agreement in the 10–100 Hz band for the tower collapse source was related to the reduced high-frequency signal content on either the DAS or NS waveforms. To check this, signal-to-noise ratios (SNRs) were calculated for the tower collapse waveforms recorded by both sensing systems (DAS and NS) over the entire 0.5–100 Hz bandwidth (refer to Figure S26 in the Electronic Supplement). The exact same window lengths (i.e., 60 s) were used for both the signal and noise waveforms, and the median SNR across all 63 DAS and NS channels was calculated. For the tower source, the median SNRs for the DAS and NS are quite similar, with the NS values being slightly higher. The SNRs drop significantly for both sensing systems at frequencies greater than approximately 10 Hz and taper to an amplitude ratio of ~1.5 at 100 Hz. Comparatively, the SNRs for the sledgehammer source are quite a bit higher for both the DAS and NS waveforms over this 10–100 Hz bandwidth. Therefore, it is possible that the lower SNRs at high frequencies for the tower collapse waveforms may have contributed to the poorer amplitude agreement between the DAS and NS in the 10–100 Hz bandwidth for the tower source. However, we are skeptical that the lower SNRs for the tower collapse source over the 10–100 Hz bandwidth are responsible for the poorer high-frequency amplitude agreement between the DAS and NS pairs for the following reasons: (1) It is important to realize that the amplitude comparisons between the DAS and NS signals are completely relative to one another, regardless of whether the amplitudes were caused by “signal” or “noise”. (2) The median SNRs still remain greater than ~1.5 for the tower collapse waveforms over the entire 10–100 Hz bandwidth, and the SNRs for the sledgehammer waveforms recorded by the NS are not much higher than this over the 60–100 Hz bandwidth. (3) The phase RMSE values for the tower collapse waveforms over the 10–100 Hz bandwidth are consistently the lowest (refer to Figure 18), so one cannot blame low high-frequency SNRs in the tower collapse waveforms for the poor amplitude agreement when the phase agreement is remarkably the best for these same waveforms and bandwidth. The phase RMSE values are discussed in greater detail below.

#### 5.3.2. Phase Comparisons

For the phase (Figure 18), the RMSE values are consistently higher for the sledgehammer source across all frequency bands compared with the tower collapse source. The stronger phase agreement observed for the tower collapse source may reflect the differences in wavefield coherence between the two sources. DAS measurements are known to be sensitive to incoherent and scattered wavefields. For example, van den Ende and Ampuero [24] showed that raw DAS strain-rate data can exhibit a reduced waveform coherence, while the conversion to particle velocity via spatial integration improves the array processing performance. In this context, the longer-duration and higher-energy tower collapse source may have produced a more coherent wavefield than the localized sledgehammer impacts, contributing to an improved phase agreement for the tower collapse waveforms. As noted above, a general assumption would be to predict that the DAS–NS phase angles would be in better agreement at frequencies where the SNR is higher. However, contrary to this assumption, the RMSE_phase_ values were consistently higher over the 1–10 Hz bandwidth than the 10–100 Hz bandwidth for both sources, despite having higher SNRs over the 1–10 Hz bandwidth for both sources (refer to Figure S26 in the Electronic Supplement). Moreover, although the DAS and NS sledgehammer waveforms clearly have higher SNRs than the tower collapse waveforms at most frequencies in the 10–100 Hz bandwidth, the sledgehammer waveforms consistently have higher RMSE_phase_ values than the tower collapse waveforms.

In summary, the DAS and NS sledgehammer waveforms generally have a better amplitude agreement (i.e., lower RMSE_amp_ values) than the tower collapse waveforms, with the exception of the 1–10 Hz frequency bandwidth, where they are quite similar. However, the DAS and NS tower collapse waveforms clearly have a better phase agreement (i.e., lower RMSE_phase_ values) than the sledgehammer waveforms across all frequency bands. Furthermore, while the amplitude RMSE values are clearly lower over the 1–10 Hz bandwidth than the 10–100 Hz bandwidth for both source types, the phase values are lower over the 10–100 Hz bandwidth for both source types.

It is important to recognize that DAS does not measure the strain at a point. Rather, each measurement represents the average strain over a finite gauge length. This spatial averaging acts as a form of low-pass spatial filter and limits the shortest wavelength that can be accurately resolved. Vantassel et al. [13] noted that the shortest wavelength that can be reliably resolved using DAS is equal to either two times the receiver (channel) spacing or the gauge length, whichever is larger. This consideration is relevant to the interpretation of the high-frequency amplitude discrepancies observed in this study, as shorter wavelengths (i.e., higher frequencies) are inherently more strongly affected by gauge-length averaging. Additionally, variations in the trenching depth and backfill compaction, local soil type and moisture content, and cable-ground coupling quality along the 1.4 km array were not directly quantified in this study, but likely contribute to the amplitude scatter between the DAS-derived and NS particle velocities, particularly at higher frequencies where DAS measurements are more sensitive to small-scale coupling heterogeneity.

### 5.4. Practical Implications on Strain-to-Velocity Parameter Selection

All quantitative evaluations of the DAS strain-to-velocity conversion in this study are based on comparisons with a collocated NS reference measurement. These collocated reference measurements were used in RMSE-based sensitivity studies to determine the optimal sets of parameters for each strain-to-velocity conversion method and source type (refer to Section 4 and Section 5.1). During these sensitivity studies, it was observed that the strain-to-velocity conversion results were highly dependent on the choice of input parameters. However, in many DAS deployments, reference measurements from NS or broadband sensors may not be available to help select the optimal input parameters. In the absence of collocated reference sensors, alternative approaches must be used for the strain-to-velocity parameter selection. Some approaches, such as a data-driven deep-learning framework capable of estimating the particle velocity directly from the DAS strain without explicit phase-velocity estimation [18], and efforts to generate the DAS instrument response directly without relying solely on collocated reference sensors [19], show promise for the strain-to-velocity conversion. However, to date, these studies have largely focused on earthquake waveform datasets, which differ from the high-energy, near-field, and higher-frequency active source datasets used in this study. Therefore, further research is needed in order to understand how variations in the source type, frequency content, strain intensity, site conditions, and acquisition geometry impact DAS strain-to-velocity conversions.

### 5.5. Computational Considerations

In addition to the conversion performance, computational efficiency is an important practical consideration. The curvelet-based method is significantly more memory-intensive and time-consuming compared to the fk-rescaling and slant-stack methods. In one comparative runtime study, the curvelet method required an approximately 25-times-longer processing time than the fk-rescaling and slant-stack methods for the same set of waveforms. Given that the fk-rescaling and slant-stack methods outperformed the curvelet method in this study, its use may not be justified for large active-source datasets or real-time applications. The fk-rescaling and slant-stack methods are computationally more efficient, while also providing a better performance, making them more suitable for practical implementation.

### 5.6. Overall Observations

The superior performance of the fk-rescaling method in this study likely results from its explicit use of frequency–wavenumber scaling, which is well-suited for coherent, predominantly one-dimensional wave propagation along the array during the active-source events. Lior et al. noted that fk-rescaling retains its robustness across complex wavefields because it accounts for varying apparent velocities throughout the full *f*–*k* domain [27]. More recently, Boullenger et al. demonstrated that fk-rescaling can recover calibrated particle-velocity records suitable for earthquake source characterization [30].

The competitive performance of the slant-stack method is consistent with the semblance-based local slowness framework proposed by Lior et al. [27], in which the apparent phase velocity is resolved as a function of time. This allows the separation of rapidly changing arrivals and scattered energy, making the method particularly suitable for impulsive and spatially variable sources such as sledgehammer impacts.

While the curvelet method underperformed for these active-source datasets, it may remain advantageous for more complex two-dimensional wavefields or curved fiber geometries where directional decomposition is beneficial. Atterholt et al. noted that curvelets efficiently represent curved wavefronts and discontinuities for geometrically complex wavefields [26]. Therefore, its poorer performance here may reflect the relatively simple linear-array geometry and uni-directional wave propagation patterns rather than a general limitation of the method.

We emphasize that the plane-wave and locally coherent-wavefield assumptions underlying all three conversion methods are more likely to hold for the tower collapse source, which generates a longer-duration, lower-frequency, and more spatially coherent wavefield, than for the sledgehammer source, which produces an impulsive, near-field excitation more prone to scattered arrivals, complex multimodal surface-wave propagation, and rapidly varying apparent velocities. We attempted to partially mitigate the near-field effects for the sledgehammer source by maintaining a minimum offset of 10 channels between the source and the nearest DAS channel within each processing window (Figure 2). However, this offset does not guarantee fully coherent, plane-wave behavior, and some of the high-frequency spikes observed for the sledgehammer source may be attributable to localized violations of these assumptions.

## 6. Conclusions

This study evaluated three widely used DAS strain-to-velocity conversion approaches: the fk-rescaling, curvelet-based conversion, and slant-stack methods. These approaches were applied to a unique high-energy, near-field, active-source dataset collected at the Birds Landing Site in Sherman Island, California. The dataset includes wavefields generated by a large transmission tower collapse and sledgehammer impacts used for subsurface imaging. The wavefields were recorded simultaneously by a 1.4 km DAS array and collocated nodal stations spaced every 20 m along the DAS array. Using 63 DAS–NS pairs, we have quantified the strain-to-velocity conversion method performance using amplitude and phase transfer functions (TFs) between DAS-derived and NS particle velocity records, with the root-mean-square error (RMSE) evaluated across three frequency bands: 0.5–100 Hz, 1–10 Hz, and 10–100 Hz.

The results show that the fk-rescaling method provides the most accurate amplitude response across both source types and all frequency bands, while both the fk-rescaling and slant-stack methods generally yield the best phase agreement. All methods yield a poorer amplitude reconstruction at frequencies higher than 10–40 Hz, while all methods yield a poorer phase reconstruction at frequencies less than 10 Hz. Based on a visual inspection of the waveform comparisons, the phase information generally appears to be reconstructed more reliably than amplitude information; however, because the amplitude RMSE and phase RMSE are expressed in different units, this observation cannot be quantitatively verified. Furthermore, we note that phase errors remain substantial in some cases, particularly for the sledgehammer source. Based on the average RMSE amplitude values and when using the best-performing fk-rescaling method, one could expect the converted DAS particle velocity amplitudes to be within a factor of 1.7 to 2.2 (i.e., log-amplitude RMSE values from 0.235 to 0.345) of the NS particle velocity amplitudes over the 1–10 Hz frequency range, and within a factor of 3.2 to 6.3 (i.e., log-amplitude RMSE values from 0.504 to 0.798) of the NS particle velocity amplitudes over the 10–100 Hz band. Based on the average RMSE phase values and when using the fk-rescaling method, one could expect the converted DAS particle velocity phase angles to vary from approximately 25 to 52° from the NS particle velocity phase angles over the 1–10 Hz frequency range, and from approximately 23 to 45° from the NS particle velocity phase angles over the 10–100 Hz frequency range.

These findings indicate that the fk-rescaling method is the most robust choice for active-source DAS applications, while the slant-stack method provides a strong alternative when the phase accuracy is prioritized. More broadly, this study demonstrates that conversion method selection and parameter tuning should consider the source characteristics, frequency range, computational cost, and array geometry.

## Figures and Tables

**Table 1:** Optimal set of parameters for each strain-to-velocity conversion method and source type.

Source Type	fk-Rescaling Method	Curvelet Method	Slant-Stack Method
Tower Collapse	*dx* = 1.017 m	*dx* = 1.017 m	*dx* = 1.017 m
	*f_s_* = 250 Hz	*f_s_* = 250 Hz	*f_s_* = 250 Hz
	*taper* = (0.02, 0.05)	*pad* = (0.25, 0.25)	*slm* = 0.005 s/m
	*f_max_* = 125 Hz	*scale*_*begin* = 2	*sls* = 0.0025 s/m
	*k_min_* = (0.0017, 0.002) m^−1^	*nbscales* = 6	*L*_*stack* = 50 channels
	*v_max_* = (750, 800) m/s	*nbangles* = 8	*frqlow* = 0.5 Hz
	*edge* = 0.2		*frqhigh* = 125 Hz
Sledgehammer (all values match the tower collapse source unless indicated otherwise)	*k_min_* = (0.020, 0.022) m^−1^	*nbscales* = 4	*slm* = 0.1 s/m
	*v_max_* = (250, 275) m/s		*sls* = 0.0002 s/m
			*L*_*stack* = 10 channels

## Data Availability

The data underlying this study are currently being organized into an open-access database that will be published soon on the DesignSafe data repository [33]. DesignSafe is a U.S. National Science Foundation-supported, open-access cyberinfrastructure platform developed to support natural hazards engineering research. It provides curated, citable data repositories with assigned DOIs and is widely used within the natural hazards and civil engineering communities. Upon publication, the database will be accessible through DesignSafe by searching the keywords “Birds Landing”, “DAS”, and/or “nodal stations”. In addition to raw data, trimmed data, and metadata, the published dataset will include example processing and visualization codes (e.g., for plotting waveforms). The strain-to-velocity conversion codes used in this study are part of the open-access DASPy toolbox [29], which is already publicly available. Readers who have difficulty locating the dataset are welcome to contact the corresponding author directly.

## Supplementary Materials

The following supporting information can be downloaded at: https://www.mdpi.com/article/10.3390/s26154673/s1, Figure S1: Dispersion data obtained from the DAS array using active and passive sources and used to inform the choice of $v_{max}$ and $k_{min}$ parameters in the fk-rescaling method, as explained in Section 4.1 in the paper; Figure S2: Waterfall-style comparison plots of normalized amplitude for DAS–NS pairs corresponding to nodal stations USUSS68–48 (starting from the pair closest to the tower-end) recorded during the tower collapse; Figure S3: Waterfall-style comparison plots of normalized amplitude for DAS–NS pairs corresponding to nodal stations USUSS47-27 recorded during the tower collapse; Figure S4: Waterfall-style comparison plots of normalized amplitude for DAS–NS pairs corresponding to nodal stations USUSS26-06 recorded during the tower collapse; Figure S5: Waterfall-style comparison plots of particle velocity for DAS–NS pairs corresponding to nodal stations USUSS68-48 (starting from the pair closest to the tower-end) recorded during the tower collapse based on the fk-rescaling method; Figure S6: Waterfall-style comparison plots of particle velocity for DAS–NS pairs corresponding to nodal stations USUSS47-27 recorded during the tower collapse based on the fk-rescaling method; Figure S7: Waterfall-style comparison plots of particle velocity for DAS–NS pairs corresponding to nodal stations USUSS26-06 recorded during the tower collapse based on the fk-rescaling method; Figure S8: Waterfall-style comparison plots of particle velocity for DAS–NS pairs corresponding to nodal stations USUSS68-48 (starting from the pair closest to the tower-end) recorded during the tower collapse based on the curvelet method; Figure S9: Waterfall-style comparison plots of particle velocity for DAS–NS pairs corresponding to nodal stations USUSS47-27 recorded during the tower collapse based on the curvelet method; Figure S10: Waterfall-style comparison plots of particle velocity for DAS–NS pairs corresponding to nodal stations USUSS26-06 recorded during the tower collapse based on the curvelet method; Figure S11: Waterfall-style comparison plots of particle velocity for DAS–NS pairs corresponding to nodal stations USUSS68-48 (starting from the pair closest to the tower-end) recorded during the tower collapse based on the slant-stack method; Figure S12: Waterfall-style comparison plots of particle velocity for DAS–NS pairs corresponding to nodal stations USUSS47-27 recorded during the tower collapse based on the slant-stack method; Figure S13: Waterfall-style comparison plots of particle velocity for DAS–NS pairs corresponding to nodal stations USUSS26-06 recorded during the tower collapse based on the slant-stack method; Figure S14: Waterfall-style comparison plots of normalized amplitude for DAS–NS pairs corresponding to nodal stations USUSS69-47 (starting from the pair closest to the tower-end) recorded during sledgehammer shots positioned 20 m away towards the tower-end of each pair; Figure S15: Waterfall-style comparison plots of normalized amplitude for DAS–NS pairs corresponding to nodal stations USUSS46-26 recorded during sledgehammer shots positioned 20 m away towards the tower-end of each pair; Figure S16: Waterfall-style comparison plots of normalized amplitude for DAS–NS pairs corresponding to nodal stations USUSS25-03 recorded during sledgehammer shots positioned 20 m away towards the tower-end of each pair; Figure S17: Waterfall-style comparison plots of particle velocity for DAS–NS pairs corresponding to nodal stations USUSS69-47 (starting from the pair closest to the tower-end) recorded during sledgehammer shots positioned 20 m away towards the tower-end of each pair based on the fk-rescaling method; Figure S18: Waterfall-style comparison plots of particle velocity for DAS–NS pairs corresponding to nodal stations USUSS46-26 recorded during sledgehammer shots positioned 20 m away towards the tower-end of each pair based on the fk-rescaling method; Figure S19: Waterfall-style comparison plots of particle velocity for DAS–NS pairs corresponding to nodal stations USUSS25-03 recorded during sledgehammer shots positioned 20 m away towards the tower-end of each pair based on the fk-rescaling method; Figure S20: Waterfall-style comparison plots of particle velocity for DAS–NS pairs corresponding to nodal stations USUSS69-47 (starting from the pair closest to the tower-end) recorded during sledgehammer shots positioned 20 m away towards the tower-end of each pair based on the curvelet method; Figure S21: Waterfall-style comparison plots of particle velocity for DAS–NS pairs corresponding to nodal stations USUSS46-26 recorded during sledgehammer shots positioned 20 m away towards the tower-end of each pair based on the curvelet method; Figure S22: Waterfall-style comparison plots of particle velocity for DAS–NS pairs corresponding to nodal stations USUSS25-03 recorded during sledgehammer shots positioned 20 m away towards the tower-end of each pair based on the curvelet method; Figure S23: Waterfall-style comparison plots of particle velocity for DAS–NS pairs corresponding to nodal stations USUSS69-47 (starting from the pair closest to the tower-end) recorded during sledgehammer shots positioned 20 m away towards the tower-end of each pair based on the slant-stack method; Figure S24: Waterfall-style comparison plots of particle velocity for DAS–NS pairs corresponding to nodal stations USUSS46-26 recorded during sledgehammer shots positioned 20 m away towards the tower-end of each pair based on the slant-stack method; Figure S25: Waterfall-style comparison plots of particle velocity for DAS–NS pairs corresponding to nodal stations USUSS25-03 recorded during sledgehammer shots positioned 20 m away towards the tower-end of each pair based on the slant-stack method; Figure S26: Comparison of median SNR plots for both DAS and NS waveforms over 0.5–100 Hz bandwidth for (a) the tower collapse source, and (b) the sledgehammer source. Note that the same window lengths were used for the signal and noise portions when calculating SNRs for the tower collapse waveforms (60 s) and the sledgehammer waveforms (4 s). Table S1: Range of values tested during the sensitivity study for the most influential parameters of each strain-to-velocity conversion method and source type, and the selected optimal value used for the final analysis presented in the main text in Table 1. Only the parameters identified in Section 4 as most influential for each method are listed; remaining parameters were held fixed at the values reported in Table 1. Note that for the fk-rescaling method, $k_{min}$ and $v_{max}$ are reported as tapered ranges (lower bound, upper bound), consistent with the format used in Table 1 of the main text.
