# Supplementary material for: Comparative Analysis of Strain-to-Velocity Conversion Methods for Active-Source DAS Data and Collocated Nodal Stations
Source: Sensors (Basel). 2026 Jul 23;26(15):4673. doi: 10.3390/s26154673 (PMC13468356; doi:10.3390/s26154673)
Supplement: Supplementary file 1 [file sensors-26-04673-s001.zip › sensors-4373346-supplementary.pdf]

Electronic Supplement

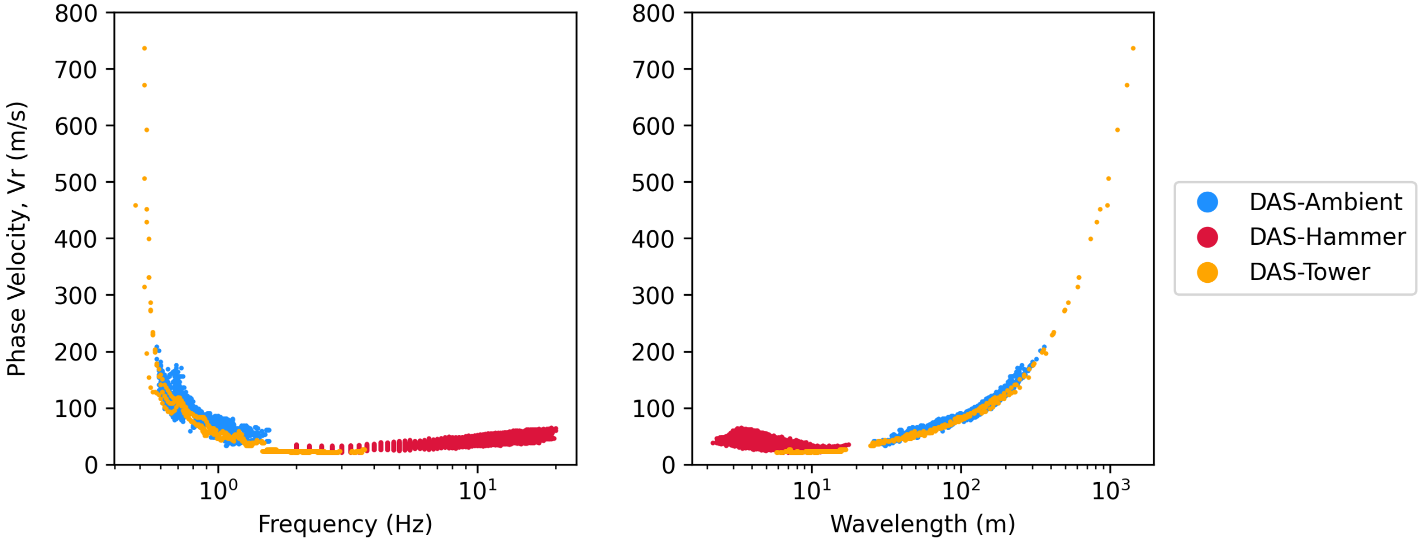

Figure S1: Dispersion data obtained from the DAS array using active and passive sources and used to inform the choice of v_{max} and k_{min} parameters in the fk-rescaling method, as explained in Section 4.1 in the paper.

Source Type	Method	Parameter	Minimum Value	Maximum Value	Optimal Value
Tower Collapse	fk-rescaling	k_{min}	(0.00133, 0.00167) m^{-1}	(0.0074, 0.0091) m^{-1}	(0.0017, 0.002) m^{-1}
		$v_{\max}$	(250, 500) m/s	(800, 1000) m/s	(750, 800) m/s
	Curvelet	$scale\_begin$	1	3	2
		$nbscales$	4	7	6
		$nbangles$	4	32	8
	Slant-stack	slm	0.003 s/m	0.008 s/m	0.005 s/m
		sls	0.0002 s/m	0.003 s/m	0.0025 s/m
Sledgehammer	fk-rescaling	k_{min}	(0.0143, 0.0154) m^{-1}	(0.0385, 0.0435) m^{-1}	(0.020, 0.022) m^{-1}
		$v_{\max}$	(90, 100) m/s	(275, 300) m/s	(250, 275) m/s
	Curvelet	$scale\_begin$	1	3	2
		$nbscales$	4	7	4
		$nbangles$	4	32	8
	Slant-stack	slm	0.025 s/m	0.1 s/m	0.1 s/m
		sls	0.0001 s/m	0.0005 s/m	0.0002 s/m

Table S1: Range of values tested during the sensitivity study for the most influential parameters of each strain-to-velocity conversion method and source type, and the selected optimal value used for the final analysis presented in the main text in Table 1. Only the parameters identified in Section 4 as most influential for each method are listed; remaining parameters were held fixed at the values reported in Table 1. Note that for the fk-rescaling method, k_{min} and $v_{\max}$ are reported as tapered ranges (lower bound, upper bound), consistent with the format used in Table 1 of the main text.

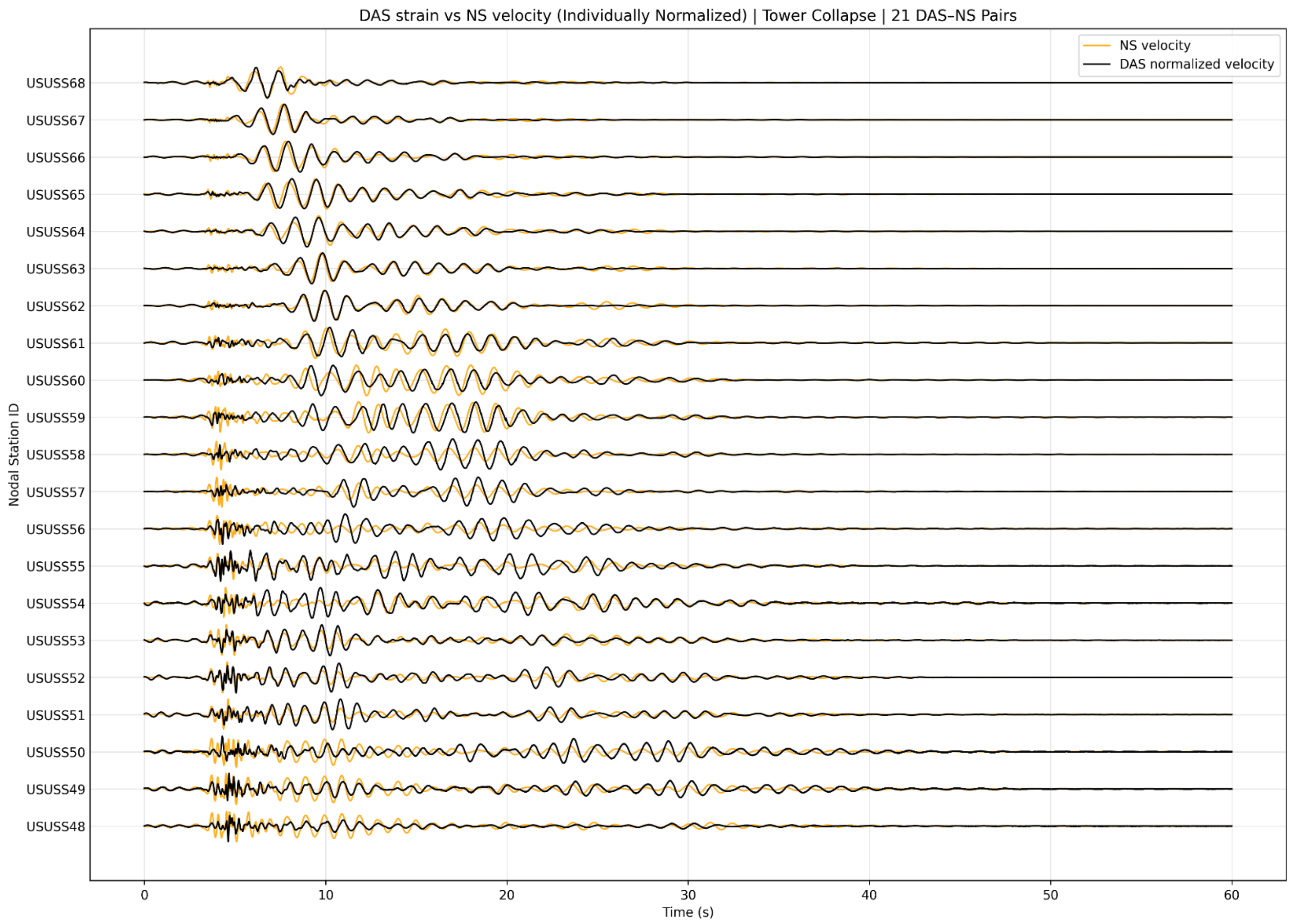

Figure S2: Waterfall-style comparison plots of normalized amplitude for DAS-NS pairs corresponding to nodal stations USUSS68-48 (starting from the pair closest to the tower-end) recorded during the tower collapse.

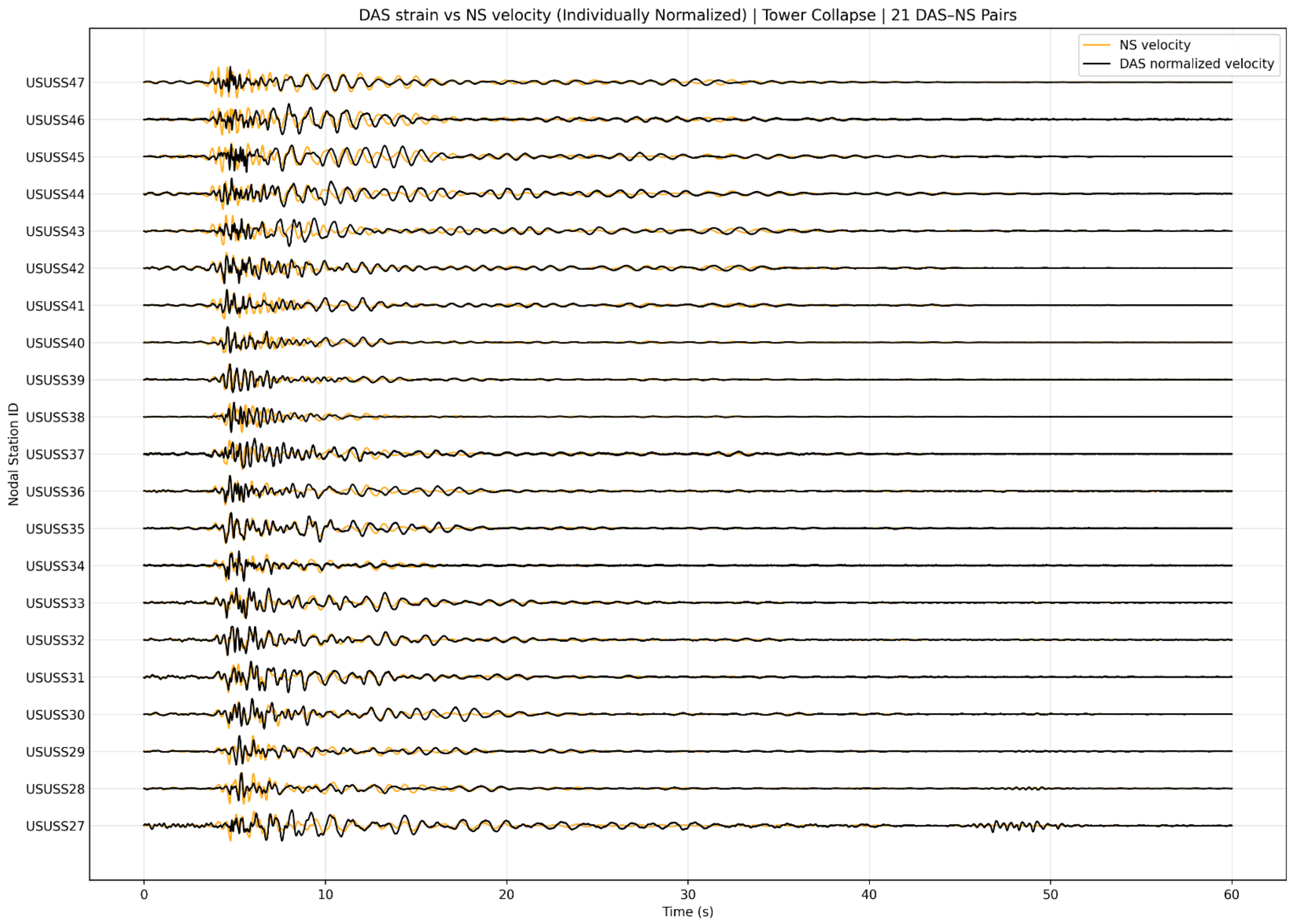

Figure S3: Waterfall-style comparison plots of normalized amplitude for DAS-NS pairs corresponding to nodal stations USUSS47-27 recorded during the tower collapse.

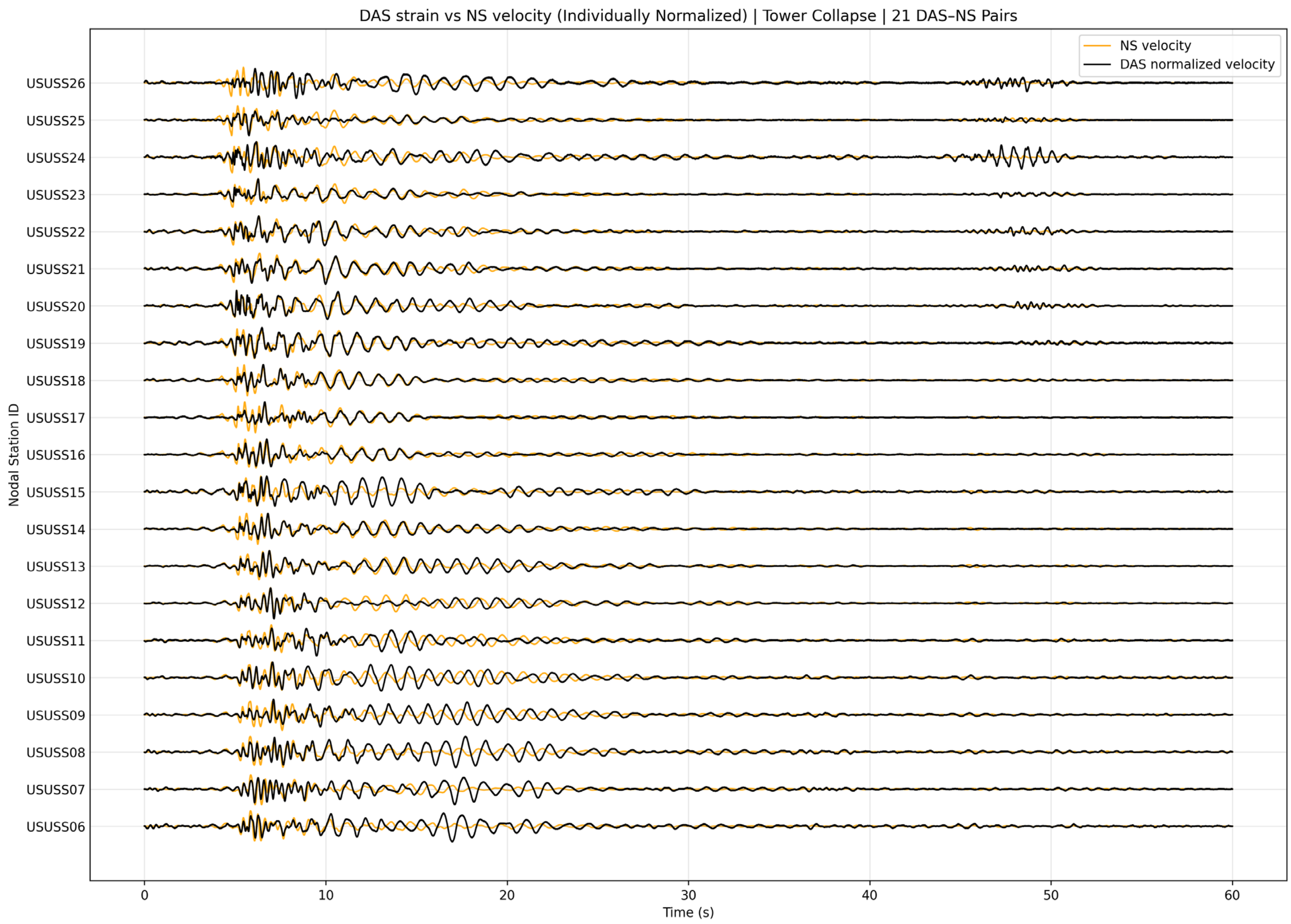

Figure S4: Waterfall-style comparison plots of normalized amplitude for DAS-NS pairs corresponding to nodal stations USUSS26-06 recorded during the tower collapse.

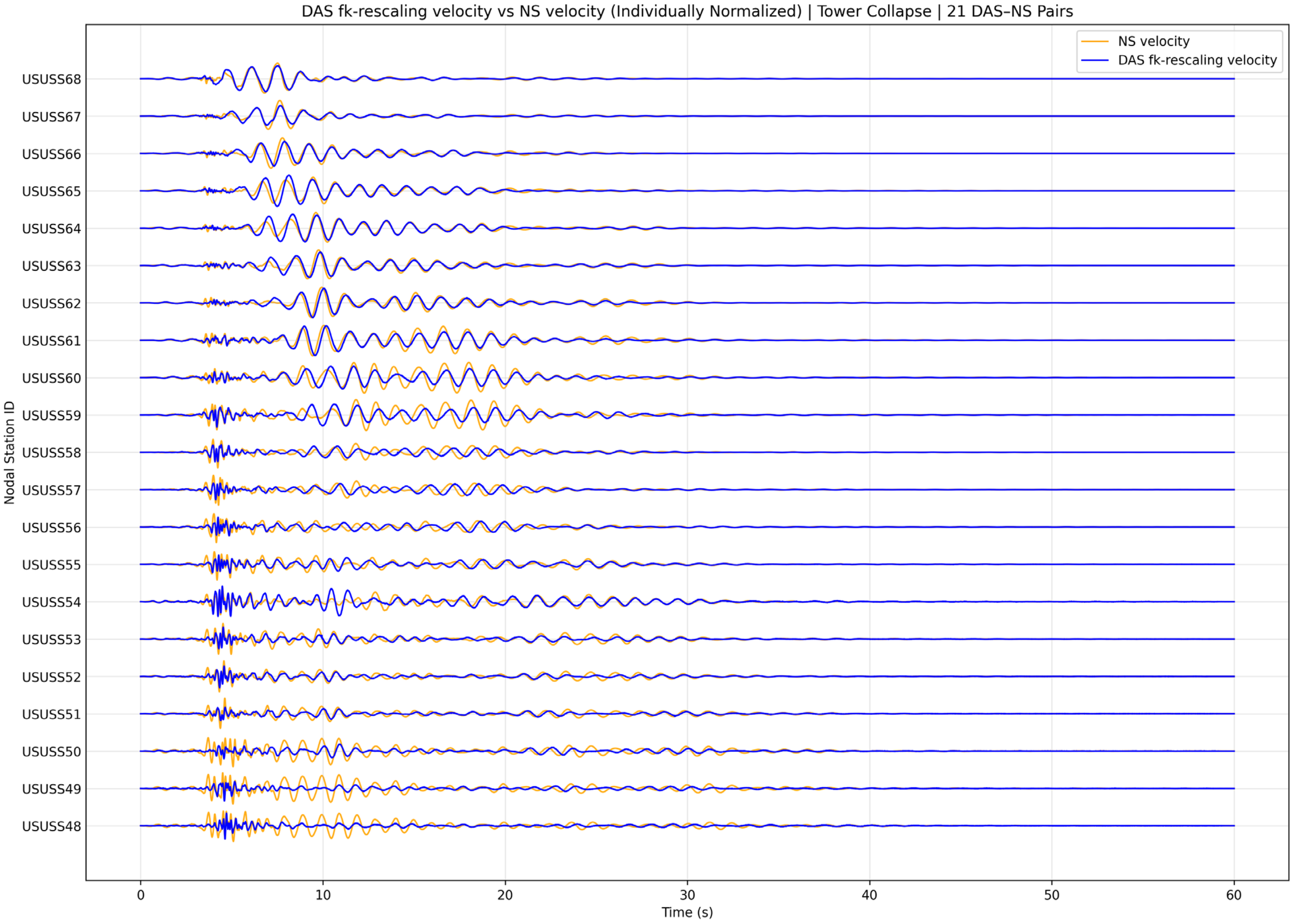

Figure S5: Waterfall-style comparison plots of particle velocity for DAS-NS pairs corresponding to nodal stations USUSS68-48 (starting from the pair closest to the tower-end) recorded during the tower collapse based on the fk-rescaling method.

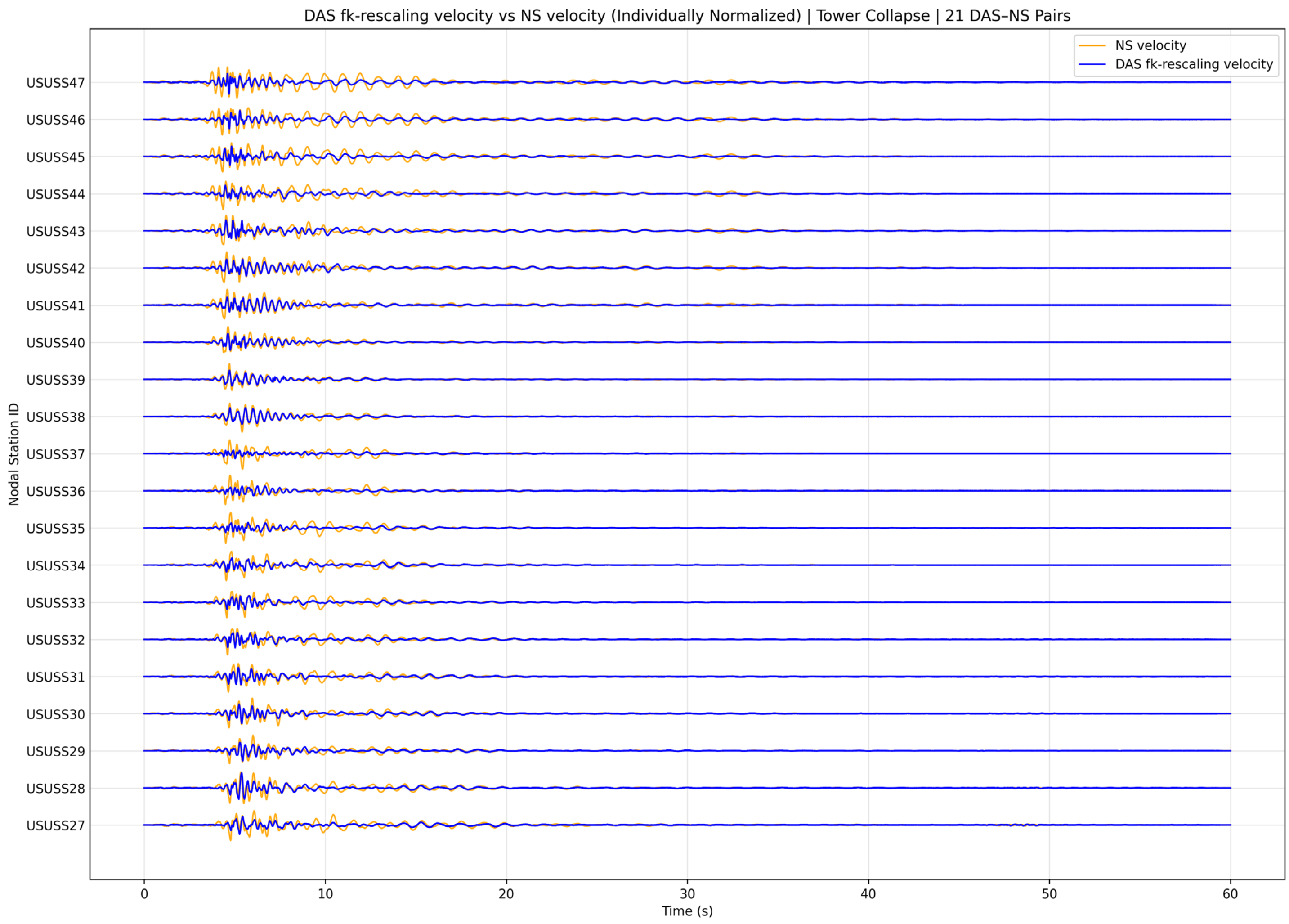

<u>Figure S6</u>: Waterfall-style comparison plots of particle velocity for DAS-NS pairs corresponding to nodal stations USUSS47-27 recorded during the tower collapse based on the fk-rescaling method.

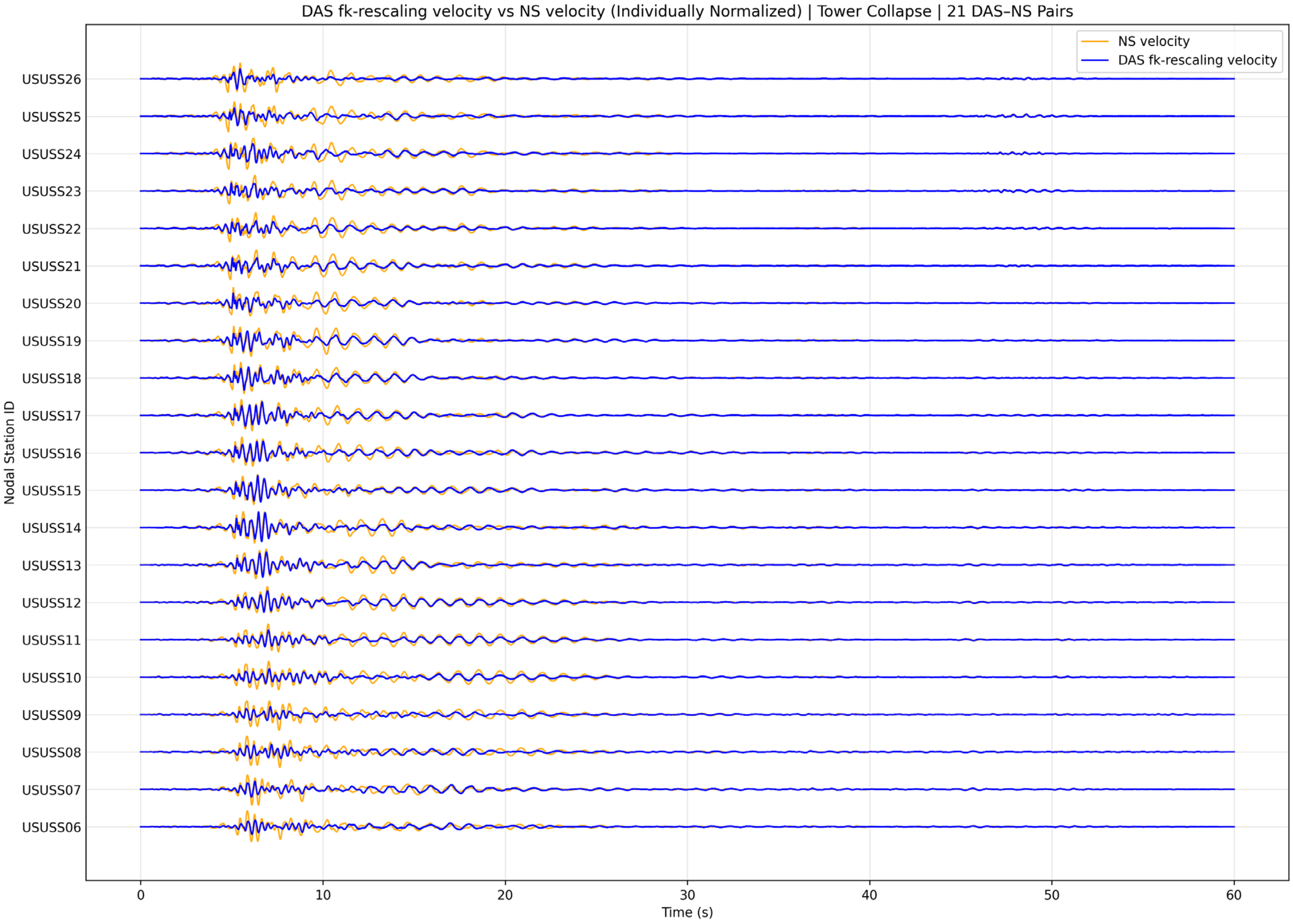

Figure S7: Waterfall-style comparison plots of particle velocity for DAS-NS pairs corresponding to nodal stations USUSS26-06 recorded during the tower collapse based on the fk-rescaling method.

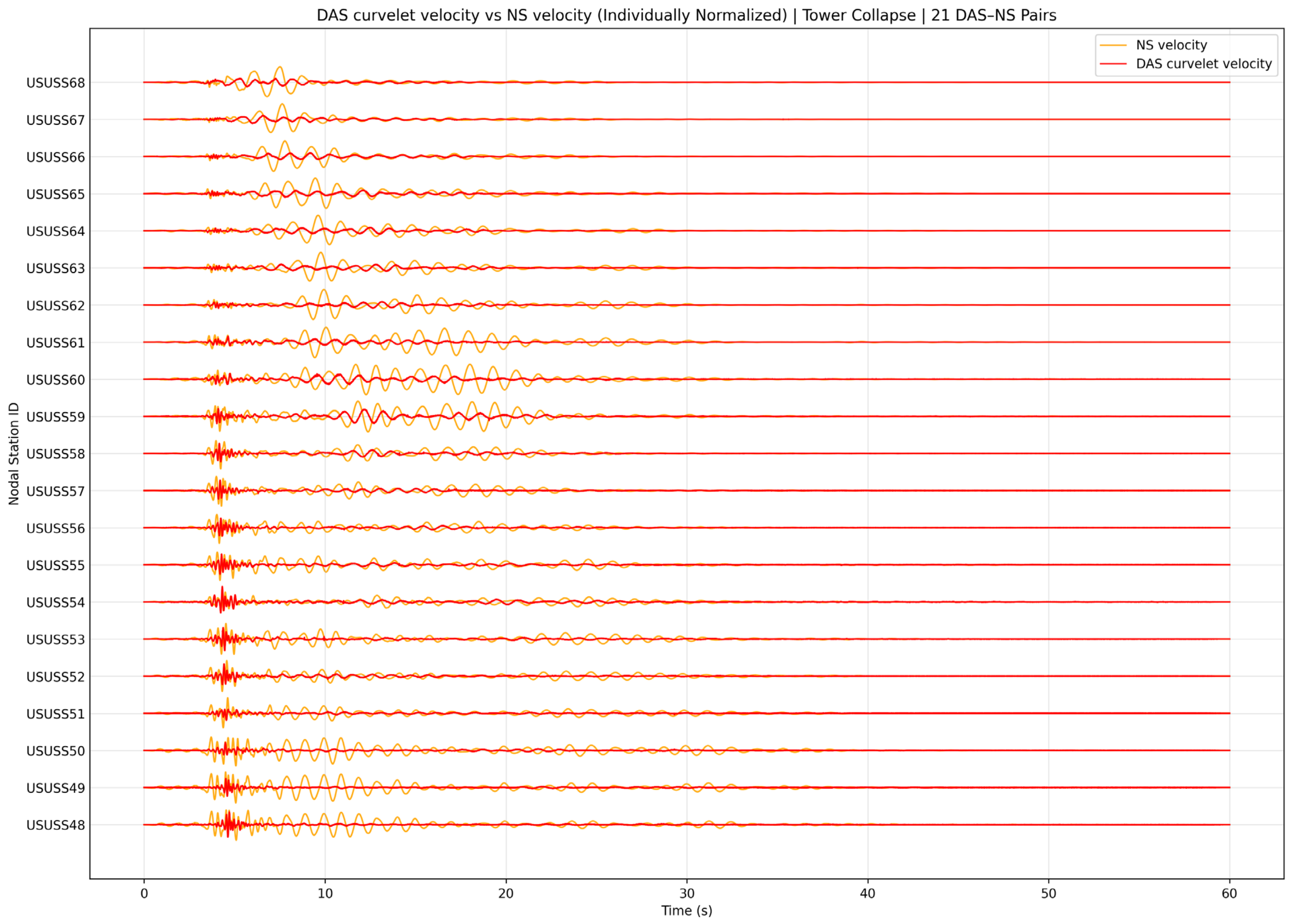

Figure S8: Waterfall-style comparison plots of particle velocity for DAS-NS pairs corresponding to nodal stations USUSS68-48 (starting from the pair closest to the tower-end) recorded during the tower collapse based on the curvelet method.

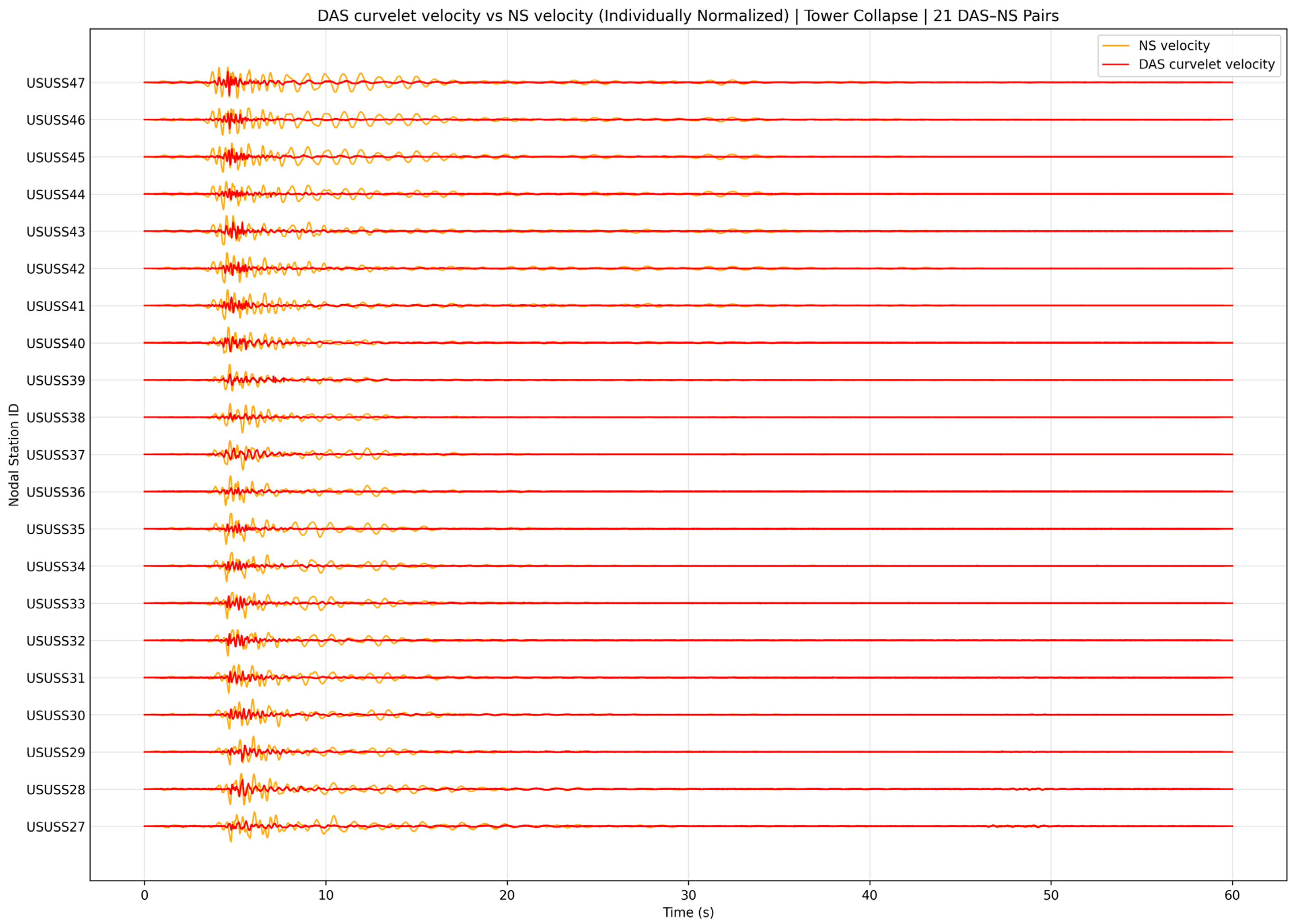

Figure S9: Waterfall-style comparison plots of particle velocity for DAS-NS pairs corresponding to nodal stations USUSS47-27 recorded during the tower collapse based on the curvelet method.

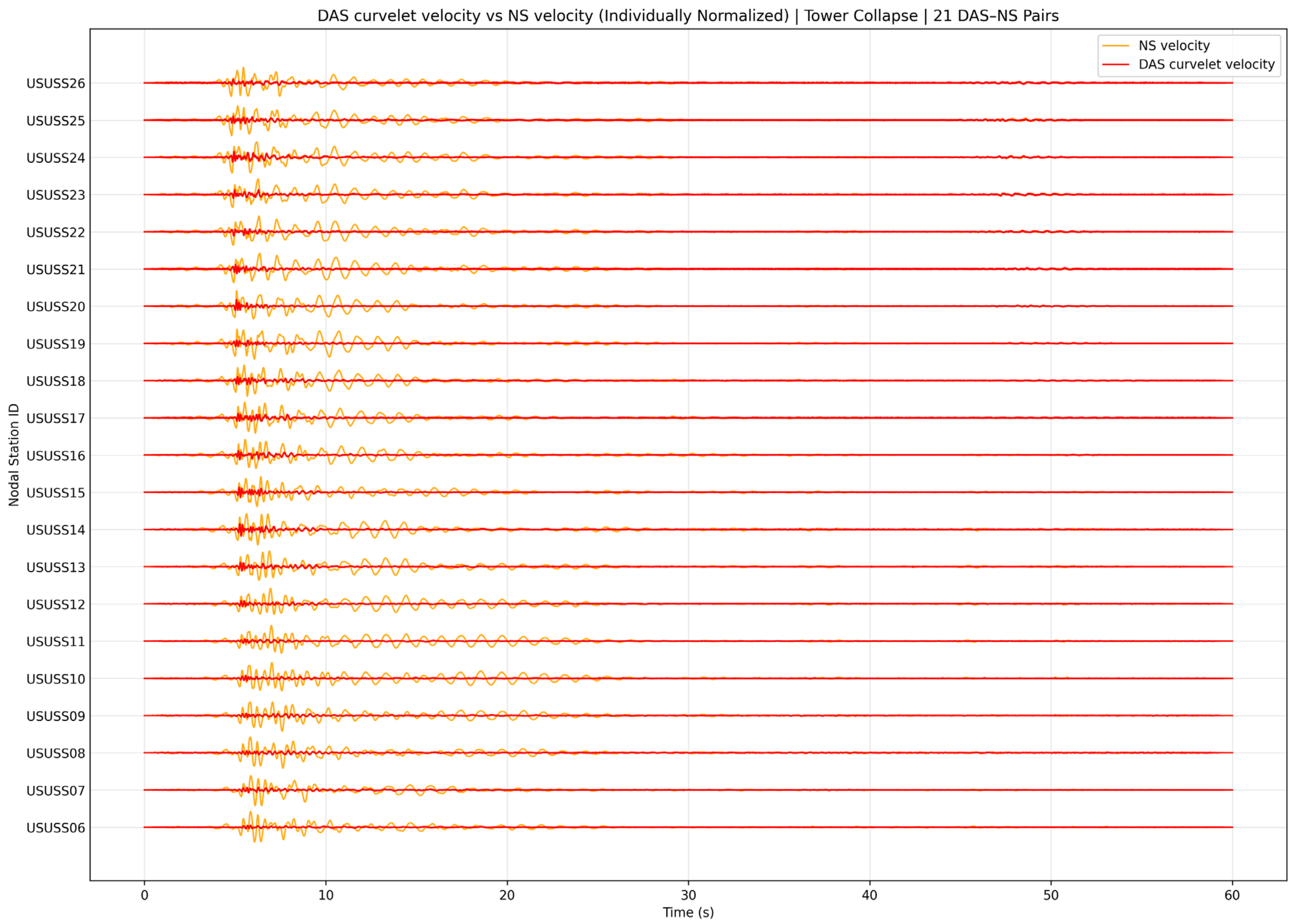

Figure S10: Waterfall-style comparison plots of particle velocity for DAS-NS pairs corresponding to nodal stations USUSS26-06 recorded during the tower collapse based on the curvelet method.

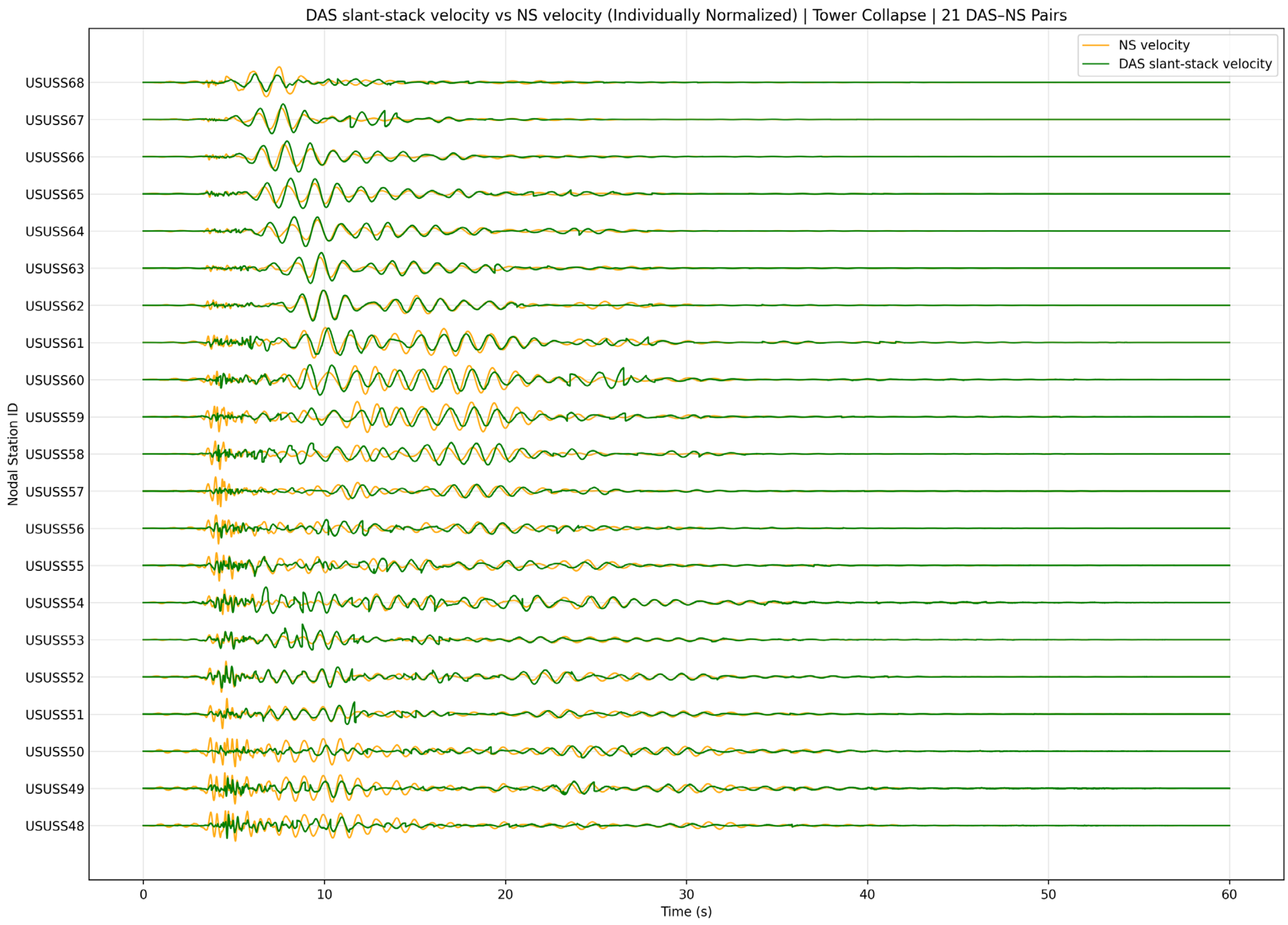

Figure S11: Waterfall-style comparison plots of particle velocity for DAS-NS pairs corresponding to nodal stations USUSS68-48 (starting from the pair closest to the tower-end) recorded during the tower collapse based on the slant-stack method.

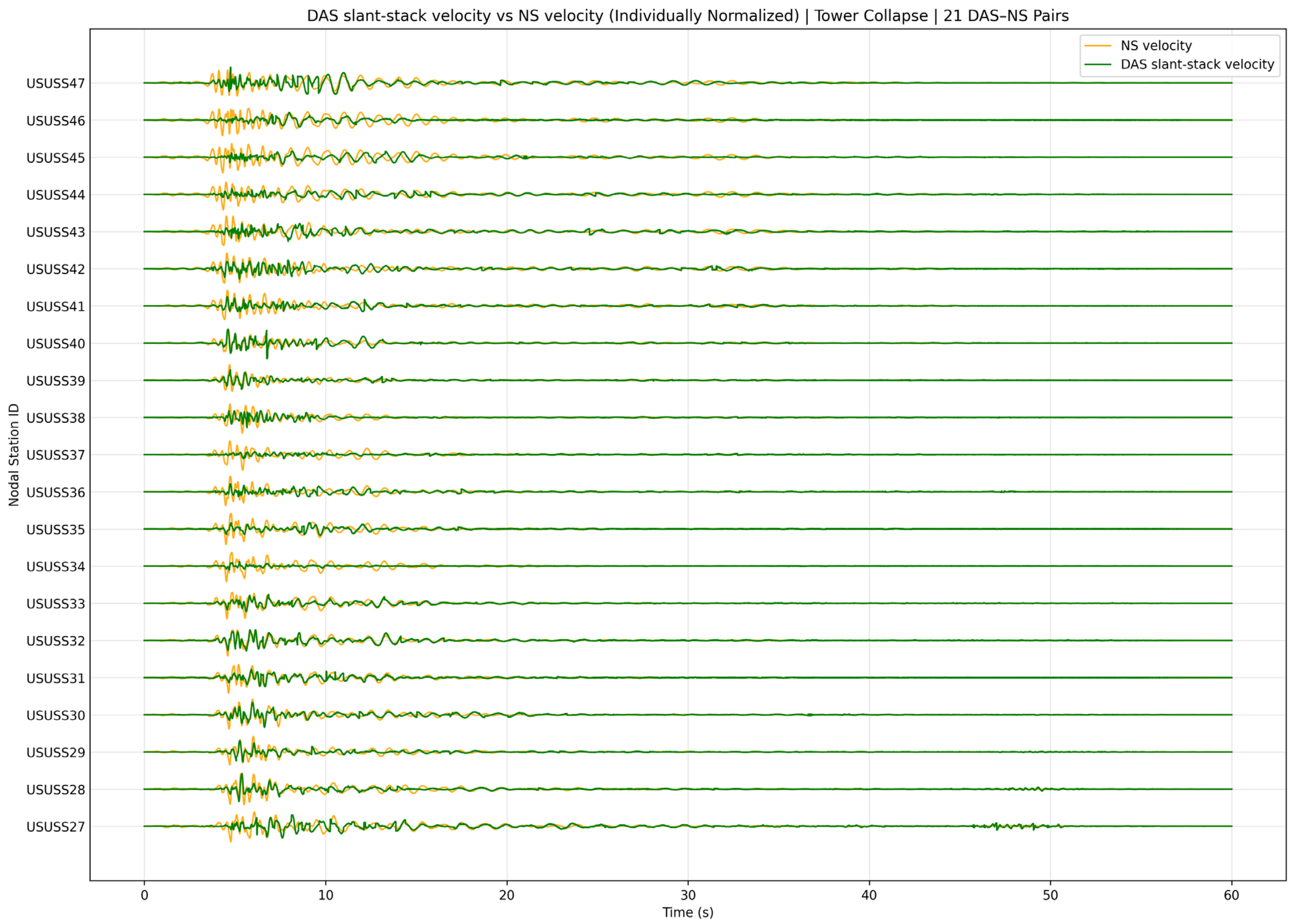

Figure S12: Waterfall-style comparison plots of particle velocity for DAS-NS pairs corresponding to nodal stations USUSS47-27 recorded during the tower collapse based on the slant-stack method.

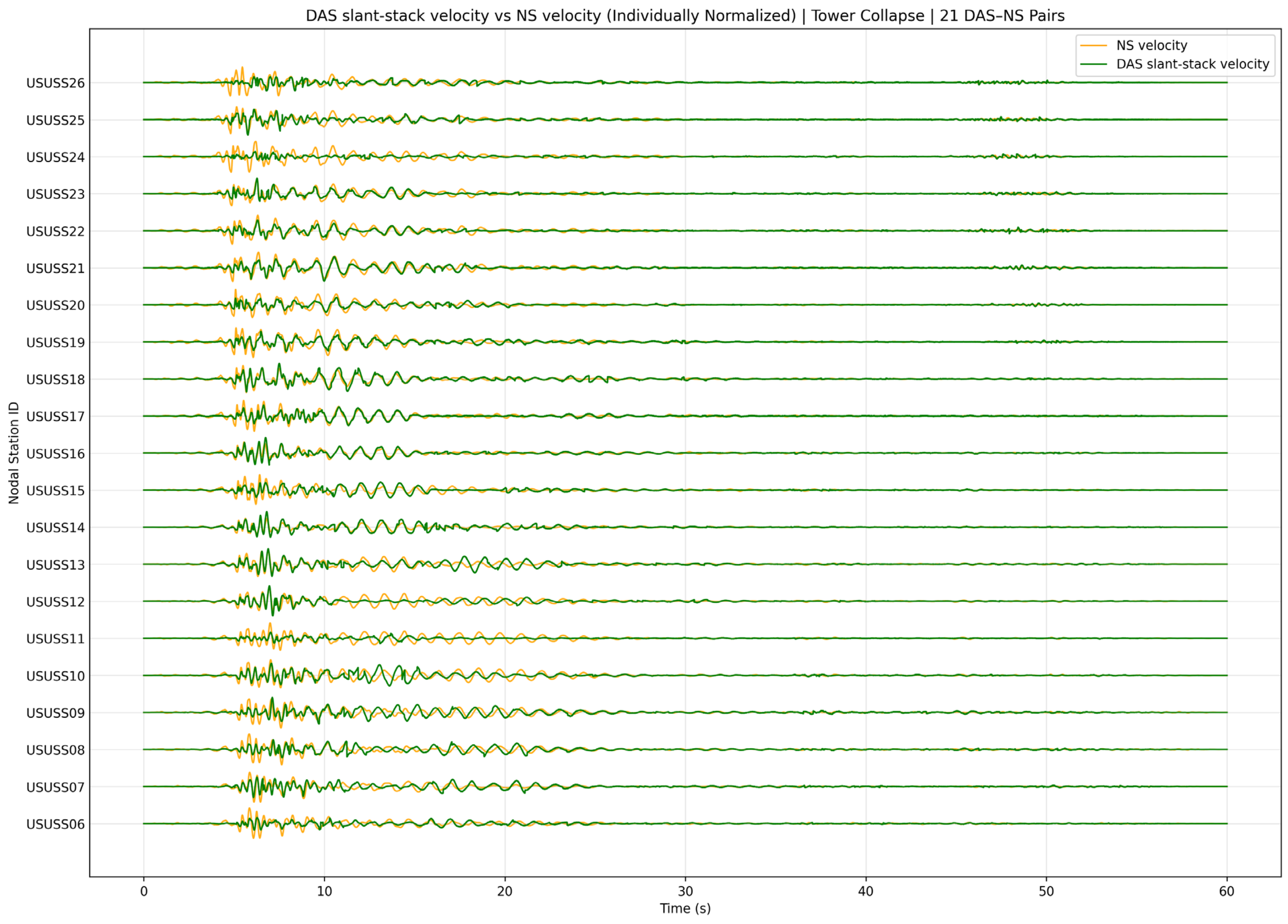

Figure S13: Waterfall-style comparison plots of particle velocity for DAS-NS pairs corresponding to nodal stations USUSS26-06 recorded during the tower collapse based on the slant-stack method.

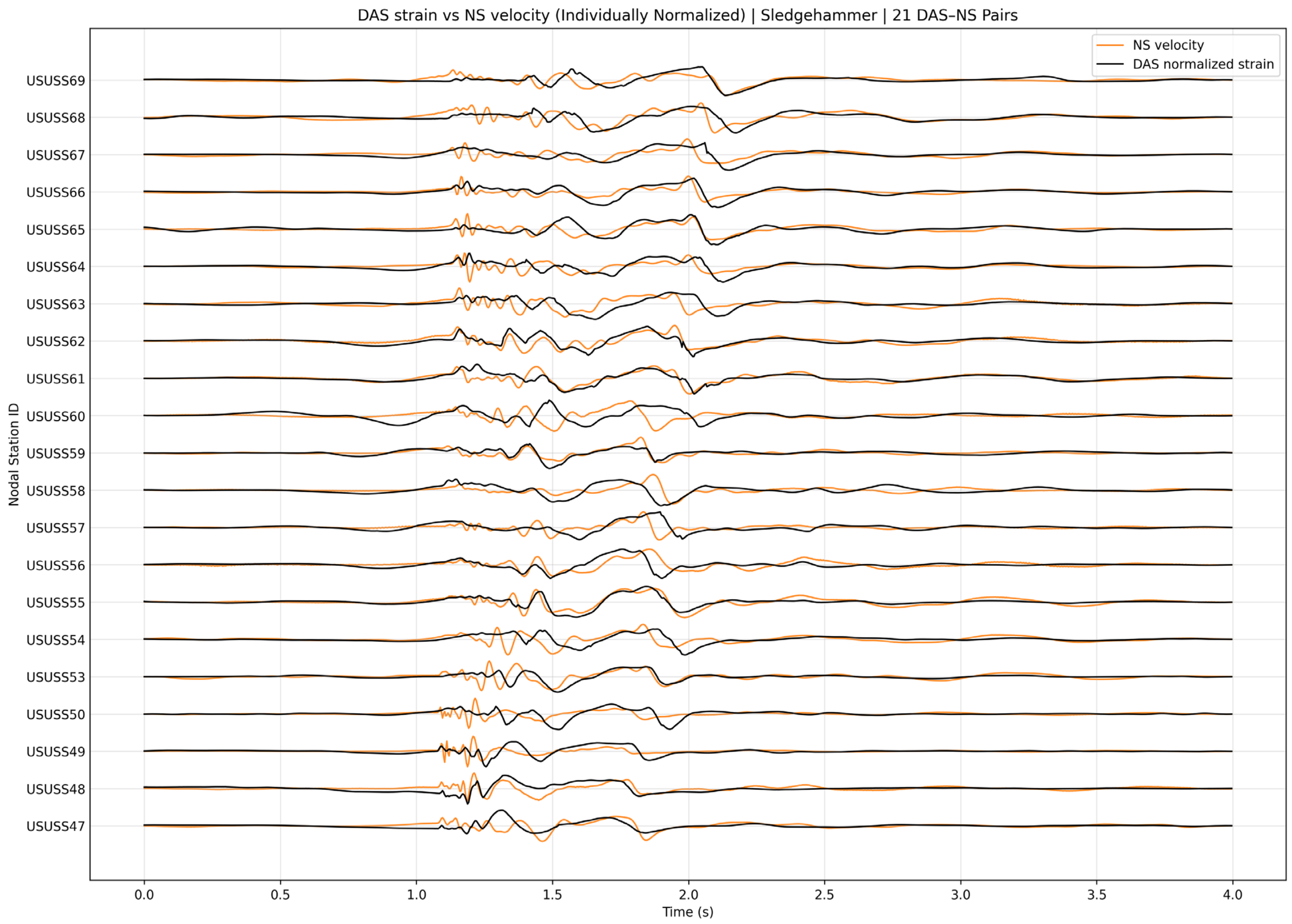

Figure S14: Waterfall-style comparison plots of normalized amplitude for DAS-NS pairs corresponding to nodal stations USUSS69-47 (starting from the pair closest to the tower-end) recorded during sledgehammer shots positioned 20 m away towards the tower-end of each pair.

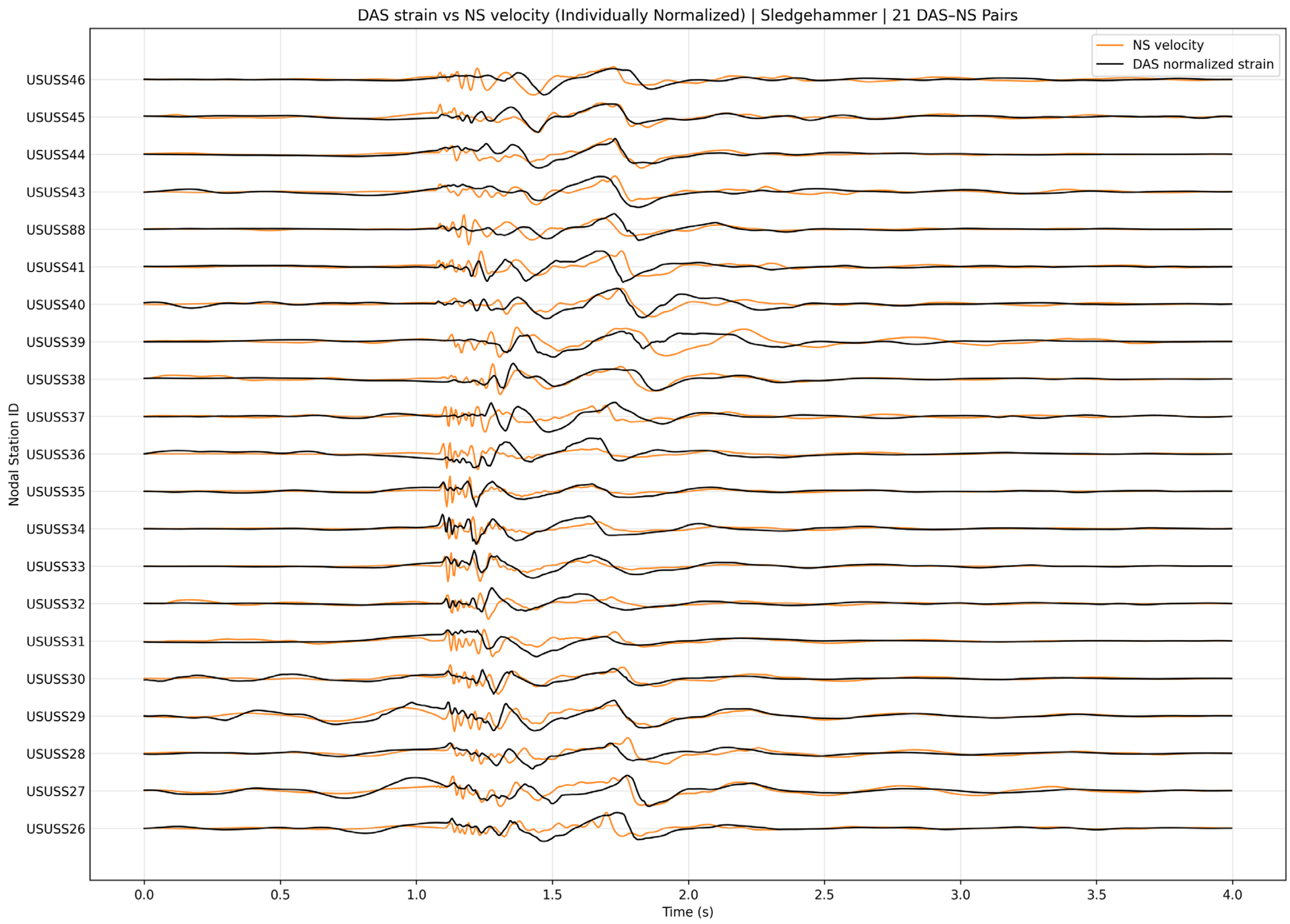

Figure S15: Waterfall-style comparison plots of normalized amplitude for DAS-NS pairs corresponding to nodal stations USUSS46-26 recorded during sledgehammer shots positioned 20 m away towards the tower-end of each pair.

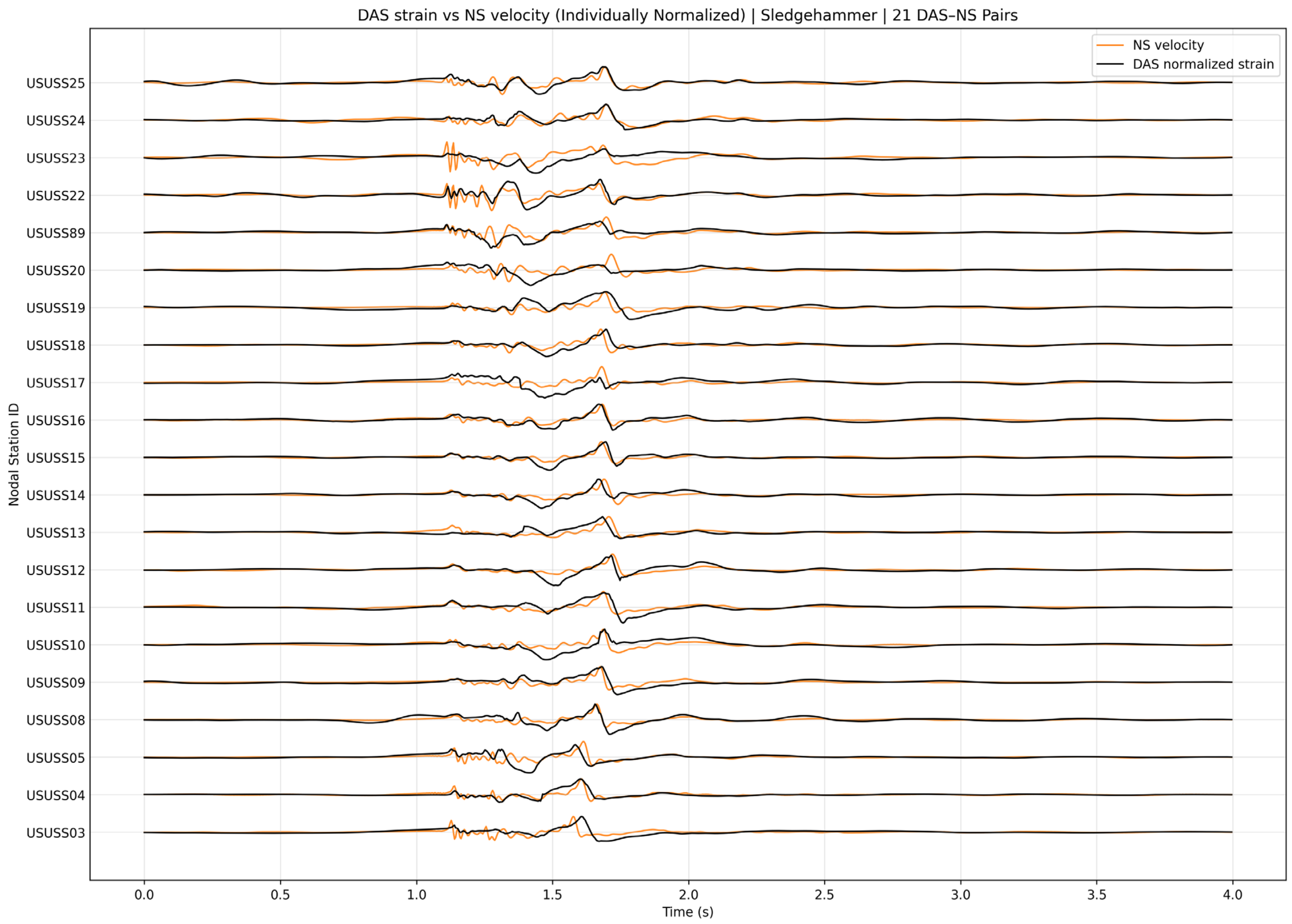

<u>Figure S16</u>: Waterfall-style comparison plots of normalized amplitude for DAS-NS pairs corresponding to nodal stations USUSS25-03 recorded during sledgehammer shots positioned 20 m away towards the tower-end of each pair.

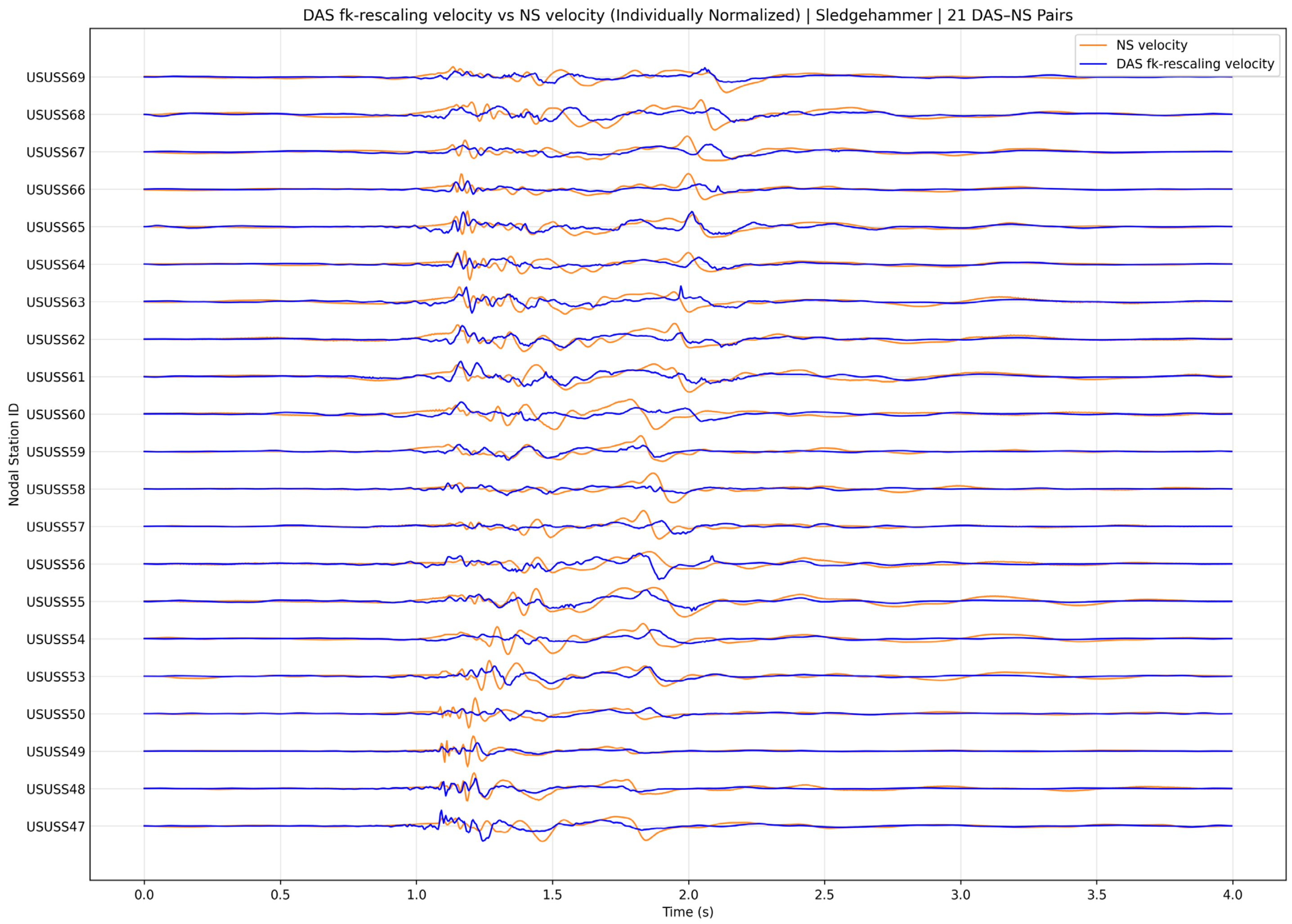

Figure S17: Waterfall-style comparison plots of particle velocity for DAS-NS pairs corresponding to nodal stations USUSS69-47 (starting from the pair closest to the tower-end) recorded during sledgehammer shots positioned 20 m away towards the tower-end of each pair based on the fk-rescaling method.

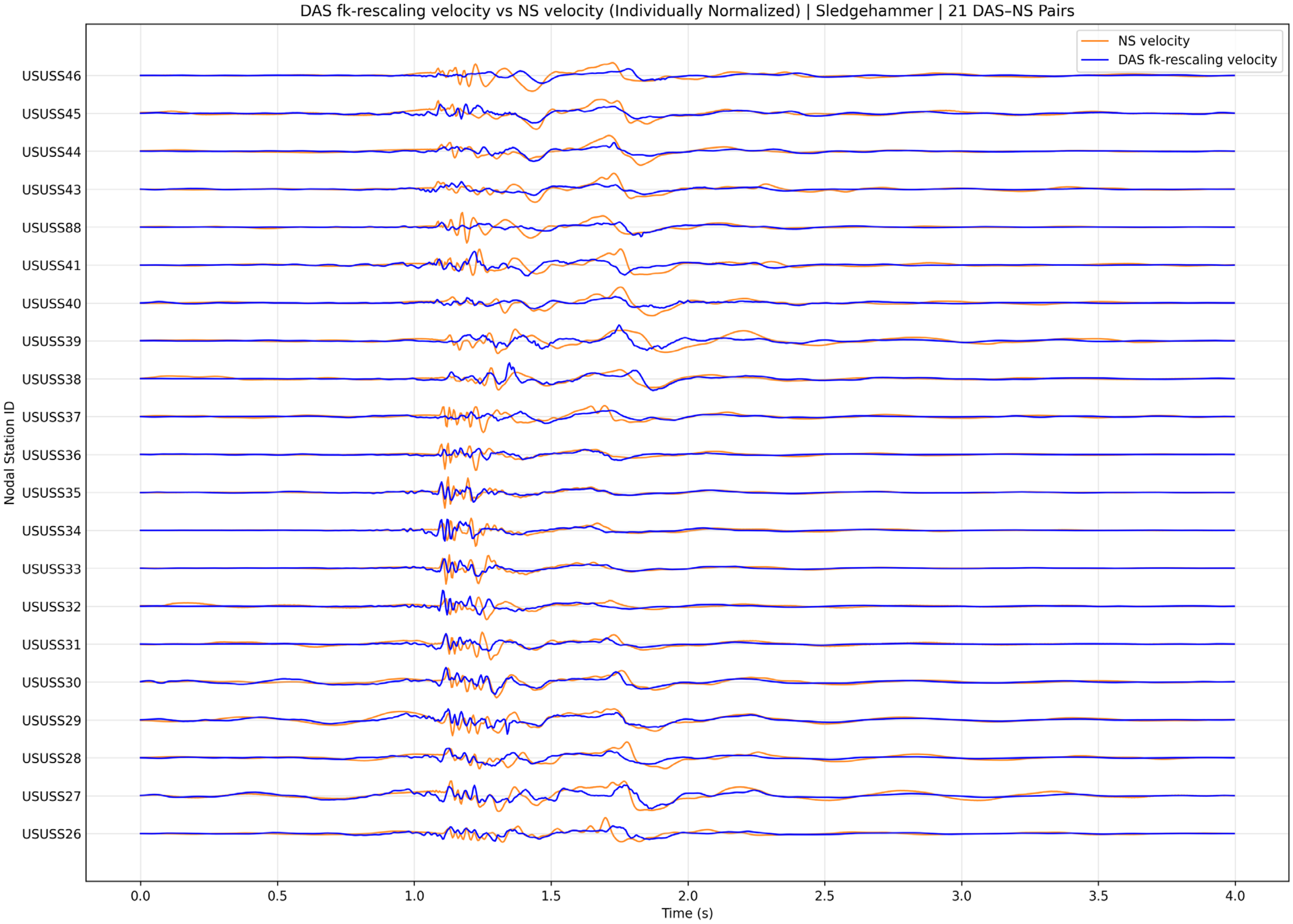

Figure S18: Waterfall-style comparison plots of particle velocity for DAS-NS pairs corresponding to nodal stations USUSS46-26 recorded during sledgehammer shots positioned 20 m away towards the tower-end of each pair based on the fk-rescaling method.

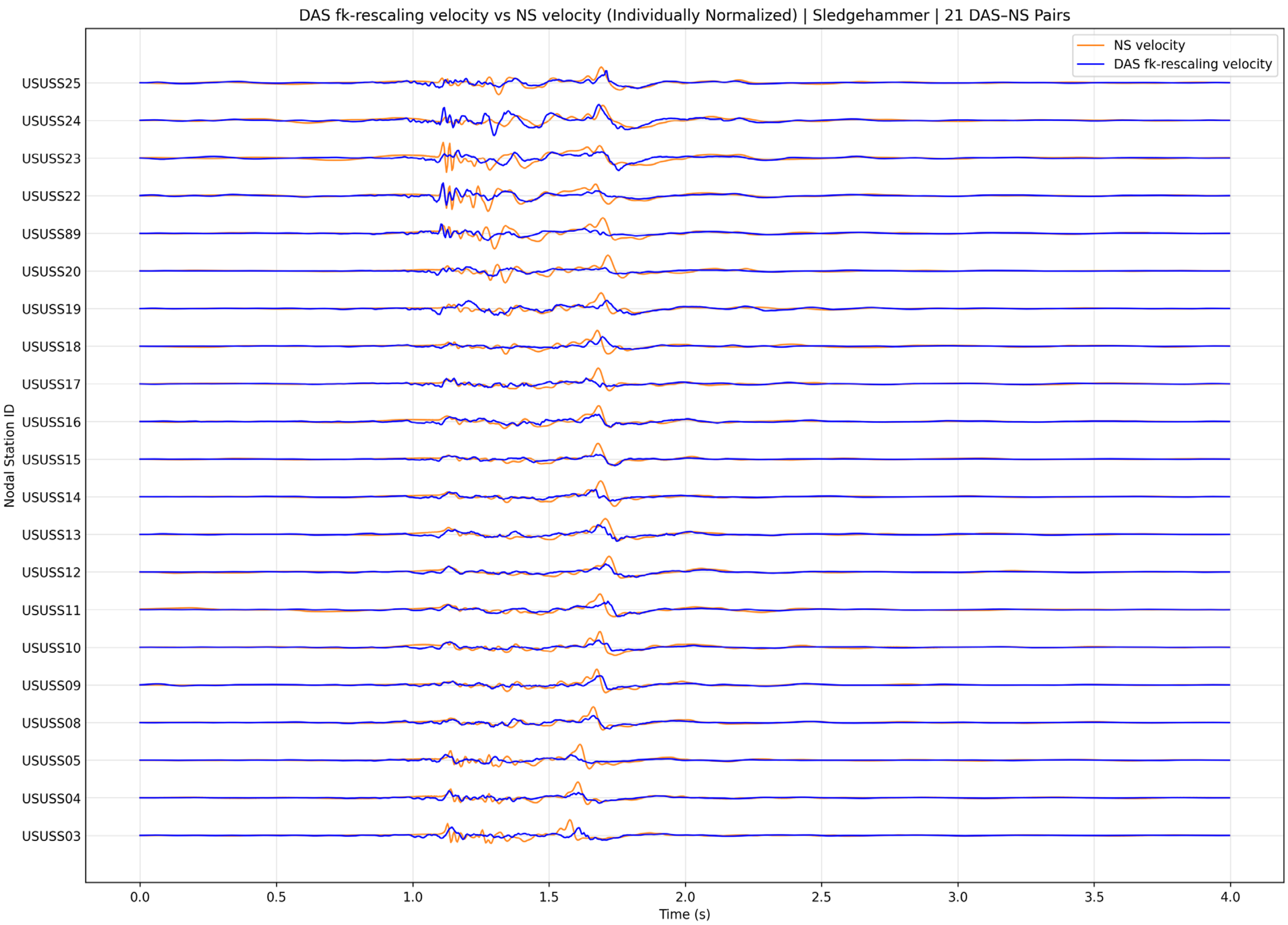

Figure S19: Waterfall-style comparison plots of particle velocity for DAS-NS pairs corresponding to nodal stations USUSS25-03 recorded during sledgehammer shots positioned 20 m away towards the tower-end of each pair based on the fk-rescaling method.

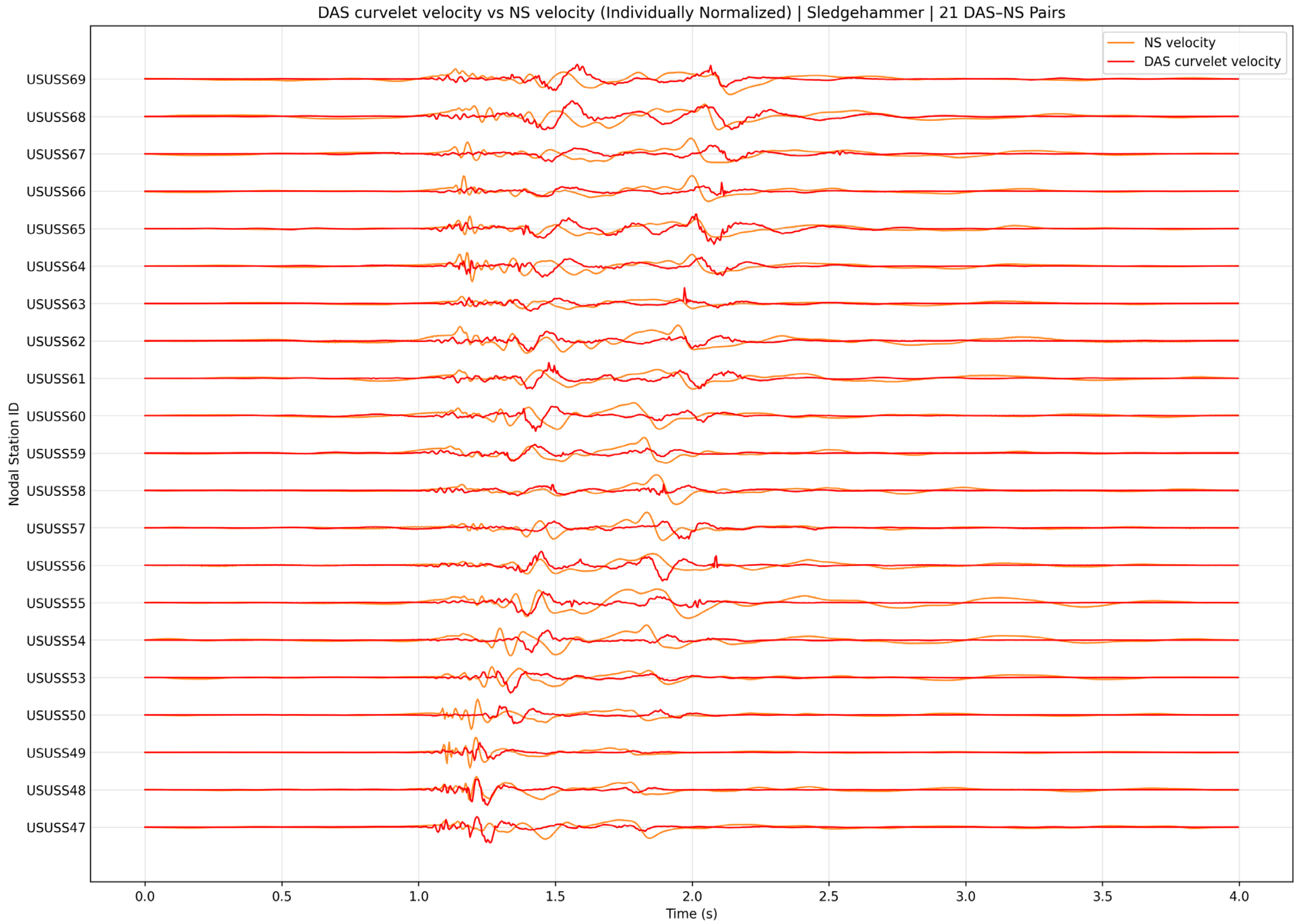

Figure S20: Waterfall-style comparison plots of particle velocity for DAS-NS pairs corresponding to nodal stations USUSS69-47 (starting from the pair closest to the tower-end) recorded during sledgehammer shots positioned 20 m away towards the tower-end of each pair based on the curvelet method.

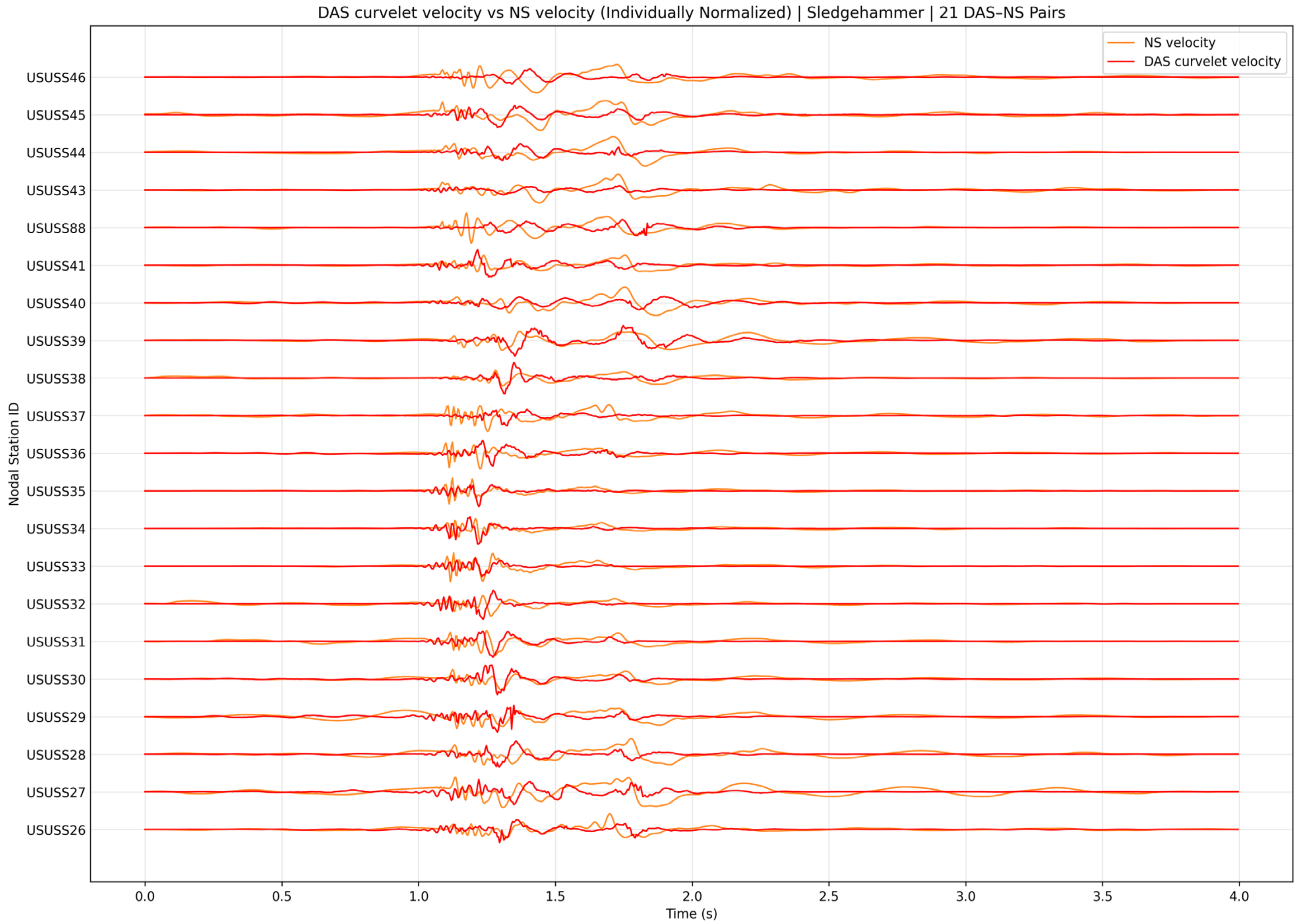

Figure S21: Waterfall-style comparison plots of particle velocity for DAS-NS pairs corresponding to nodal stations USUSS46-26 recorded during sledgehammer shots positioned 20 m away towards the tower-end of each pair based on the curvelet method.

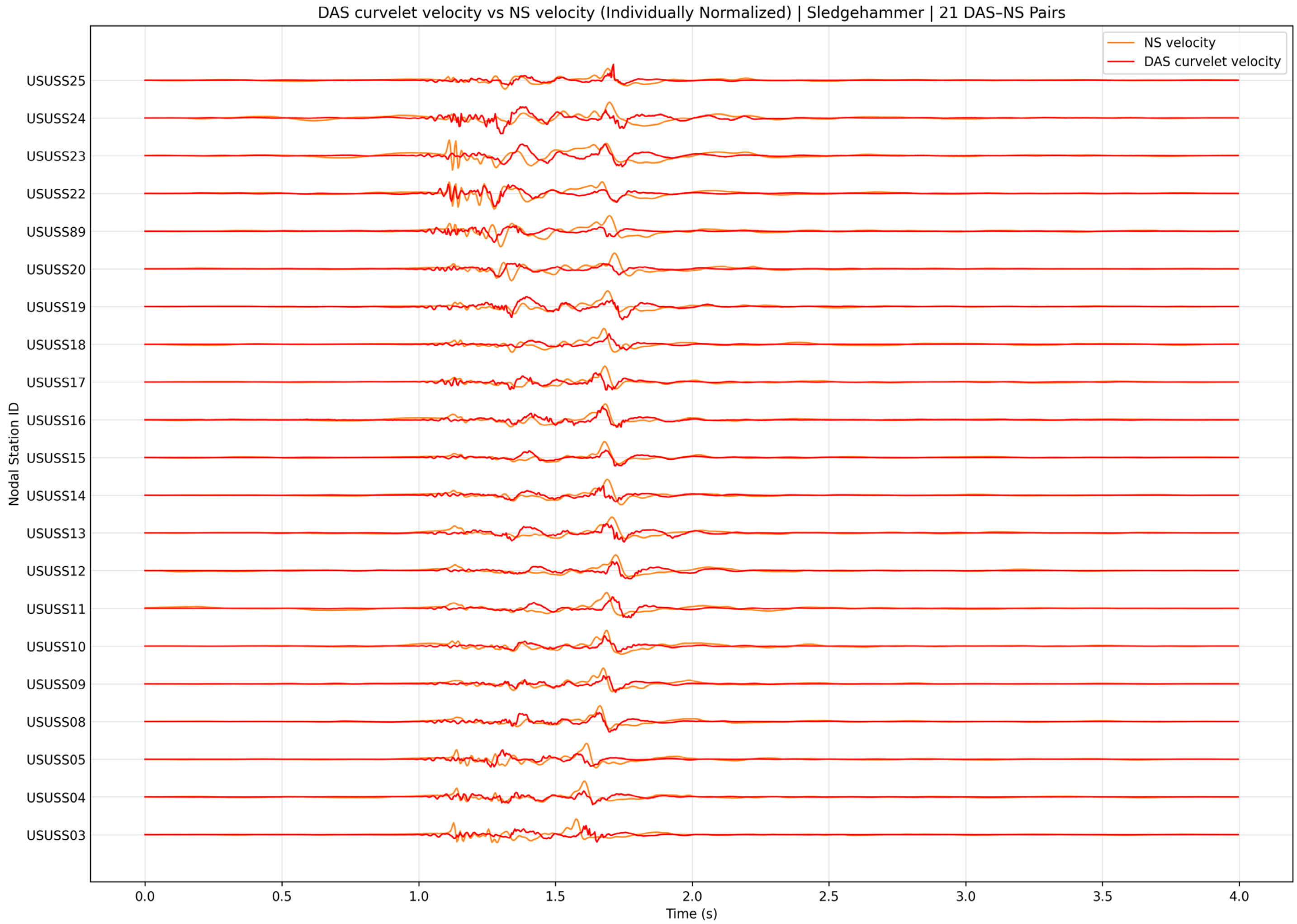

Figure S22: Waterfall-style comparison plots of particle velocity for DAS-NS pairs corresponding to nodal stations USUSS25-03 recorded during sledgehammer shots positioned 20 m away towards the tower-end of each pair based on the curvelet method.

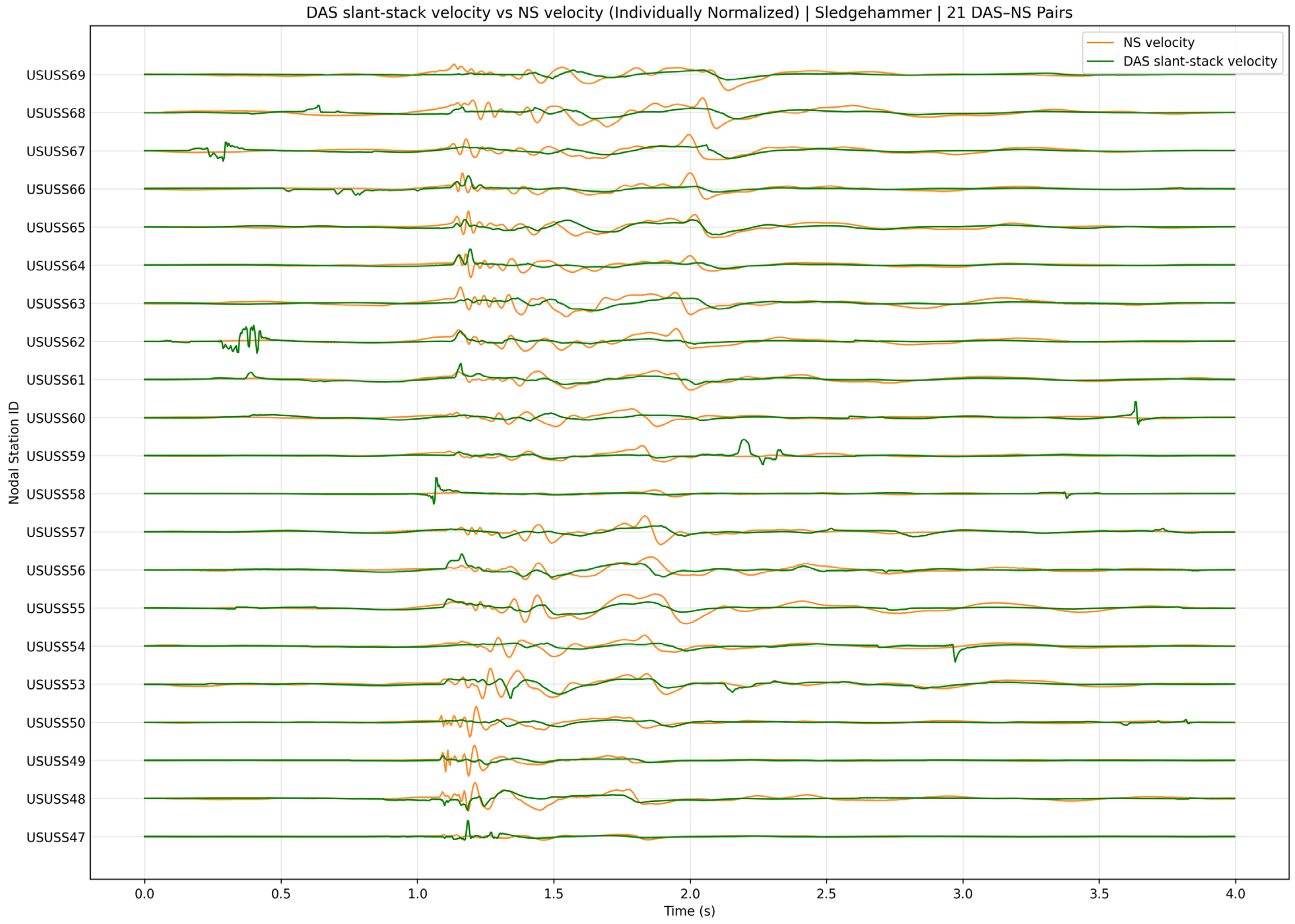

<u>Figure S23</u>: Waterfall-style comparison plots of particle velocity for DAS-NS pairs corresponding to nodal stations USUSS69-47 (starting from the pair closest to the tower-end) recorded during sledgehammer shots positioned 20 m away towards the tower-end of each pair based on the slant-stack method.

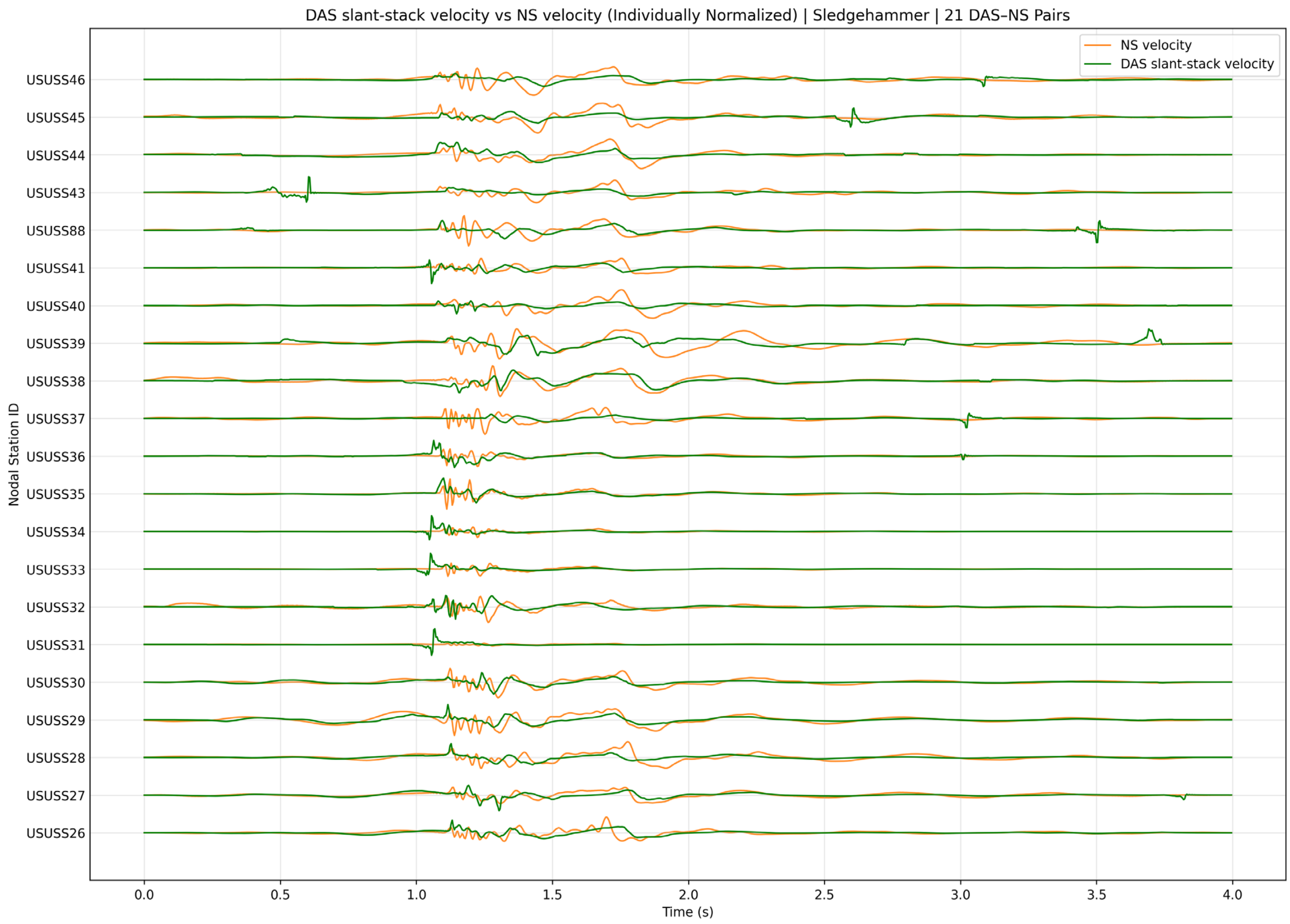

Figure S24: Waterfall-style comparison plots of particle velocity for DAS-NS pairs corresponding to nodal stations USUSS46-26 recorded during sledgehammer shots positioned 20 m away towards the tower-end of each pair based on the slant-stack method.

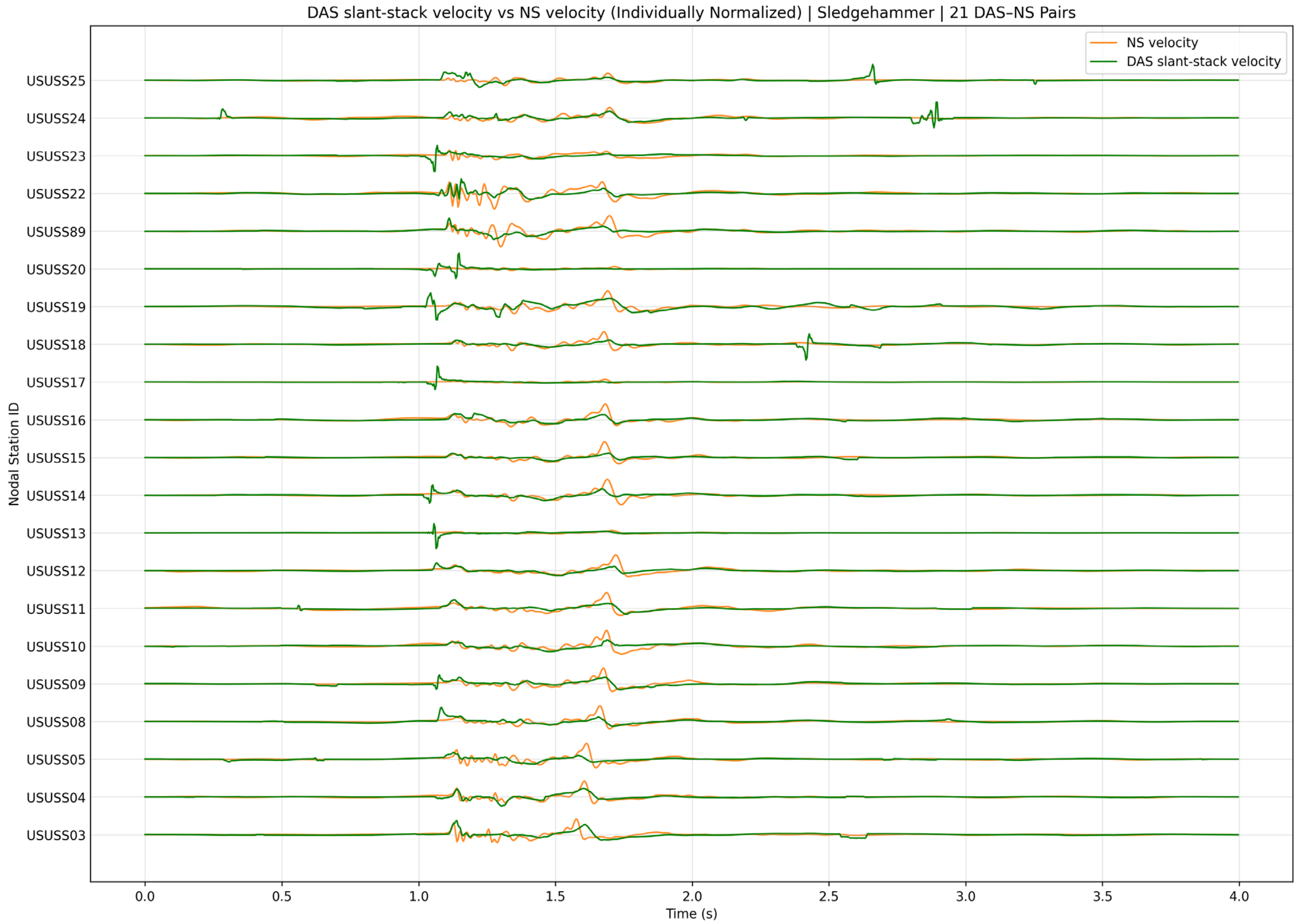

Figure S25: Waterfall-style comparison plots of particle velocity for DAS-NS pairs corresponding to nodal stations USUSS25-03 recorded during sledgehammer shots positioned 20 m away towards the tower-end of each pair based on the slant-stack method.

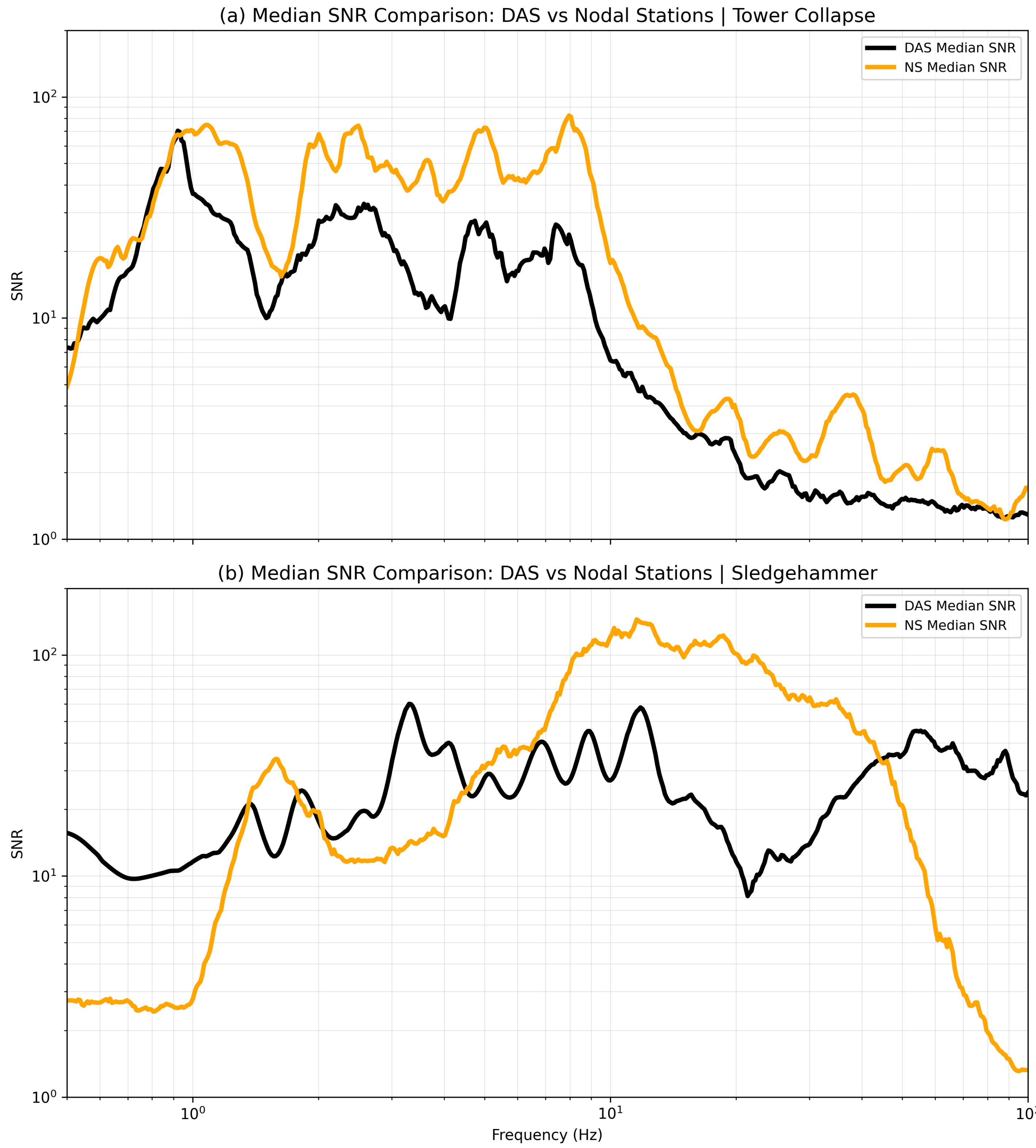

<u>Figure S26</u>: Comparison of median SNR plots for both DAS and NS waveforms over 0.5 – 100 Hz bandwidth for: (a) the tower collapse source, and (b) the sledgehammer source. Note that the same window lengths were used for the signal and noise portions when calculating SNRs for the tower collapse waveforms (60 s) and the sledgehammer waveforms (4 s).
